# Transcriptomic Analysis of the Regulation of Rhizome Formation in Temperate and Tropical Lotus (*Nelumbo nucifera*)

**DOI:** 10.1038/srep13059

**Published:** 2015-08-17

**Authors:** Mei Yang, Lingping Zhu, Cheng Pan, Liming Xu, Yanling Liu, Weidong Ke, Pingfang Yang

**Affiliations:** 1 Key Laboratory of Plant Germplasm Enhancement and Speciality Agriculture, Wuhan Botanical Garden, Chinese Academy of Sciences, Wuhan 430074, Hubei, China; 2 Key Laboratory of Aquatic Plant and Watershed Ecology, Wuhan Botanical Garden, Chinese Academy of Sciences, Wuhan 430074, Hubei, China; 3 University of Chinese Academy of Sciences, Beijing 100049, China; 4 National Garden of Aquatic Vegetable, Wuhan Institute of Vegetable Science, Wuhan 430065, Hubei, China

## Abstract

Rhizome is the storage organ of lotus derived from modified stems. The development of rhizome is a complex process and depends on the balanced expression of the genes that is controlled by environmental and endogenous factors. However, little is known about the mechanism that regulates rhizome girth enlargement. In this study, using RNA-seq, transcriptomic analyses were performed at three rhizome developmental stages—the stolon, middle swelling and later swelling stage —in the cultivars ‘ZO’ (temperate lotus with enlarged rhizome) and ‘RL’ (tropical lotus with stolon). About 348 million high-quality reads were generated, and 88.5% of the data were mapped to the reference genome. Of 26783 genes identified, 24069 genes were previously predicted in the reference, and 2714 genes were novel transcripts. Moreover, 8821 genes were differentially expressed between the cultivars at the three stages. Functional analysis identified that these genes were significantly enriched in pathways carbohydrate metabolism and plant hormone signal transduction. Twenty-two genes involved in photoperiod pathway, starch metabolism and hormone signal transduction were candidate genes inducing rhizome girth enlargement. Comparative transcriptomic analysis detected several differentially expressed genes and potential candidate genes required for rhizome girth enlargement, which lay a foundation for future studies on molecular mechanisms underlying rhizome formation.

*Nelumbo Adans*., a basal eudicot that belongs to the family of Nelumbonaceae, has a longer evolutionary history than most species of flowering plant. The genus is diploid (2*n* = 16) and its estimated genome size is 929 Mb^1^,^2^. *Nelumbo* comprises two extant species: *N. nucifera* Gaertn. and *N. lutea* (Willd.) Pers. *N. nucifera* is distributed throughout Asia and Northern Australia, whereas *N. lutea* is found in North America and northern of South America. These species differ in external morphology, such as plant size, leaf shape, petal shape and color^3^. *Nelumbo nucifera* can be classified into three categories according to their agricultural utilization and strongest feature: rhizomes, seed, and flower lotus. Rhizome lotus that are bred for rhizome quality have a large, edible rhizome but produce few flowers. Seed lotus has normally developed pistils, stamens and carpels, can flower profusely, and produce a high yield of nutrient-rich seeds. Flower lotus is primarily grown as ornamentals because of their attractive flower shape, petal colors, and highly variable number of petals. Compared with rhizome cultivars, seed and flower lotus have smaller rhizomes and more flowers^4^,^5^.

Rhizome is the storage organ of lotus derived from modified stems, and is used for asexual propagation which is the predominant propagation way in lotus. Moreover, rhizome is a popular edible vegetable. In China, the products of rhizome such as fresh, salted and boiled rhizomes are very popular in the daily diet because of its richness in nutrients including starch, proteins, vitamins, and mineral substances. For example, the fresh rhizome contains 10–20% starch^6^. With the unique characteristics, the rhizome forms underground, and elongates in a single direction after sprouting in the early spring with the formation of a few floating leaves from each node of the rhizomes. Subsequently, axillary rhizomes appear from each node in the main rhizomes which elongate underground forming many upright leaves. In late summer, rhizome stops longitudinal growth and starts to swell. Hence, rhizome become shorter and show increased girths, and some important carbohydrates such as starch are synthesized. Rhizomes and leaves continue developing under and above ground, respectively. In autumn, rhizome produces three or four enlarged internodes, and starch is accumulated rapidly to enable it survive the incoming winter^7^,^8^,^9^. In general, the development of rhizome internodes can be classified into four stages: stolon stage (elongate in a single direction), initial swelling (longitudinal growth stops and starts to increase girth), middle swelling (rhizomes continue swelling, and starch accumulate gradually), and later swelling stage (the enlargement of rhizome stop, and starch accumulate rapidly). Rhizome formation is a complex developmental process that initially leads to the formation of an underground stolon of longitudinal growth and then swells to enlarge girth^10^. The mechanisms that control rhizome development are poorly understood, because environmental factors, mainly photoperiod or temperature, strictly regulate the above four stages through triggering signal molecules or gene regulation^7^,^8^.

Development of storage organs have been extensively studied in some tuberous species such as *Solanum tuberosum* and *Begonia evansiana*^11^. Photoperiod regulates the formation of storage organ. Short photoperiod promotes the formation of storage organ, while long day prolongs this process. Photoperiodic control of tuberization shares a number of common elements with flowering regulation^12^. *Phytochrome B* involved in the response of plants to photoperiodic control, and the formation of storage organ is affected by *PHYB* under the short day (SD) condition^13^. Genes involved in flowering time regulation, *LOV KELCH PROTEIN 2* (*LKP2*), *CONSTANS* (*CO*), *GIGANTEA* (*GI*), and *FLOWERING LOCUS T* (*FT*), were believed to participate in the photoperiodic signal transduction, and promoted the transition of storage organ^14^,^15^. At the same time, the expression of miR172, which regulated photoperiodic control of floral transition by targeting a set of *APETALA2* (*AP2*)-like genes, also participated in the formation of underground storage organs^14^. Starch is considered as an important component in the storage organs. Accumulation of starch proceeds simultaneously with swelling of storage organs. *ADP-glucose pyrophosphorylase* (*AGPase*) and *granule-bound starch synthase* (*GBSS*) had been testified to affect the synthesis of starch in storage organs^16^,^17^,^18^. Hormones, especially gibberellic acid (GA) and abscisic acid (ABA), were involved in the initiation and growth of storage organs. Overexpression of GA oxidase gene could increase GA content, then promotes stolon elongation and inhibit storage organ formation^19^, while ABA induced tuber formation^20^. Moreover, *Patatin*, which is identical to glycoprotein, and some transcription factors, MADS-box transcription factors, is usually believed to be crucial for storage organ development^10^.

Similar to *Solanum tuberosum*, short day promotes rhizome girth enlargement in *Nelumbo*. Rhizome girth enlargement was brought about under 8, 10 and 12 h photoperiods, whereas rhizome elongated under 13 and 14 h photoperiods^7^. A transcriptional study investigated the expression of genes related to the development of rhizome. Fourteen important tuber formation-related genes were found to be involved in this enlargement^10^. However, rhizome girth enlargement is still poorly known in lotus. In order to reveal the mechanisms that control rhizome girth enlargement, we selected two lotus cultivars which differed in rhizome enlargement to conduct comparative transcriptomic analysis of the regulation of rhizome formation. The two cultivars are temperate lotus and tropical lotus, the two ecotypes of *N*. *nucifera*. The temperate lotus has an annual growth cycle, goes through the longitudinal growth of an underground stolon and induces swelling to enlarge girth. While the tropical lotus has no clear girth enlargement in the entire growth period, and thus cannot survive in the cold winter in the south–central regions of China such as Wuhan^4^,^21^,^22^. This difference in rhizome formation between the two ecotypes provides an opportunity to study the molecular mechanisms that control rhizome girth enlargement in lotus.

The present study focused on the identification of differentially expressed genes involved in rhizome formation during the rhizome development between two cultivars which differed in rhizome girth enlargement. We used the Illumina HiSeq^TM^ 2000 platform to sequence cDNA libraries from three stages of rhizome development: stolon (T1), middle swelling (T2), and late swelling (T3) stage for transcriptome sampling. These were studied in the lotus cultivars ‘ZO’ (Z, temperate lotus) and ‘RL’ (R, tropical lotus). This study should provide comprehensively understanding of the processes of rhizome formation and enlargement at transcriptional level.

## Results

### Rhizome development and sampling

**Figure 1:**
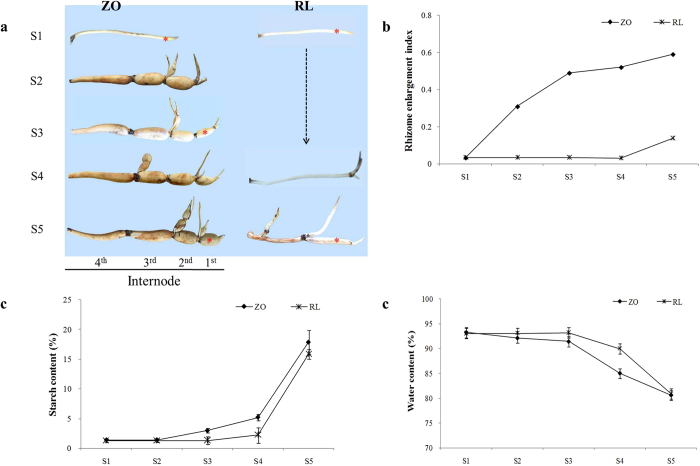
Rhizome development of ‘ZO’ and ‘RL’. (**a**) Changes in rhizome during the rhizome development of ‘ZO’, including five stages, stolon stage (S1), initial swelling (S2), middle swelling (S3 and S4), and later swelling (S5) stages for rhizome with the four internodes. The rhizomes for ‘RL’ were sampled at the corresponding day with ‘ZO’. The first internode at the stages S1, S3, and S5 are sampled for RNA-Seq and indicated with an asterisk, which named as T1, T2, and T3, respectively. (**b**) Rhizome enlargement index at different growth stages. (**c**) Starch content in the first internode of rhizomes at different growth stages. (**d**) Water content in the first internode of rhizomes at different growth stages.

Rhizome from ‘ZO’ and ‘RL’ were sampled at five stages, stolon stage (S1), initial swelling (S2), middle swelling (S3 and S4), and later swelling (S5) stages, throughout the growth season (Fig. 1a). Rhizome development was monitored by the measurement of rhizome enlargement index and starch content. Rhizome enlargement index of ‘ZO’ increased rapidly throughout the stage, ranged from 0.03 to 0.59. Rhizome enlargement index of ‘RL’ kept at 0.03 from S1 to S4, and increase to 0.14 at S5 (Fig. 1b). Starch content in the first internode of main rhizome of ‘ZO’ increased throughout the stage, with a slow rise from 1.4% to 3.0% from stage S1 to S4, and rapidly increased to 17.9% at S5. While, starch content of ‘RL’ kept at the low level (1.3%) from S1 to S4, and rapidly increased to 15.8% at S5 (Fig. 1c). Regardless of cultivars, in the first three stages water content reached up to 93%, then dropped rapidly to 80% at last two stages (Fig. 1d). Given our interest in the transcriptional changes that may be involved in regulating rhizome enlargement, we chose the rhizome at stages S1, S3, and S5 for RNA-seq. The first internodes from the three stages, which were named as T1, T2, and T3, of ‘RL’ and ‘ZO’ were sampled to construct six cDNA libraries: RT1, RT2, RT3, ZT1, ZT2, and ZT3.

### Illumina HiSeq mRNA sequencing

**Figure 2:**
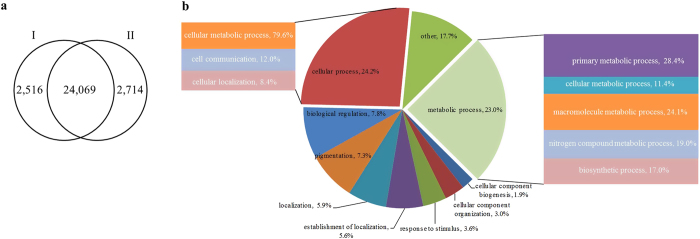
Novel transcript identified in the rhizome transcriptome. (**a**) Comparison of the estimated gene numbers between reference database (I) and our study (II). (**b**) GO annotation of the novel genes.

In total, 358 million short reads were generated from the six libraries, with 348 million high-quality (Q > 20) 100-bp reads selected for further analysis. 73.5–93.1% of the short clean reads were aligned against the ‘China Antique’ reference genome^2^. Among which, 75.3–85.9% of reads were mapped to exonic regions, 2.6–9.7% to intronic regions, and 11.5–17.6% to intergenic regions (Table 1). The reads from all the libraries were mapped to the lotus genome, and 26783 genes were identified (Fig. 2a). While 24832, 24832, 23871, 24926, 21937, and 22843 genes were identified in the RT1, RT2, RT3, ZT1, ZT2, and ZT3 libraries, respectively (Table 1).

Moreover, 24069 out of 26783 genes were previously predicted in the lotus reference (Fig. 2a). The remaining 2714 genes were not included in the reference genes, representing novel transcripts (Fig. 2a). Among these novel genes, 1015 could represent different alleles of 1823 reference genes, which was based on the BLASTN alignment with E-value < 10^−5^ and identity ≥ 0.73 (Table S2). As the complement to the lotus reference genome^2^, the 2714 novel genes were named as NNU_26687—NNU_29400. The 2516 reference genes were not found in the rhizome transcriptome (Fig. 2a), which also had no or low expression in the rhizome of the reference lotus^2^. Of the 2714 novel transcripts, 1186 genes were successfully annotated by GO assignments, and were classified into 9 sub-categories in biological process (Fig. 2b). The annotated sequences were included in cellular process (24.2%), metabolic process (23.0%), biological regulation (7.8%), pigmentation (7.3%), localization (5.9%), establishment of location (5.6%), response to stimulus (3.6%), cellular component organization (3.0%), cellular component biogenesis (1.9%) and other categories (17.7%). For cellular process, cellular metabolic process (79.6%) was the most enriched term. For metabolic process, primary metabolic process (28.4%) and macromolecule metabolic process (24.1%) were the two overrepresented terms (Fig. 2b).

### Differentially expressed genes (DEGs) during rhizome development

**Figure 3:**
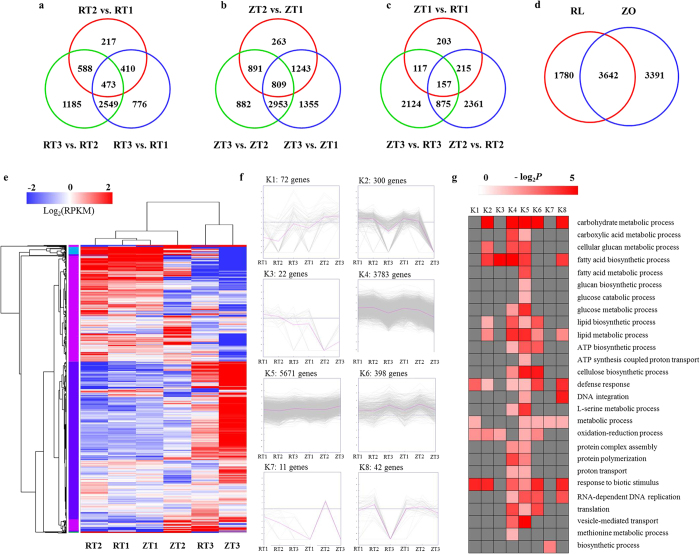
Overview of serial analysis of DEGs identified by pairwise comparisons of the six rhizome transcriptomes, RT1, RT2, RT3, ZT1, ZT2, and ZT3. (**a**) Venn diagram of DEGs in ‘RL’ at three stages. (**b**) Venn diagram of DEGs in ‘ZO’ at three stages. (**c**) Venn diagrams of DEGs between ‘RL’ and ‘ZO’ at the particular stage. The individual and overlapping areas in venn diagrams represent the number of specifically expressed and co-expressed genes between different stages. (**d**) Number of DEGs identified in ‘RL’ and ‘ZO’. (**e**) Heatmap of DEGs across three rhizome developmental stages in the lotus cultivars, ‘RL’ and ‘ZO’. Expression values of six libraries are presented as RPKM normalized log2 transformed counts. Red and blue colors indicate up- and down- regulated transcripts, respectively. Eight main clusters are shown. (**f**) Expression patterns of the genes in the eight main clusters, namely K1-K8, corresponding to the heatmap. (**g**) GO-term function enrichment analysis of different clusters. The significances of the most represented GO-slims in each main cluster are indicated using log-transformed P-value (red). The dark grey areas represented the missing values.

Differences in gene expression at three stages during rhizome development in two cultivars were examined, and DEGs were identified by pairwise comparisons of the six libraries (Fig. 3). Comparisons of the three stages of ‘RL’ identified 1688, 4208, and 4795 DEGs in pairs of RT2 vs. RT1, RT3 vs. RT1, and RT3 vs. RT2. Comparisons of the three stages of ‘ZO’ identified 3206, 6360, and 5535 DEGs in pairs of ZT2 vs. ZT1, ZT3 vs. ZT1, and ZT3 vs. ZT2. The total number of DEGs across the three stages was much higher in ‘ZO’ compared with ‘RL’ at each stage (Fig. 3a,b). The number of DEGs detected in same-stage comparisons between the two lotus cultivars was generally lower than that detected from same-cultivar comparisons at different stages (Fig. 3c). In total, 10299 genes were differentially expressed for ‘RL’ and ‘ZO’ at three stages. Of these DEGs, 3642 were significantly regulated in both cultivars during rhizome development, 1780 and 3399 genes were differentially regulated in ‘RL’ and ‘ZO’, respectively (Fig. 3d). This indicated that more DEGs participated in the rhizome formation of ‘ZO’. While the rest DEGs were differentially expressed between the two cultivars.

We performed hierarchical clustering of all DEGs using the euclidean distance method associated with complete-linkage (Fig. 3e,f). Eight clusters were plotted with expression patterns. The K1 cluster include 78 genes which showed up-regulation in both cultivars across the stage proceeded, and the expression level of these genes in ‘RL’ was lower than in ‘ZO’. Most of the genes in K2 cluster were down-regulated in both cultivars with the developmental stage going. The 22 gene in K3 were down-regulated in both cultivars, and the expression level in ‘RL’ was higher than in ‘ZO’. The genes in clusters K4 and K5 possessed most of DEGs, but the genes in the two clusters had opposite expression pattern. The genes in K4 were significantly down-regulated at T3 stage compared to the T1 and T2 stages in both cultivars, whereas, the ones in K5 were significantly up-regulated at T3 stage compared to the first two stages in both cultivars. The K6 cluster is composed of 398 genes. The expression of these genes was up-regulated at T3 stage compared to the first two stages in ‘RL’, but was the highest at T2 stage in ‘ZO’. The 11 genes in K7 were down-regulated in ‘RL’ with the developmental stage going, and were expressed merely in the T2 stage in ‘ZO’. The genes in K8 were up-regulated at the T2 stage compared to T1, and then down-regulated at T3 stage in both cultivars.

### Functional classification of DEGs during rhizome development

We used GO assignments to classify the functions of DEGs in pairwise comparisons of cDNA libraries between different cultivars and between different rhizome developmental stages (Fig. S1). In the three GO categories (biological process, cellular component, and molecular function), no GO terms were significantly enriched in the comparisons of ZT3 vs. ZT1 and ZT1 vs. RT1. In the biological process category, ‘carbohydrate metabolic process’, and ‘glucan metabolic process’ were significantly enriched (*P* < 0.05) in other seven pairwise comparisons. In the cellular component category, no GO terms were significantly enriched in all pairwise comparisons, but ‘apoplast’ was significantly enriched in the RT3 vs. RT1, RT3 vs. RT2, and ZT2 vs. ZT1. In the molecular function category, four GO terms ‘cation binding’, ‘hydrolase acticity’, ‘oxidoreductase activity’, and ‘xyloglucan:xyloglucosyl transferase activity’ were significantly enriched in most of comparisons.

We further analyzed the overrepresented GO functions within each cluster. The enriched GO terms of biological process are showed in Fig. 3g. The K4 and K5 clusters contained the most overrepresented GO terms among all of the clusters. Genes involving ‘carbohydrate metabolic process’, ‘glucan metabolic process’, and ‘fatty acid biosynthetic process’ were greatly enriched, although the genes exhibited opposite regulatory patterns in these two clusters. Some genes GO terms were enriched in particular cluster, such as ‘fatty acid metabolic process’, ‘glucan biosynthetic process’, and ‘glucan catabolic process’ were enriched specifically in K5. To further explore the biological pathways that were involved in the differentially expressed genes, we performed KEGG analysis of DEGs. Dozens of genes were assigned to the biosynthesis of secondary metabolites, starch and sucrose metabolism, carbon metabolism, flavonoid biosynthesis, and biosynthesis of amino acids processes (Fig. S2).

### Genes related to rhizome development

Genes involved in photoperiod pathway, starch biosynthesis, and hormone signal transduction are reported to be related to rhizome enlargement in the previous study. The genes in photoperiod pathway that were related to rhizome formation are *PHYB*, *CO*, *GI*, and *FT*. Putative homologs of these genes were identified in lotus rhizome (Table 2). *PHYB*, which prevents tuberization, had the highest expression at the T2 stage in ‘RL’, but had the lowest expression at the T2 stage in ‘ZO’. Two homologs of *GI*, NNU_10096 and NNU_28780 (novel gene), had the lowest expression at the T2 stage in ‘RL’, but in ‘ZO’ two homologs had an opposite expression pattern. The putative homologs of members of the *CO* gene family, including *COL1*–*COL5*, *COL7*, *COL9*–*COL10*, and *COL13*–*COL15*, *COL16*, were found to have different expression patterns. The three homologs of *FT*, NNU_15765, NNU_20154, and NNU_26362, were identified in rhizome transcriptome. NNU_20154 and NNU_26362 were down-regulated in the two cultivars during the rhizome development, while NNU_015765 showed opposite expression patterns between ‘RL’ and ‘ZO’.

**Figure 4:**
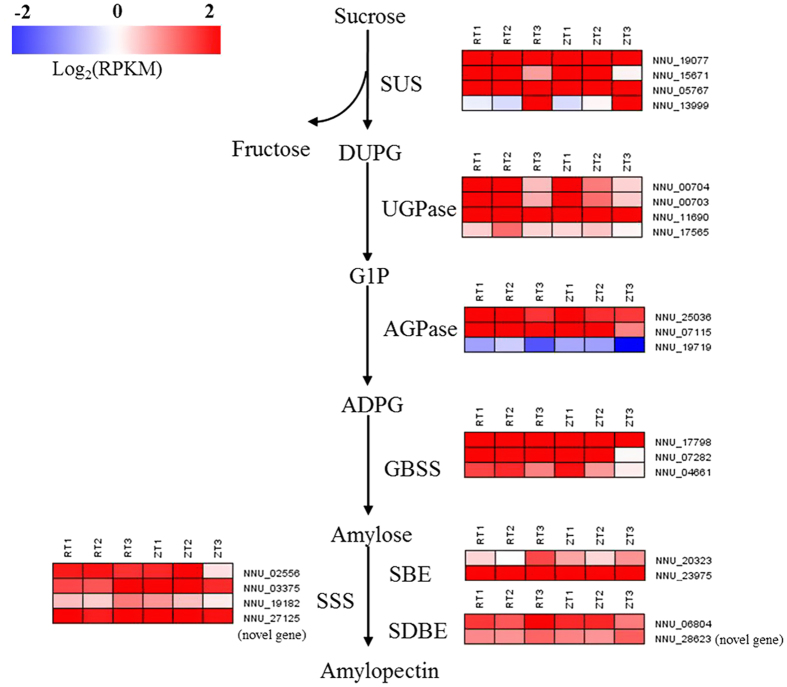
Expression patterns of the expressed genes assigned to starch biosynthesis in the six rhizome transcriptomes, RT1, RT2, RT3, ZT1, ZT2, and ZT3. The log-transformed expression values range from −2 to 2. Red and blue colors indicate up- and down- regulated transcripts, respectively.

The committed enzymes, sucrose synthase (SUS), DUP-glucose phrophosphorylase (UGPase), AGPase, GBSS, soluble starch synthase (SSS), starch branching enzyme (SBE), and starch debranching enzyme (SDBE), involved in starch biosynthesis were found in all six libraries (Fig. 4). Four transcripts encoding *SUS*, NNU_05767, NNU_13999, NNU_15761, and NNU_19077, were detected. NNU_05767 had the lowest expression at the T2 stage in ‘RL’, but was up-regulated during the development of rhizome in ‘ZO’. The expression of NNU_13999 started to increase at the T3 stage in ‘RL’, and was increased from the T2 stage in ‘ZO’. NNU_15761 had the highest expression at T2 stage in the two cultivars. The expression of NNU_19077 in ‘RL’ was the highest at T2 stage, but in ‘ZO’ it was down-regulated during the development of rhizome. Four homologs of *UGPase*, NNU_00703, NNU_00704, NNU_11690, and NNU_17565, showed completely opposite expression patterns between ‘RL’ and ‘ZO’. The expression of three transcript encoding *AGPase*, NNU_07115, NNU_19719, and NNU_25036, was unregulated in RT2 vs. RT1, then down-regulated in RT3 vs. RT2, while was down-regulated during the rhizome development in ‘ZO’. Three *GBSS* genes, NNU_04661, NNU_07282, and NNU_17798, had the lowest expression at T2 stage in ‘RL’, and were found to have a reduced expression level during the rhizome development in ‘ZO’. Most transcripts encoding *SSS*, *SBE*, and *SDBE* were up-regulated in ‘RL’, but down-regulated in ‘ZO’ as rhizome development proceeded (Fig. 4).

**Figure 5:**
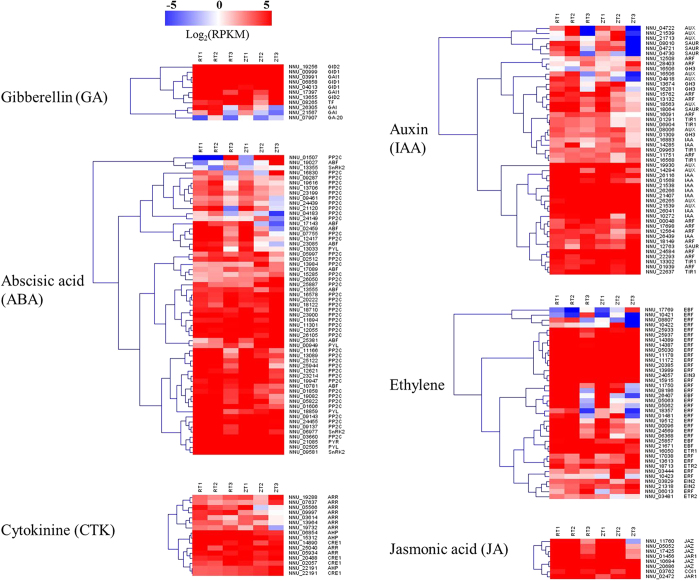
Heatmap of the expressed genes assigned to hormone signal transduction pathway in the six rhizome transcriptomes, RT1, RT2, RT3, ZT1, ZT2, and ZT3. The log-transformed expression values range from −5 to 5. Red and blue colors indicate up- and down- regulated transcripts, respectively.

A fairly large number of genes assigned to hormone signal transduction, hormone biosynthesis pathway, hormone responsive protein, and hormone transporter protein were identified (Fig. 5). These genes involving the GA, ABA, cytokinine (CTK), auxin, ethylene, Jasmonic acid (JA) pathways were divided into two clusters which showed completely opposite expression patterns. One cluster is enriched in genes that were down-regulated as rhizome development proceeded in both cultivars. The other cluster is enriched in genes that were up-regulated as rhizome development proceeded in both cultivars. The different homologs encoding one gene were distributed in the two clusters, respectively. The variations of the genes in ‘ZO’ were higher than in ‘RL’ in the two clusters.

**Figure 6:**
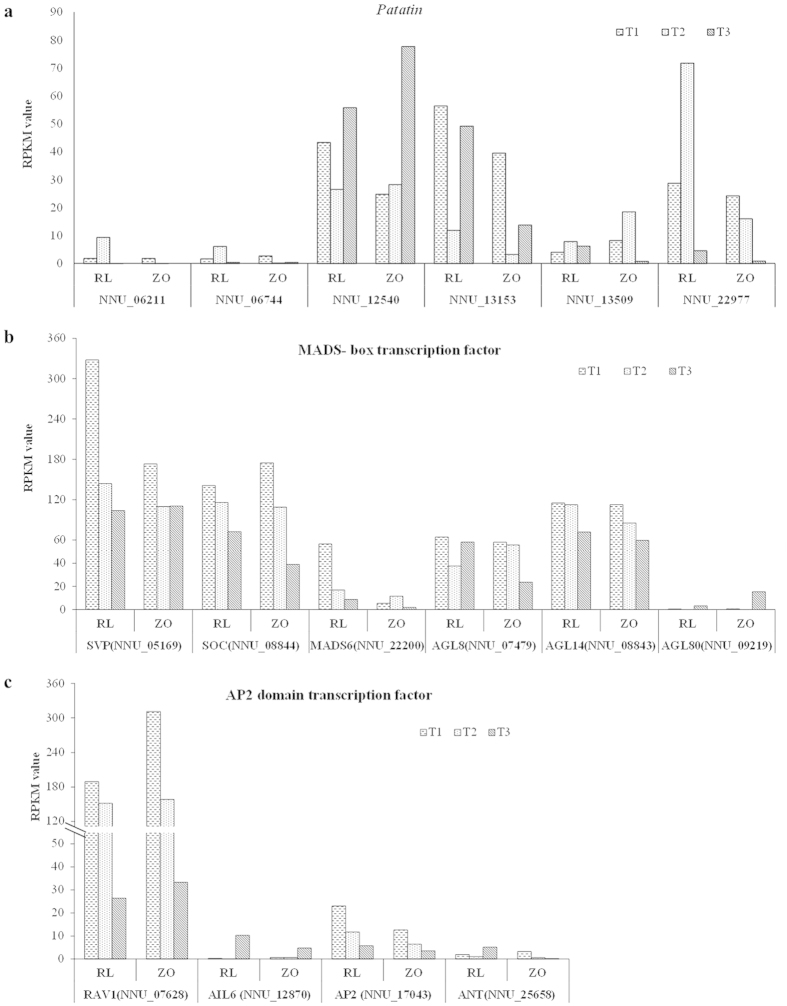
The expression level of six *Patatin* genes (a), six MADS-box transcription factors (b) and four AP2 domain transcription factors (c) at the three stages, T1, T2, and T3, between the two cultivars, ‘RL’ and ‘ZO’.

Six putative homologs of *Patatin*, which is usually believed to be important for storage organ development, were expressed in rhizome. The expression pattern of NNU_13153, NNU_13509, and NNU_22977 were similar in the two cultivars, while NNU_06211, NNU_06744, and NNU_12540 had different expression pattern (Fig. 6a). Moreover, six MADS transcription factors involved in photoperiod pathway, and four AP2 domain class transcription factors were identified in the six libraries. MADS-box protein *AGL80* (NNU_09219) and AP2-like transcription factor *AIL6* (NNU_12870) were up-regulated as rhizome development proceeded in both cultivars, and other transcription factors, *SVP* (NNU_05169), *SOC* (NNU_08844), *AIL14* (NNU_08843), *RAV1* (NNU_07628), *AP2* (NNU_17403) were down-regulated as rhizome development proceeded in both cultivars (Fig. 6b,c).

### Candidate genes involved in rhizome girth enlargement

**Figure 7:**
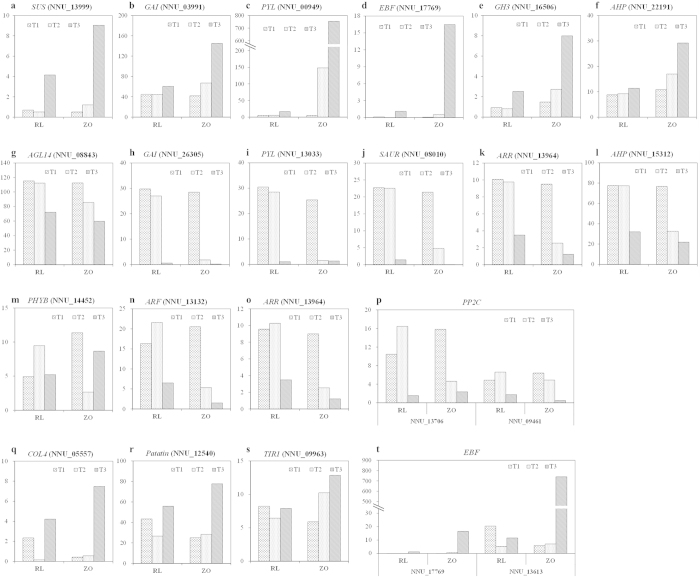
The RPKM value of 22 candidate genes controlling rhizome girth enlargement at the three stages, T1, T2, and T3, between the two cultivars, ‘RL’ and ‘ZO’.

Genes whose expression was highly correlated with rhizome enlargement index were potential candidates for involvement in rhizome girth enlargement. Based on our analysis, we suggested 12 candidate genes for rhizome girth enlargement. The expression of these genes at RT1 and RT2 had no significant difference to those at ZT1 because the rhizomes at these three stages are in stolon phase (Fig. 7a–i). The candidate genes were grouped into two major groups. The first group included six genes, *SUS* (NNU_13999), *GAI* (NNU_03991), *PYL* (NNU_00949), *EBF* (NNU_177769), *GH3* (NNU_16506), and *AHP* (NNU_22191), which positively correlated with rhizome enlargement index (Fig. 7a–f). The second group consisted of other six genes, *AGL14* (NNU_08843), *GAI* (NNU_26305), *PYL* (NNU_13033), *SAUR* (NNU_08010), *ARR* (NNU_13964), and *AHP* (NNU_15312), which negatively correlated with rhizome enlargement index (Fig. 7g–l). Except of *SUS* involving in starch biosynthesis and *AGL14* that is MADS-box transcription factor, others genes are assigned to hormone signal transduction pathway. *GAI*, *PYL*, and *EBF* involve in GA, ABA, and ethylene transduction pathway, respectively. *GH3* and *SAUR* involve in auxin transduction pathway, *AHP* and *ARR* involve in CTK transduction pathway.

In addition, genes that spiked between ZT1 and ZT2 and between RT2 and RT3 represented these genes controlling the switches in the process from stolon to rhizome, which also were the potential candidate genes for rhizome girth enlargement. Ten genes were found, and had no differential expression among RT2 and ZT1 (Fig. 7m–t). These genes were also classified into two groups: five genes, *PHYB* (NNU_14452), *ARF* (NNU_13132), *ARR* (NNU_13964), *PP2C* (NNU_13706 and NNU_09461), were expressed at a high level at the time point prior to the switch (Fig. 7m–p), and other genes, *COL4* (NNU_05557), *Patatin* (NNU_12540), *TIRI* (NNU_09963), *EBF* (NNU_17769 and NNU_13613), were expressed at a low level at the time point prior to the switch (Fig. 7q–t). *PHYB* and *COL4* are involved in photoperiod pathway. *ARF* and *TIRI* are involved in auxin transduction pathway. Functional analysis for these candidate genes may be useful for genetic engineering or marker assisted selection of the new lotus cultivars with enlarged rhizome.

### Verification of the gene expression through RT-qPCR

**Figure 8:**
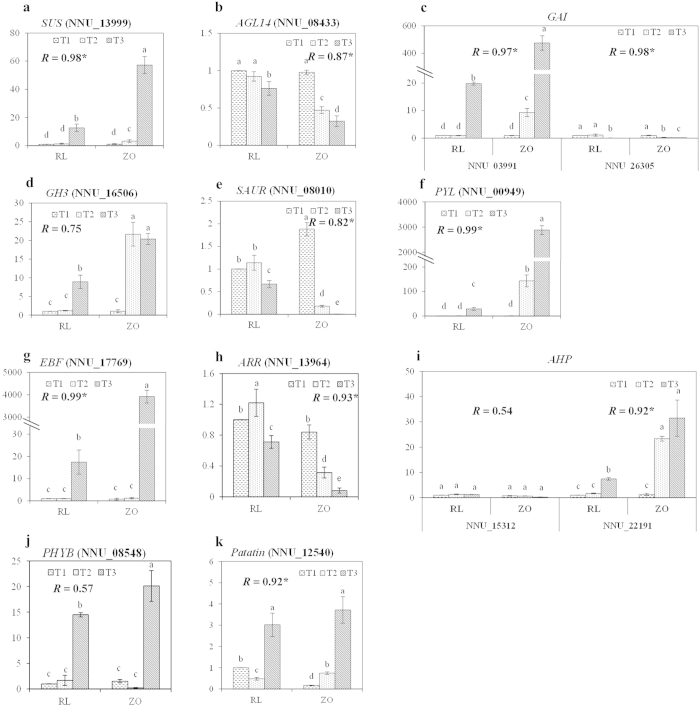
Real-time quantitative RT-qPCR confirmation of 13 candidate genes at the three stages, T1, T2, and T3, between the two cultivars, ‘RL’ and ‘ZO’. The left y-axis indicates relative gene expression levels determined by RT-qPCR. Relative gene expressions were normalized by comparison with the expression of lotus *β-actin* (NNU_24864), and analyzed using the 2^−ΔΔC^T Method. The expression values were adjusted by setting the expression of RT1 to be 1 for each gene. All RT-qPCRs for each gene used three biological replicates, with three technical replicates per experiment; the error bars indicate SE. Different lower case letter (**a**–**e**) indicates the significant difference among six treatments at *P* = 0.05. The correlation coefficient (*R*) for each gene between the RT-qPCR and RNA-Seq data is shown with the significant level (*P = 0.05).

Transcriptional regulation revealed by RNA-seq was confirmed in a biologically independent experiment using RT-qPCR. A total of 46 genes, including the 13 candidate genes, ten genes involved in photoperiod pathway, four genes involved in starch synthetic pathway, six involved in hormone transduction pathway, four *Patatin* genes, five transcription factor and four other genes, were chosen to design gene-specific primers (Table S1). The RT-qPCR results for all the genes were tested statistically, and each genes showed significantly different expression (*P* = 0.05, Fig. 8 and Fig. S3). Moreover, 28 gene showed significant correlations (*P* = 0.05) between the RT-qPCR data and the RNA-seq results, which indicated good reproducibility between transcript abundance assayed by RNA-seq and the expression profile revealed by RT-qPCR data (Fig. 8 and Fig. S3).

## Discussion

### Global gene transcription changes in lotus rhizome

The enlargement of lotus rhizome has been proved to be regulated by complex environmental and endogenous factors^10^. A global analysis of transcriptome could facilitate the identification of systemic gene expression and regulatory mechanisms, which has been used successfully for the analysis of transcriptomes of several species^23^,^24^,^25^,^26^,^27^. In this study, in order to screen for the genes controlling rhizome enlargement, we have sequenced and annotated the transcriptome of three rhizome developmental stages from two lotus cultivars. The two cultivars used in the study represented the distinct development of rhizome. The rhizome formation of the tropical lotus ‘RL’ was not merely a delayed process compared to the temperate counterpart ‘ZO’. Understanding the transcript profile of ‘RL’ could somehow inspire to uncover the gene controlling rhizome girth enlargement for ‘ZO’, which could provide a comprehensive understanding of gene expression during the rhizome formation in lotus.

In total, 185 and 173 million pair-end reads were generated from the cultivar ‘ZO’ and ‘RL’, respectively. An average of 88.5% of the data was mapped to the reference genome of *N. nucifera* (Table 1). This percentage of the mapped reads is the same as the flower transcriptome in lotus^22^. About 90% of identified genes in this study (24069) were predicted by Ming *et al*.^2^. These results suggested that the selected database is relatively complete. The rest of annotated genes (2714) in the study were not detected in the reference genes, which represented novel transcripts^2^,^22^. These gene sequences were annotated on the basis of searching four public databases. Analyses of the functional categories revealed that the genes encoding proteins were categorized as being involved in cellular process (24.2%), metabolic process (23.0%) (Fig. 2). The currently available expressed sequences would help to comprehensively understand the transcription profiles of lotus during rhizome development.

### Identification of DEGs in rhizome transcriptome

To identify corresponding genes that were associated with the development of rhizome for two cultivars, transcriptome-wide gene expression profiles were compared between the libraries of the three developmental stages. The number of DEGs identified in the comparisons of RT3 vs. RT1 and RT3 vs. RT2 was remarkably higher than that detected in the comparison of RT2 vs. RT1 (Fig. 3a). These variations were consistent with the morphological and physiological changes of rhizome for ‘RL’, which was little between T1 and T2, and rose from T2 to T3 (Fig. 1). Most of DEGs in RT2 vs. RT1 were involved in hydrolase activity, binding and vesicle membrane, which were different from the functions of DEGs in RT3 vs. RT1 and RT3 vs. RT2 (Fig. S1). The number of DEGs identified in the comparisons of ZT3 vs. ZT1 and ZT3 vs. ZT2 were higher than that detected between ZT1 and ZT2 (Fig. 3b), which corresponded to the change of rhizome enlargement index for ‘ZO’ that enlarged sharply at the T2 and T3 stages (Fig. 1). The total number of DEGs across the three stages was higher in ‘ZO’ compared to ‘RL’ at a particular stage, and the number of DEGs detected in same-stage comparisons between the two lotus cultivars was generally lower than that detected from same-cultivar comparisons at different stages (Fig. 3), which indicated that a number of genes involved in the rhizome development in ‘ZO’, and provided valuable resources for further elucidating the regulatory mechanisms that control rhizome enlargement.

The GO enrichment analysis revealed that carbohydrate metabolic and glucan metabolic processes were overrepresented terms for DEGs, which might contribute to starch accumulation in lotus rhizome (Fig. 3g and Fig. S1). KEGG analysis also revealed that dozens of genes were assigned to starch and sucrose metabolism, biosynthesis of secondary metabolites, hormone signal transduction, carbon metabolism, and so on (Fig. S2). These indicated that these processes play an important role in the rhizome development of lotus.

### DEGs involved in photoperiod pathway

Photoperiod is one of the most important environmental stimuli and regulates many physiological and developmental processes including flowering in several species^28^, and formation of storage organs in potato^14^. Photoperiodic control of flowering shared a number of common elements with that of tuberization regulation^11^. Day lengths are perceived by the leaves, and they synthesize a mobile signal ‘florigen’ or ‘tuberigen’ that is transported via the phloem to the vegetative shoot apex or the underground stolon tips to respectively induce floral or tuberization transition. The mobile *FT* protein was identified as the main component of the ‘florigen’ signal^29^,^30^, and controls the potato tuberization transition^15^. Activation of the *FT* gene by *CO* promotes flowering and inflorescence meristem identity for long-day (LD) flowering plant, and delays SD potato to tuberize in SD^12^,^31^. Flowering regulators, *PHYB*, *GI*, *RAP1* (related to *APETALA2 1*), *CO*, and *LKP2*, which positively regulated LD flowering, also played roles in tuberization^14^,^15^.

Lotus is a LD flowering plant, and the expression of these genes mediated flowering of lotus^22^, while the formation of enlarged rhizome strictly requires SD photoperiod^7^,^9^. In this study, during the growing season of lotus, the days got shorter, the girth of rhizomes got larger. The genes encoding *PHYB*, *CO*, *GI*, and *FT* were identified in lotus rhizome (Table 2). The expression of *PHYB*, which prevents tuberization, was decreased in ZT2 vs. ZT1 and ZT3 vs. ZT1, but enhanced ZT3 vs. ZT2. However, it had opposite expression pattern in ‘RL’ (Fig. 7m). The RT-qPCR confirmed the expression of *PHYB* in ‘RL’ and ‘ZO’ (Fig. S3). This indicated that *PHYB* played an important role in the process of switching from stolon to rhizome. Two homologs of *GI* had different expression pattern, and showed unclear relationship with rhizome enlargement index. The CO protein, a key component in the photoperiod pathway, is a member of an *Arabidopsis* gene family that comprises 16 other members (*COL1*–*COL16*). The different effect of *CO*, *COL1*, *COL2*, *COL3*, *COL5*, and *COL9* on flowering had been studied^32^,^33^. *CO* prevents tuberization under LD conditions in potato. The putative homologs of members of the *CO* gene family, including *COL1*–*COL5*, *COL7*, *COL9*–*COL10*, *COL13*–*COL15*, and *COL16*, were found with different expression patterns in the lotus transcriptome. Some genes, such as *COL2*, *COL3*, *COL10* and *COL13* were up-regulated during the rhizome development in two cultivars, whereas *COL1*, *COL14*, and *COL16* were down-regulated during the rhizome development in the two cultivars. Other homologs had opposite expression pattern in the two cultivars, and *COL4* was one of candidate genes for controlling the switch from stolon to rhizome (Fig. 7q). In the previous study, *COL1*, *COL3* and *COL13* promoted flowering, and *COL16* inhibited flowering in lotus^22^. It was speculated that these genes had different mechanisms that regulate flowering and rhizome formation in lotus. The two homologs of *FT*, NNU_20154 and NNU_26362, were down-regulated in the two cultivars during the rhizome formation, which might promote the switch from stolon to rhizome. The RT-qPCR validated the down-regulation of NNU_26362 as rhizome development proceeded in both cultivars (Fig. S3).

However, none of the photoperiodic genes was parallel with rhizome girth enlargement, and some of them were confirmed by RT-qPCR (Fig. 8 and Fig. S3). Therefore, the photoperiodic genes might play important role in the switch from stolon to rhizome. Cheng *et al*.^10^ found that the expression of *PHYB*, *GI* and *COL* were up-regulated at the stolon, initial swelling, and middle swelling stages, but down-regulated in later swelling stages. We tested the correlations of these genes between our study and their study, and found that they were not significantly correlated between the two studies (Table S3). Moreover, the expressions of other 11 commonly identified genes were compared in the two studies. The expressions for *AP2* and *Cycling Dof Factor* were positively correlated between the two studies, while those for *Lipoxygenase* and *BEL1-like HD transcription factor* were negatively correlated. The rest seven genes did not correlate well between the two studies (Table S3). The correlation analysis showed that these genes were not cross-validated by the two studies, which were due to the different cultivars and sampling seasons.

### The role of carbohydrate metabolism in the development of lotus rhizome

Carbohydrate metabolism is an essential process in plants that produces both energy sources and structural components of cells and cell walls. In our study, significant differences in carbohydrate metabolism, including glycolysis and metabolism of starch/sucrose, fructose/mannose, glucose/galactose, and pyruvate, were detected (Fig. 4 and Fig. S2). The starch biosynthesis is an important metabolism during the process of rhizome enlargement in lotus. The swelling of storage organ is in high coordination with the accumulation of starch in potato^14^, and the synthesis of starch in lotus was also positively related with the enlargement of rhizome (Fig. 1). The starting point for the starch synthetic pathway is sucrose, which is converted to fructose and DUPG by *SUS*^34^. The activity of *SUS* is correlated with the growth of potato tubers^35^. In this regard, four transcripts encoding *SUS*, NNU_05767, NNU_13999, NNU_15761, and NNU_19077, were detected in the study (Fig. 4). The expressions of NNU_13999 in two cultivars were increased in parallel with rhizome enlargement, which was presumed as one of the candidate gene for rhizome enlargement (Fig. 7a). The expression of NNU_13999 was confirmed by RT-qPCR (Fig. 8a). NNU_19077, which is similar to *SUS1*, highest expressed at T2 stage in ‘RL’, but down-regulated during the development of rhizome in ‘ZO’. As reported previously, *SUS1* is predominantly expressed in elongating tissues, including roots where the rapid formation of secondary wall takes place just after cell elongation^36^. According to high positive relationship between rhizome elongation and expression level, NNU_19077 was speculated to promote rhizome elongation in lotus.

AGPase, the committed step in the biosynthesis of starch, catalyzes the formation of ADP-glucose from G1P. Its isoforms present in chloroplasts/chromoplasts, amyloplast of all starch-synthesizing tissues^37^. Transgenic approaches in potato clearly found that the activity of *AGPase* was reduced by antisense repression which displayed a significantly reduced starch level, but these plants displayed normal tuber formation. The only change is that the number of tubers increased and the size of the individual tuber decreased^11^. In this study, we also found that two of *AGPase*, NNU_07115 and NNU_25036, were significantly down-regulated during the development of rhizome, which was opposite to rhizome enlargement index and starch accumulation (Figs 1 and 4). These results indicated that NNU_07115 and NNU_25036 suppressed rhizome enlargement of lotus, which consistent with the function of *AGPase* in potato.

The starch synthases catalyzes the transfer of the glucosyl moiety of the soluble precursor ADP-glucose to synthesize the insoluble glucan polymers amylose and amylopectin. *GBSS*, the first group of starch synthases genes, functions specifically to elongate amylase^37^. Inhibition of *GBSS* gene expression resulted in reduction of the production of amylase, but did not affect the expression level of other starch synthesizing enzymes^18^. The second group of starch synthases genes (designated *SSS*) are exclusively involved in amylopectin biosynthesis, and their distribution within the plastid between the stroma and starch granules varies between the species, tissue, and developmental stages^37^. In our study, both of *GBSS* and *SSS* showed opposite expression pattern in ‘RL’ and ‘ZO’. The expressions significantly increased in RT2 vs. RT1 and RT3 vs. RT1, but reduced in ZT2 vs. ZT1 and ZT3 vs. ZT1, although the starch content rose in two cultivars as the development proceed (Figs 1 and 4). The *GBSS* (NNU_07282) was also detected in Cheng *et al*.^10^, where it had similar expression tendency with our study (Table S3). These results indicated that these genes promoted starch synthesis in the early stage of starch accumulation in rhizome.

As discussed above, the expression of key genes involved in starch biosynthesis, except for *SUS* (NNU_13999), were not parallel with the variations of rhizome enlargement index in lotus. According to the principle of candidate gene chosen, only *SUS* (NNU_13999) was the candidate gene for rhizome girth enlargement. Therefore, these genes were required for starch accumulation in rhizome, but not required for the switch from stolon to rhizome in lotus. In potato, Fernie and Willmitzer 11 also reported that starch biosynthesis was not needed for tuberization.

### Complex regulation of plant hormone signal transduction in rhizome development

Plant hormones are involved in many different processes throughout the life of a plant, including growth, development and senescence. Several plant hormones such as GA, ABA, JA, ethylene have been reported to play an important roles in the formation of storage organs^11^. Our transcriptome analysis uncovered many genes that were involved in the phytohormone response to rhizome development. The specific regulation of rhizome formation might be governed by the endogenous levels of the hormones (Fig. 5).

GA is one of the most important plant hormones, and inhibits tuberization in potato. Xu *et al*.^38^ observed that high content of exogenous GA inhibited storage organ formation and promoted stolon elongation. Endogenous GA level was high during stolon elongation and decreased when stolon tips started to swell under inducing conditions in potato. The prevention of GA on tuberization is probably involved in the photoperiodic control of the formation of storage organ^14^. A dwarf mutant of potato plant that is able to tuberize in LD condition as well as in SD has been shown to have a partial block in GA biosynthetic pathway. Wild-type plants treated with ancymidol, an inhibitor of GA biosynthesis, will tuberize in LD, which is very similar to the formation of tubers on the antisense *PHYB* plants. These results suggest that GA is probably involved in the photoperiodic induction to regulate the formation of storage organ^39^,^40^. In this study, we obtained the families of the genes involved in GA biosynthesis and signal transduction pathway (Fig. 5). The expression of these genes was significantly regulated during the rhizome development. Two homologus of *GAI*, (NNU_21567) and (NNU_26305), were down-regulated as rhizome development proceeded, and could regulate rhizome girth enlargement. It had been reported that the high expression level of *GAI* promote early flowering in lotus^22^. Therefore, unlike potato, GA promoted flowering but inhibited rhizome girth enlargement in lotus. These findings indicated that rhizome swelling in lotus may be dependent upon a GA-mediated signal transduction process.

Unlike GA, exogenous ABA stimulated tuberization and reduced stolen length^38^. However, *Droopy*, an ABA deficient mutant of potato, is able to tuberize^41^. This indicated that ABA is not an essential component of the tuberization stimulus. The promotive effects of ABA on tuberization appear to be due to the antagonistic effects of ABA and GA^38^. In our study, numerous ABA-responsive transcripts, *ABF*, *PP2C*, *PYL*, *PYR*, and *snRK2* exhibited different expression levels in rhizome (Fig. 5). Only four genes, *ABF* (NNU_19027), *PP2C* (NNU_01507), *PYL* (NNU_00949), and *SnRK2* (NNU_13355) were significantly up-regulated, other genes were down-regulated (Fig. 5). These results suggested the positive-feedback of *ABF* (NNU_19027), *PP2C* (NNU_01507), *PYL* (NNU_00949), and *SnRK2* (NNU_13355) on rhizome girth enlargement in lotus. Other *PP2C* (NNU_13706 and NNU_09461) are expressed high at the time point prior to the switch in the two cultivars (Fig. 7p). Therefore these two genes were speculated as candidate genes that may control a switch from stolon to rhizome.

We found that a range of auxin, ethylene, cytokinin and JA -related genes were detected in our study, such as *AUX*/*IAA*, *ARF*, *GH3*, *SAUR*, *EIN*, *EBF*, *ERF*, *CRE*, *AHP*, *ARR*, *JAR1*, *COI1*, and *JAZ*. These genes were divided into two clusters which showed completely opposite expression patterns (Fig. 5). Seventeen out of 22 candidate genes for rhizome girth enlargement were assigned to hormone signal transduction pathway (Fig. 7). Interestingly, each two candidate genes from one hormone transduction pathway was plotted in the two opposite groups, which were confirmed by RT-qPCR. These candidate genes indicated that the hormones, especially GA and ABA, played a vital role in rhizome formation. Functional analysis for these candidate genes might be useful for genetic engineering or marker assisted selection of the new lotus cultivars with enlarged rhizome.

Overall, rhizome formation of lotus is a complex developmental process, which depends on the balanced expression of the genes within a complex network. The switch from stolon to rhizome in lotus might be controlled by the gene in photoperiod and hormone signal transduction pathway, and the rhizome girth enlargement (including the rapid accumulation of starch) might be controlled by the gene in starch biosynthesis and hormone signal transduction pathway. Further studies on the functions of these potential candidate genes might help to understand the rhizome formation in lotus.

## Conclusions

RNA-Seq analysis was used to monitor global transcriptional changes at the three developmental stages of rhizome in the lotus cultivars ‘ZO’ and ‘RL’, and it was enabled comprehensive analysis of differential transcriptional events that occurred during rhizome formation in lotus. In total, 26783 genes, including 2714 novel genes, were assembled, and 10299 DEGs were identified between the two cultivars at three stages. Hierarchical clustering plotted these DEGs into eight main clusters, which were significantly enriched in carbohydrate metabolism and glucan metabolic process. The DEGs involved in photoperiod, carbohydrate metabolism and hormone signal transduction pathways, which were reported previously as relevant to rhizome formation, were explored, and 22 candidate genes inducing rhizome girth enlargement were identified from these three pathways. Expression of 46 genes was confirmed by RT-qPCR, which had a high significant correlation with RNA-seq result. These results laid a solid foundation for future studies on molecular mechanisms underlying rhizome formation.

## Materials and Methods

### Plant materials and rhizome development measurements

Two cultivars, ‘ZO’ and ‘RL’ of *N. nucifera*, were used for transcriptome analysis. Whereas ‘ZO’ is a temperate lotus, of which rhizome can enlarge at the late growth stage, ‘RL’ is a tropical lotus with a thin rhizome. They have been conserved via rhizome at Wuhan Botanical Garden of the Chinese Academy of Sciences (N30°32′44.02′′, E114°24′52.18′′), Hubei Province, China for many years. The rhizomes of the two cultivars were planted in the trial plot of Wuhan Botanical Garden under the same cultivation conditions on 8^th^ April, 2013. Each cultivar was planted in three separate pools (3 m × 3 m), with three rhizomes per pool. Analysis of the developmental stage of rhizome identified three rhizome developmental stages for ‘ZO’, stolon (T1), middle swelling (T2), and later swelling (T3) stage for transcriptome sampling, which corresponded to the stage S1, S3, and S5 in Fig. 1a. The first internodes of main rhizome at three stages were sampled. Rhizomes for ‘RL’ were sampled at the corresponding day. The samples of the three stages were collected at 115, 154 and 207 days after planting (DAP), respectively. All samples were collected at 10 A.M, transferred immediately to liquid nitrogen, and stored subsequently at −80 °C until RNA extraction. The rhizomes of each stage were sampled from three comparable plants using as three biological replications. The samples of the stages T1, T2, and T3 for ‘RL’ and ‘ZO’ were used to construct six libraries, which were named as RT1, RT2, RT3, ZT1, ZT2, and ZT3, respectively.

Developmental stages of rhizome were characterized by the changes in rhizome enlargement index (= maximum rhizome diameter/ rhizome length) and starch content in the first internode. In order to calculate rhizome enlargement index, the length and maximum diameter of the internodes were measured after rhizomes were harvested. Starch content was determined by using the iodine binding colorimetry method^42^. Briefly, about 100 mg of drought sample was put into a 100-mL volumetric flask and then dissolved in 1 mL absolute ethyl alcohol and 9 mL NaOH (1 mol L^−1^). The dissolution was carried out by incubating at 85 °C for 10 min and then made up to 100 mL volume with water. 2.5 mL treatment dissolution was mixed with 1 mL acetic acid (1 mol L^−1^) and 1 mL iodine and then made up to 50 mL volume with water. The blend was immediately mixed and placed in the darkness for 20 min. Apparent amylase content was evaluated from the absorbance at 620 nm with ultraviolet-visible spectrophotometer (Beijing Puxi Instrument Company, Beijing, China). The recorded values were converted to percent of starch by reference to a standard curve prepared with amylose from potato and amylopectin from corn.

### RNA isolation, library construction, and sequencing

Total RNA was extracted using the Easyspin RNA reagent (RN38, Aidlab Biotechnology, Beijing, China) according to the manufacturer’s protocol, and treated with RNase-free DNase I (Takara, Dalian, China) to remove genomic DNA contamination. RNA integrity was evaluated with a 1.0% agarose gel stained with ethidium bromide (EB). Thereafter, the quality and quantity of RNA were assessed using a NanoPhotometer® spectrophotometer (IMPLEN, CA, USA) and an Agilent 2100 Bioanalyzer (Agilent Technologies, CA, USA). The RNA integrity number (RIN) was greater than 8.0 for all samples. For each developmental stage of the two cultivars, RNA samples from the three individuals were pooled together in equal amounts to generate one mixed sample. These four mixed RNA samples were subsequently used in cDNA library construction and Illumina sequencing which was completed by Beijing Novogene Bioinformatics Technology Co., Ltd.

A total amount of 3 μg RNA per sample was used to construct cDNA library. The library was generated using NEBNext^®^ Ultra™ RNA Library Prep Kit for Illumina® (NEB, USA) following manufacturer’s recommendations. Briefly, poly(A) mRNA was purified from total RNA using oligo(dT)-attached magnetic beads. The mRNA was then cleaved into small fragments by exposure to divalent cations under an elevated temperature in NEBNext first strand synthesis reaction buffer (5X). These fragments were used to synthesize first-strand cDNA using random hexamer primer and M-MuLV reverse transcriptase (RNase H^−^). Second-strand cDNA synthesis was subsequently performed using DNA polymerase I and RNase H. Remaining overhangs were converted into blunt ends via exonuclease/polymerase activities. After adenylation of 3′ ends of DNA fragments, NEBNext adaptor with hairpin loop structure were ligated to prepare for hybridization. In order to select cDNA fragments of preferentially 150~200 bp in length, the library fragments were purified with AMPure XP system (Beckman Coulter, Beverly, USA). Then 3 μl USER Enzyme (NEB, USA) was used with size-selected, adaptor-ligated cDNA at 37 °C for 15 min followed by 5 min at 95 °C before PCR. Then PCR was performed with phusion high-fidelity DNA polymerase, Universal PCR primers and index (X) primer. At last, PCR products were purified (AMPure XP system) and library quality was assessed on the Agilent Bioanalyzer 2100 system.

The clustering of the index-coded samples was performed on a cBot Cluster Generation System using TruSeq PE Cluster Kit v3-cBot-HS (Illumia) according to the manufacturer’s instructions. After cluster generation, the library preparations were sequenced on an Illumina Hiseq 2000 platform and 100 bp paired-end reads were generated. All raw-sequence reads data were deposited in NCBI Sequence Read Archive (SRA, http://www.ncbi.nlm.nih.gov/Traces/sra) with accession number SRA271278.

### Analysis of sequencing results: Mapping and differential expression

The raw reads were cleaned by removing adapter sequences, reads containing ploy-N, and low-quality sequences (Q < 20). Clean reads were aligned to the reference genome sequence^2^ (China Antique; 26,685 genes) using the program Tophat v2.0.9^43^. The tolerance parameters were the default settings, allowing mismatches of no more than two bases. Novel transcripts were identified from TopHat alignment results using Cufflinks v2.1.1 reference annotation based transcript (RABT) assembly method. For annotations, all novel genes were searched against the Nr database using BLASTx with 10^−5^ as E-value cut-off point and sequences with the highest similarities were retrieved. Using BLASTN, the identities between novel genes and the reference genes were detected with E-value < 10^−5^.

The DESeq package (ver. 2.1.0) was used to detect differentially expressed genes (DEGs) between the two samples. The false discovery rate (FDR) was used to determine the *P*-value threshold in multiple tests (Benjamini and Hochberg, 1995). An FDR ≤0.005 and the absolute value of the log_2_ (Fold change) with RPKM ≥ 1 were used as the thresholds to determine significant differences in gene expression in the study. The RPKM, reads per kb per million reads, eliminates the influence of different gene lengths and sequencing discrepancies on the quantification of gene expression to enable direct comparison of gene expression between samples, and is currently the most commonly used method to quantify gene expression^44^.

### Functional analysis of differentially expressed genes

Functional enrichment analyses including Gene Ontology (GO) and KEGG were performed to identify which DEGs were significantly enriched in GO terms or metabolic pathways. GO enrichment analysis of differentially expressed genes was implemented by the GOseq R package, in which gene length bias was corrected. GO terms with corrected *P* value less than 0.05 were considered significantly enriched by differential expressed genes. The GO annotations were functionally classified by WEGO software for gene function distributions. KOBAS software was used to test the statistical enrichment of differential expression genes in KEGG pathways. The pathways with an FDR value of ≤0.05 were defined as those with genes that display significant levels of differential expression.

### Quantitative real-time PCR validation of RNA-Seq data

Forty-six DEGs involved in rhizome development were chosen for validation using quantitative real-time PCR (RT-qPCR). Primers for RT-qPCR, which were designed with the Primer 3.0 software (http://biotools.umassmed.edu/bioapps/primer3_www.cgi), are listed in Table S1. RT-qPCR reactions were analyzed in the ABI StepOne^TM^ Plus Real-Time PCR System with the SYBR Green PCR Master Mix (Takara, Dalian, China), and amplified with 1 μl of cDNA template, 5 μL of 2 × SYBR Green Master Mix, and 0.2 μL of each primer (10 μmol/μL), to a final volume of 10 μL by adding water. The amplification program consisted of one cycle of 95 °C for 10 s, followed by 40 cycles of 95 °C for 15 s and 60 °C for 60 s. Fluorescent products were detected in the last step of each cycle. Melting curve analysis was performed at the end of 40 cycles to ensure proper amplification of target fragments. All RT-qPCR for each gene was performed in three biological replicates, with three technical repeats per experiment. Relative gene expression were normalized by comparison with the expression of lotus *β-actin* (NNU_24864), and analyzed using the 2^−ΔΔC^T Method^45^. The data were indicated as means ± SE (n = 9). Statistical analysis of RT-qPCR data was conducted using the ANOVA procedure of SAS 8.1 (SAS Institute, Cary, NC, USA).

## Additional Information

**How to cite this article**: Yang, M. *et al.* Transcriptomic Analysis of the Regulation of Rhizome Formation in Temperate and Tropical Lotus (*Nelumbo nucifera*). *Sci. Rep.* **5**, 13059; doi: 10.1038/srep13059 (2015).

## Supplementary Material

Supplementary Information

## Figures and Tables

**Table 1:** Summary statistics of clean reads in the rhizome transcriptomes of lotus.

	**RT1**	**RT2**	**RT3**	**ZT1**	**ZT2**	**ZT3**
Total reads	49292444	64923252	58358900	63523030	63701924	57705146
Base number (G)	4.92	6.5	5.84	6.35	6.37	5.77
High-quality reads(%)	97.37	97.13	97.25	97.34	97.22	97.3
Mapped reads	45674271	59897657	42906546	59166507	55147398	53435623
Exon (%)	84	85.9	77.6	83.9	85.1	75.3
Intron (%)	3.2	2.6	4.8	3.8	2.8	9.7
Intergenic (%)	12.7	11.5	17.6	12.2	12.1	15
Number of transcript	24832	24832	23871	24926	21937	22843
Number of novel transcript	2536	2524	2457	2581	2550	2427

**Table 2:** The genes involving the photoperiod pathway were related to rhizome formation identified previously.

**Gene name**	**Gene ID**	**RPKM value**					
		**RT1**	**RT2**	**RT3**	**ZT1**	**ZT2**	**ZT3**
PHYB	NNU_14452	4.92	9.46	5.22	11.37	2.65	8.64
*GI*	NNU_10096	19.29	7.17	19.11	36.84	32.79	56.89
*GI*	NNU_28780	5.96	1.81	19.48	19.32	16.31	8.36
*COL1*	NNU_08202	6.01	15.12	0.41	2.21	0.47	0.05
*COL2*	NNU_01086	161.43	65.55	147.69	112.54	25.26	351.50
*COL2*	NNU_17996	0.45	0.41	1.22	1.02	0.16	4.13
*COL3*	NNU_26112	6.72	10.56	21.27	1.90	3.33	16.63
*COL4*	NNU_05557	2.34	0.15	4.22	0.42	0.55	7.47
*COL5*	NNU_00495	202.91	185.92	61.01	80.37	140.92	187.22
*COL5*	NNU_09266	207.42	138.59	122.22	115.34	32.78	213.85
*COL5*	NNU_09327	61.53	20.91	125.17	22.32	6.12	70.12
*COL5*	NNU_13557	60.22	58.32	354.56	199.28	93.83	28.92
*COL7*	NNU_27522	2.66	1.08	3.96	2.35	0.47	8.45
*COL7*	NNU_17959	3.25	3.98	0.75	0.54	2.50	0.76
*COL9*	NNU_24588	4.53	6.20	1.43	5.19	2.19	2.16
*COL10*	NNU_06942	2.27	0.79	5.76	3.07	0.49	16.30
*COL13*	NNU_24804	13.76	3.51	42.75	19.14	4.74	125.19
*COL13*	NNU_02257	0.59	0.07	6.16	0.20	1.75	0.50
*COL14*	NNU_03991	28.29	35.66	23.76	37.94	14.79	29.39
*COL14*	NNU_03189	11.73	20.79	0.35	7.45	6.73	0.19
*COL14*	NNU_17798	1.58	2.19	1.63	1.66	1.09	3.80
*COL16*	NNU_01236	127.71	64.57	46.21	71.59	32.65	76.51
*COL16*	NNU_02963	5.62	2.42	0.09	2.19	0.90	0.05
*COL16*	NNU_12644	14.34	3.21	0.20	2.64	0.24	0.28
*FT*	NNU_20154	4.97	4.34	3.86	4.89	4.42	1.46
*FT*	NNU_26362	7.06	5.35	0.48	1.95	0.24	0.14
*FT*	NNU_15765	3.35	0.00	8.90	0.85	3.93	1.83

## References

[b1] Diao Y. *et al.* Nuclear DNA C-values in 12 species in nymphaeales. Caryologia 59, 25–30 (2006).

[b2] Ming R. *et al.* Genome of the long-living sacred lotus (*Nelumbo nucifera* Gaertn.). Genome Biology 14, R41 (2013). 23663246 10.1186/gb-2013-14-5-r41 PMC4053705

[b3] Shen-Miller J. Sacred lotus, the long-living fruits of China Antique. Seed Sci Res 12, 131–143 (2002).

[b4] Zhang X. Y., Chen L. Q. & Wang Q. C. New lotus flower cultivars in China (Beijing, 2011).

[b5] Wang Q. C. & Zhang X. Y. Colored illustration of lotus cultivars in China (Beijing, 2005).

[b6] Li X. Lotus of China (Beijing, 1987).

[b7] Masuda J., Ozaki Y. & Okubo H. Rhizome transition to storage organ is under phytochrome control in lotus (*Nelumbo nucifera*). Planta 226, 909–915 (2007). 17520280 10.1007/s00425-007-0536-9

[b8] Masuda J., Ozaki Y. & Okubo H. Regulation in rhizome transition to storage organ in lotus (*Nelumbo nucifera* Gaertn.) with exogenous gibberellin, gibberellin biosynthesis inhibitors or abscisic acid. J Jpn Soc Hortic Sci 81, 67–71 (2012).

[b9] Masuda J., Yoshimizu S., Ozaki Y. & Okubo H. Rhythmic response of rhizome growth to light-break in lotus (*Nelumbo nucifera*). J Fac Agr Kyushu U 52, 35–38 (2007).

[b10] Cheng L. B., Li S. Y., Yin J. J., Li L. J. & Chen X. H. Genome-wide analysis of differentially expressed genes relevant to rhizome formation in lotus root (*Nelumbo nucifera* Gaertn). Plos One 8, e67116 (2013). 23840598 10.1371/journal.pone.0067116 PMC3694149

[b11] Fernie A. R. & Willmitzer L. Molecular and biochemical triggers of potato tuber development. Plant Physiology 127, 1459–1465 (2001). 11743089 PMC1540178

[b12] Fischer L., Lipavska H., Hausman J. F. & Opatrny Z. Morphological and molecular characterization of a spontaneously tuberizing potato mutant: an insight into the regulatory mechanisms of tuber induction. BMC plant biology 8, 117 (2008). 19025587 10.1186/1471-2229-8-117 PMC2613151

[b13] Jackson S. D., Heyer A., Dietze J. & Prat S. Phytochrome B mediates the photoperiodic control of tuber formation in potato. The Plant J 9, 159–166 (1996).

[b14] Abelenda J. A., Navarro C. & Prat S. From the model to the crop: genes controlling tuber formation in potato. Curr Opin Biotechnol 22, 287–292 (2011). 21168321 10.1016/j.copbio.2010.11.013

[b15] Navarro C. *et al.* Control of flowering and storage organ formation in potato by *FLOWERING LOCUS T*. Nature 478, 119–U132 (2011). 21947007 10.1038/nature10431

[b16] Tjaden J., Mohlmann T., Kampfenkel K., Henrichs G. & Neuhaus H. E. Altered plastidic ATP/ADP-transporter activity influences potato (*Solanum tuberosum* L.) tuber morphology, yield and composition of tuber starch. Plant J 16, 531–540 (1998).

[b17] Geigenberger P., Merlo L., Reimholz R. & Stitt M. When growing potato-tubers are detached from their mother plant there is a rapid inhibition of starch synthesis, involving inhibition of adp-glucose pyrophosphorylase. Planta 193, 486–493 (1994).

[b18] Kuipers A. G. J., Jacobsen E. & Visser R. G. F. Formation and deposition of amylose in the potato-tuber starch granule are affected by the reduction of granule-bound starch synthase gene-expression. The Plant cell 6, 43–52 (1994). 12244219 10.1105/tpc.6.1.43 PMC160414

[b19] Carrera E., Bou J., Garcia-Martinez J. L. & Prat S. Changes in GA 20-oxidase gene expression strongly affect stem length, tuber induction and tuber yield of potato plants. Plant J 22, 247–256 (2000). 10849342 10.1046/j.1365-313x.2000.00736.x

[b20] Vreugdenhil D., Bindels P., Reinhoud P., Klocek J. & Hendriks T. Use of the growth retardant tetcyclacis for potato-tuber formation in-vitro. Plant Growth Regulation 14, 257–265 (1994).

[b21] Zhang X. Y. & Wang Q. C. Perliminary study of the eco-types of genetic resources of tropical lotus. Landscape Plants 22, 82–85 (2006).

[b22] Yang M., Zhu L., Xu L., Pan C. & Liu Y. Comparative transcriptomic analysis of the regulation of flowering in temperate and tropical lotus (*Nelumbo nucifera*) by RNA-Seq. Ann Appl Biol 165, 73–95 (2014).

[b23] Venturini L. *et al.* De novo transcriptome characterization of *Vitis vinifera* cv. Corvina unveils varietal diversity. BMC genomics 14, 41 (2013). 23331995 10.1186/1471-2164-14-41 PMC3556335

[b24] Xie F. *et al.* De novo sequencing and a comprehensive analysis of purple sweet potato (*Impomoea batatas* L.) transcriptome. Planta 236, 101–113 (2012). 22270559 10.1007/s00425-012-1591-4

[b25] Wang Z. *et al.* De novo characterization of the banana root transcriptome and analysis of gene expression under Fusarium oxysporum f. sp. Cubense tropical race 4 infection. BMC genomics 13, 650 (2012). 23170772 10.1186/1471-2164-13-650 PMC3534498

[b26] Wang X., Xu R., Wang R. & Liu A. Transcriptome analysis of Sacha Inchi (*Plukenetia volubilis* L.) seeds at two developmental stages. BMC genomics 13, 716 (2012). 23256450 10.1186/1471-2164-13-716 PMC3574040

[b27] Sweetman C., Wong D. C., Ford C. M. & Drew D. P. Transcriptome analysis at four developmental stages of grape berry (*Vitis vinifera* cv. Shiraz) provides insights into regulated and coordinated gene expression. BMC genomics 13, 691 (2012). 23227855 10.1186/1471-2164-13-691 PMC3545830

[b28] Amasino R. Seasonal and developmental timing of flowering. Plant J 61, 1001–1013 (2010). 20409274 10.1111/j.1365-313X.2010.04148.x

[b29] Taoka K. *et al.* 14-3-3 proteins act as intracellular receptors for rice Hd3a florigen. Nature 476, 332–U397 (2011). 21804566 10.1038/nature10272

[b30] Corbesier L. *et al.* FT protein Movement contributes to long-distance signaling in floral induction of *Arabidopsis*, Science 316, 1030–1033 (2007). 17446353 10.1126/science.1141752

[b31] Kardailsky I. *et al.* Activation tagging of the floral inducer *FT*. Science 286, 1962–1965 (1999). 10583961 10.1126/science.286.5446.1962

[b32] Datta S., Hettiarachchi G. H., Deng X. W. & Holm M. *Arabidopsis CONSTANS-LIKE3* is a positive regulator of red light signaling and root growth. Plant Cell 18, 70–84 (2006). 16339850 10.1105/tpc.105.038182 PMC1323485

[b33] Hassidim M., Harir Y., Yaki,r E., Kron I. & Green R. Over-expression of *CONSTANS-LIKE 5* can induce flowering in short-day grown *Arabidopsis*. Planta 230, 481–491 (2009). 19504268 10.1007/s00425-009-0958-7

[b34] Ogawa A., Audo F., Toyofuku K. & Kawashima C. Sucrose metabolism for the development of seminal root in maize seedlings. Plant Prod Sci 12, 9–16 (2009).

[b35] Zrenner R., Salanoubat M., Willmitzer L. & Sonnewald. Evidence of the crucial role of sucrose synthase for sink strength using transgenic potato plants (*Solanum tuberosum* L.). The Plant J 7, 97–107 (1995). 7894514 10.1046/j.1365-313x.1995.07010097.x

[b36] Hirose T., Scofield G. N. & Terao T. An expression analysis profile for the entire sucrose synthase gene family in rice. Plant Sci 174, 534–543 (2008).

[b37] Tetlow I. J., Morell M. K. & Emes M. J. Recent developments in understanding the regulation of starch metabolism in higher plants. Journal of experimental botany 55, 2131–2145 (2004). 15361536 10.1093/jxb/erh248

[b38] Xu X., van Lammeren A. A. M., Vermeer E. & Vreugdenhil D. The role of gibberellin, abscisic acid, and sucrose in the regulation of potato tuber formation *in vitro*. Plant Physiology 117, 575–584 (1998). 9625710 10.1104/pp.117.2.575 PMC34977

[b39] Jackson S. D. & Prat S. Control of tuberisation in potato by gibberellins and phytochrome B. Physiol Plantarum 98, 407–412 (1996).

[b40] Jackson S. D. Multiple signaling pathways control tuber induction in potato. Plant Physiol 119, 1–8 (1999). 9880339 10.1104/pp.119.1.1 PMC1539201

[b41] Quarrie S. A. Droopy: a wilty mutant of potato deficient in abscisic acid. Plant, Cell & Environment 5, 23–26 (1982).

[b42] Fang S. Q. & Liang S. W. Modern plant physiology experimental instruction (Beijing, 1999).

[b43] Trapnell C. *et al.* Differential gene and transcript expression analysis of RNA-seq experiments with TopHat and Cufflinks. Nature protocols 7, 562–578 (2012). 22383036 10.1038/nprot.2012.016 PMC3334321

[b44] Mortazavi A., Williams B. A., Mccue K., Schaeffer L. & Wold B. Mapping and quantifying mammalian transcriptomes by RNA-Seq. Nature methods 5, 621–628 (2008). 18516045 10.1038/nmeth.1226 PMC13303166

[b45] Livak K. J. & Schmittgen T. D. Analysis of relative gene expression data using real-time quantitative PCR and the 2^−ΔΔC^T method. Methods 25, 402–408 (2001). 11846609 10.1006/meth.2001.1262

