# Supplementary material for: Transcriptomic Analysis of the Regulation of Rhizome Formation in Temperate and Tropical Lotus (Nelumbo nucifera)
Source: Sci Rep. 2015 Aug 17;5:13059. doi: 10.1038/srep13059 (PMC4538393; doi:10.1038/srep13059)
Supplement: Supplementary Information [file srep13059-s1.pdf]

Supplementary Information

Transcriptomic Analysis of The Regulation of Rhizome Formation in Temperate and Tropical Lotus (*Nelumbonucifera*)

Mei Yang[1], Lingping Zhu[2,3], Cheng Pan[1], Liming Xu[1], Yanling Liu[2], Pingfang Yang[1]*

[1]Key Laboratory of Plant Germplasm Enhancement and Speciality Agriculture, Wuhan Botanical Garden, Chinese Academy of Sciences, Wuhan 430074, Hubei, China

[2]Key Laboratory of Aquatic Plant and Watershed Ecology, Wuhan Botanical Garden, Chinese Academy of Sciences, Wuhan 430074, Hubei, China

[3]University of Chinese Academy of Sciences, Beijing 100049, China

***Corresponding author:**

Key Laboratory of Plant Germplasm Enhancement and Speciality Agriculture, Wuhan Botanical Garden, Chinese Academy of Sciences, Wuhan 430074, Hubei, China

Pingfang Yang

E-mail: yangpf@wbgcas.cn

Fax: 86-27-87510956

List of Supplementary Materials

Table S1. Primers used to assay gene expression by RT-qPCR.

Table S2. The alignment of novel genes to the annotated genes of the reference lotus genome (‘China Antique’).

Table S3. Comparison of the expression of 14 common genes between our study and the previous study of Cheng et al. [10] who analyzed transcript profiling of lotus rhizome by sequence tag counting technology.

Figure S1. GO terms significantly enriched in DEGs in comparisons of RT1, RT2, RT3, ZT1, ZT2, and ZT3 libraries.

Figure S2. KEGG pathways significantly enriched in DEGs in comparisons of RT1, RT2, RT3, ZT1, ZT2, and ZT3 libraries.

Figure S3. Real-time quantitative RT-qPCR validation of 31 genes expressed at the three stages, T1, T2, and T3, between the two cultivars, ‘RL’ and ‘ZO’. The left y-axis indicates relative gene expression levels determined by RT-qPCR. Data were normalized against a lotus *β-actin* gene (NNU_24864) and analyzed using the $2^{-\Delta\Delta C}$T Method. The expression values were adjusted by setting the expression of RT1 to be 1 for each gene. All RT-qPCRs for each gene used three biological replicates, with three technical replicates per experiment; the error bars indicate SE. Different lower case letter (a, b, c, d, e) indicates the significant difference among six treatments at P = 0.05. The correlation coefficient (R) between the RT-qPCR and RNA-Seq data is shown for each gene.

Table S1. Primers used to assay gene expression by qRT-PCR

Gene name	Gene ID	Forward primer (5'→3')	Reverse primer (5'→3')
AGL14	NNU_08433	GACCCAGATGAAGCGGATAG	GAGAACCGAAAGCTCAAACG
AGL8	NNU_04430	TAGCACAAGAACGAGGATGG	CAGTTTGGACAATGAAGGGA
AGPase	NNU_11690	CCCTACAGAAACCGATAAGC	AAAGTCAATCCATTGCGAAC
AHP	NNU_15312	GAGACCCTCGCCAGTATGTG	TTCCGAATGGAGAACCCTAA
AHP	NNU_22191	TTATGACCTGCTGTTCCACG	GACCCACATCCATTCTTGCT
AP2	NNU_17043	CATACGACAGAGCAGCCATAA	GAGCCTCCCATCTTCCACAT
ARF	NNU_12564	GAAACAGTTCATCAGAAGGCAATA	TCAAGAGTCCGACCCACATC
ARR	NNU_13964	CTTATCCCTTCTGTCCGTTCC	CCAGTCCAGTTTCACGCTTT
AUX	NNU_14284	GTAAGAACATCCTGTCCGTGC	CTTTCCTAGAGCGTCAGAGAGC
BEL1	NNU_05289	GCCTCCGCTACATAGACCAA	ATGCCCGAAGGATAGAAACA
CML3	NNU_17861	GAGGCATTCAATGTGTTTGACC	CACCATCCCATCTCCATCC
COL	NNU_00495	ACCCACTCCGTCTCCATTTA	CGATGATACGCTCTGGCTAA
COL	NNU_08202	ACCTGGACATGGATTACGAT	TCTGGAGTGGGAGTTGGATA
COL	NNU_22255	ATTCCAGCACTGTCCAAACG	GACGAGCAGGCAACATAAGC
DOF	NNU_12021	AGCCTACCAGTGACCACGAT	GACCCTCCAGCAGATTACCA
EBF	NNU_17769	GAGCAGGATTTCAAGGGTAA	AAGTGATAGGAACGCAAGGA
ERF118	NNU_00508	ACGTTCTCTTGTCTTCATCTGG	TACAGCGTTTTCATCGTTCG
FT	NNU_20154	ACAGACGGTCTTCGCTCCAA	CCACCAGTTCCATTCTCCCT
FT	NNU_26362	GGTTGTTGGTCGTGTAATAGG	CTTGGACTTGGAGCATCAGG
GAI	NNU_03991	ACCACAACCTCCTCTTCAGCA	CAATAACGCCCGCATCTTTT
GAI	NNU_26305	TTGGGATACAAGGTTAGGTCG	CAGAAGCAAGATGGGAAAGC
GBSS	NNU_07282	CAAATGATTGGCACACTGC	TCATCTACTGGACCACGACC
GH3	NNU_16506	CACGACGGTGTAACTCCAAT	GGTAGACGCTGTTGAGGGAT
GI	NNU_20074	CACGGAGAACTGATACGCTAA	AGCCCTGAATCCTTGGTAAT
GID1	NNU_00999	GGCTGGAGGTAACGAAATCA	AGTTCCCGAAGAGTCTGGTG
KING1	NNU_02097	ATCACCCCTTCCAGGTTCC	ACGATGCTTCTCCTATGTTGC
Lipoxygenase	NNU_06221	GAAATGTCTTCCGTGCTCTA	CAAGTCATCGTTCGGGTAAT
MYB4	NNU_03430	ACCACCACAAACACCAAAAGG	CCGAAGACAATTCACTGGAGG
Patatin	NNU_06211	AGGCGTTACGCAGGAATGAT	ACGGCAGACCTTCCAGAGTT
Patatin	NNU_06744	CGCAGGAATGACGGTAAGTT	GCACGGGAGAAGACAAATGG
Patatin	NNU_12540	CCCGTTTTGATACCTTGTTACG	CCCAGAGCCAGAAGTCATAGC
Patatin	NNU_13153	GGAGGTATGCCGAGCAACGT	TCGCACGAACGGGAACTCAT
Patatin	NNU_22977	AGTATCCCCCAGAACTTCATCC	ACATTGCTGACTCCATTTCG
PHYA	NNU_09851	TGAGATGGTCCGTTTGATTG	TTCCCTGAAGTGCTAAGTGC
PHYB	NNU_08548	GTGGAATGCAGCAATGGA	ATCCACATATTTTCCTTTCC
PHYC	NNU_08548	CCCTCTTTCGGTCCTCCAGT	CCAAATCTATAACCAAACCCACAT
PP2C	NNU_01858	AATGCTACCTTGGCAAACAC	CCTCCACCACAGGAAGACAT

PP2C	NNU_03660	ACTTTGGAGCACGGAGGAGA	AACAGCAGAACCAAGAACCC
PYL	NNU_00949	GAGTCGCATGTCATGGGTTT	GTATCGGCGAACAAGCAAGTA
RAV1	NNU_07628	GCCGTCACCAACTTCAAGC	CTTTTACTCACATCGCCATTGC
SAUR	NNU_08010	GGTTCAAGCCGAGGAAGAGTA	GTGAAGGAGCGGACGAGATT
SOC1	NNU_08844	AGACCATAGAACGCTATCAACG	CCTTTCTTGCCCTGATGC
SSS	NNU_02556	TTTGAACCCTGTGGTCTAAACC	ATCTCTAAGCCCTCCAGTGC
SUS	NNU_13999	CCTTGTTGTTGTCGCTGGTT	ATTGCGTGCCTGGTTCATTT
SUS	NNU_19077	GACAATGATGCTGAATGACAGG	CCTCAAGAAGGTCCAGAAGC
WRKY	NNU_14734	TCATCAGAGCCCTCAACACC	GGTTCAAGAGCGAAATCACC

Table S2. The alignment of novel genes to the annotated genes of the reference lotus genome ('China Antique')

Novel gene ID	Length of novel gene	Reference gene ID	Length of reference gene	Identity	Length of identity	E-vaule	Score
NNU_26691	667	NNU_025217	4380	0.91	209	1.01E-75	153
NNU_26691	667	NNU_014307	1831	0.91	204	6.07E-73	148
NNU_26698	1716	NNU_024881	2068	0.90	252	3.36E-89	178
NNU_26698	1716	NNU_022209	1172	0.85	524	1.12E-143	276
NNU_26698	1716	NNU_017053	435	0.88	421	2.42E-140	270
NNU_26698	1716	NNU_002311	459	0.93	133	4.60E-48	104
NNU_26698	1716	NNU_010891	2188	0.90	120	1.68E-37	85
NNU_26698	1716	NNU_007740	4566	0.96	95	6.03E-37	84
NNU_26698	1716	NNU_017453	2338	0.89	86	6.16E-22	57
NNU_26700	1376	NNU_004154	2060	0.94	142	7.84E-55	116
NNU_26704	4388	NNU_022560	1183	0.94	171	2.48E-69	143
NNU_26704	4388	NNU_011088	711	0.93	171	5.36E-66	137
NNU_26704	4388	NNU_019155	3336	0.94	111	1.54E-41	93
NNU_26705	1396	NNU_020214	2091	0.95	387	4.01E-177	336
NNU_26705	1396	NNU_017533	434	0.96	263	2.02E-120	234
NNU_26705	1396	NNU_008852	1569	0.98	239	9.45E-114	222
NNU_26706	806	NNU_014645	1863	0.91	115	1.00E-36	83
NNU_26707	2694	NNU_025202	1029	0.80	939	0	370
NNU_26710	557	NNU_011240	504	0.96	52	7.05E-17	47
NNU_26712	6946	NNU_018306	3653	0.75	1263	9.55E-165	315
NNU_26712	6946	NNU_010865	6040	0.85	209	3.13E-50	109
NNU_26712	6946	NNU_009690	338	0.96	45	7.11E-12	40
NNU_26721	705	NNU_019843	1981	0.94	267	1.31E-114	223
NNU_26721	705	NNU_009408	1198	0.86	80	2.50E-17	48
NNU_26721	705	NNU_023186	813	0.81	89	2.52E-12	39
NNU_26730	1820	NNU_023881	1356	0.84	253	2.22E-61	128
NNU_26733	1494	NNU_004709	651	0.93	629	0	506
NNU_26734	7180	NNU_022370	377	0.96	185	4.01E-79	161
NNU_26734	7180	NNU_024189	525	0.94	175	8.73E-71	146
NNU_26734	7180	NNU_018179	2813	0.91	94	2.00E-27	68
NNU_26735	856	NNU_000871	762	0.96	76	2.33E-28	68
NNU_26736	1995	NNU_016415	1494	0.95	75	9.22E-26	64
NNU_26738	779	NNU_011722	3274	0.92	70	1.66E-19	52
NNU_26739	2112	NNU_023709	1481	0.82	568	1.40E-133	258
NNU_26739	2112	NNU_017396	315	0.92	293	3.09E-115	225

NNU_26740	3858	NNU_000345	1701	0.96	417	0	367
NNU_26740	3858	NNU_000651	4965	0.94	413	2.41E-178	339
NNU_26740	3858	NNU_009163	1290	0.85	583	1.13E-171	327
NNU_26740	3858	NNU_007096	354	0.91	110	6.36E-35	81
NNU_26740	3858	NNU_016129	809	0.87	161	2.25E-44	98
NNU_26740	3858	NNU_018062	1629	0.86	95	1.81E-20	55
NNU_26743	1027	NNU_024334	1371	0.94	367	1.41E-155	297
NNU_26745	1546	NNU_013295	676	0.95	466	0	400
NNU_26745	1546	NNU_002317	2794	0.89	192	5.24E-62	129
NNU_26745	1546	NNU_002327	2557	0.92	161	4.08E-58	122
NNU_26752	2517	NNU_000451	3033	0.97	384	0	352
NNU_26752	2517	NNU_000456	408	0.91	198	2.35E-72	148
NNU_26752	2517	NNU_002532	567	0.98	48	1.18E-15	46
NNU_26756	421	NNU_011078	346	0.99	116	2.99E-54	114
NNU_26756	421	NNU_021585	389	1.00	44	6.79E-16	45
NNU_26756	421	NNU_001080	1156	0.97	71	1.44E-27	66
NNU_26761	4458	NNU_016735	2186	0.83	1066	0	531
NNU_26761	4458	NNU_020393	968	0.91	149	5.56E-51	110
NNU_26764	2677	NNU_026278	1317	0.91	587	0	437
NNU_26764	2677	NNU_007154	468	0.88	382	3.88E-125	243
NNU_26766	2437	NNU_016851	1845	0.93	227	3.70E-90	180
NNU_26770	3288	NNU_011285	1338	0.95	86	4.21E-31	74
NNU_26770	3288	NNU_008023	1462	0.90	50	5.61E-10	36
NNU_26770	3288	NNU_003595	409	0.95	54	1.55E-15	46
NNU_26770	3288	NNU_008845	1446	0.98	59	1.19E-21	57
NNU_26770	3288	NNU_012119	1479	0.92	59	5.57E-15	45
NNU_26771	426	NNU_026652	1737	1.00	35	6.93E-11	36
NNU_26772	3243	NNU_008390	2300	0.77	442	3.98E-61	128
NNU_26773	5419	NNU_019707	3183	0.90	110	4.16E-33	78
NNU_26773	5419	NNU_000024	971	0.96	56	4.25E-18	51
NNU_26773	5419	NNU_016916	2220	0.88	76	5.50E-17	49
NNU_26773	5419	NNU_003416	3965	0.83	92	2.56E-15	46
NNU_26773	5419	NNU_007703	2002	0.95	43	7.17E-11	38
NNU_26773	5419	NNU_002963	1798	0.88	57	9.27E-10	36
NNU_26773	5419	NNU_000908	1704	1.00	28	7.22E-06	29
NNU_26774	1457	NNU_001632	567	0.89	121	8.55E-35	80
NNU_26774	1457	NNU_023355	2107	0.93	66	3.14E-19	52
NNU_26775	5400	NNU_022660	368	0.96	50	9.17E-15	45
NNU_26776	4216	NNU_004589	3138	0.88	181	2.43E-54	116
NNU_26778	2524	NNU_018465	4525	0.98	99	2.46E-42	94
NNU_26781	924	NNU_021623	743	0.93	464	0	372
NNU_26781	924	NNU_022542	673	0.90	514	0	354
NNU_26781	924	NNU_008323	2182	0.88	434	1.65E-144	277
NNU_26783	1743	NNU_025539	2646	0.87	61	2.28E-11	38

NNU_26783	1743	NNU_012068	1650	1.00	34	1.06E-09	35
NNU_26783	1743	NNU_020512	795	0.88	61	4.91E-13	41
NNU_26783	1743	NNU_005177	345	0.93	344	2.46E-140	270
NNU_26783	1743	NNU_019302	300	0.90	296	5.55E-107	210
NNU_26783	1743	NNU_026178	1896	0.97	71	6.22E-27	66
NNU_26783	1743	NNU_002174	2498	0.95	62	2.91E-20	54
NNU_26787	926	NNU_013541	2210	0.97	68	1.52E-25	63
NNU_26789	4464	NNU_016735	2186	0.83	1066	0	531
NNU_26792	2085	NNU_016753	1910	0.88	148	5.64E-43	95
NNU_26797	2921	NNU_022907	726	0.84	186	2.85E-42	94
NNU_26797	2921	NNU_022906	1575	0.89	127	7.99E-38	86
NNU_26802	1205	NNU_011801	1233	0.96	77	9.19E-29	69
NNU_26805	3592	NNU_008340	285	0.88	287	3.27E-92	184
NNU_26805	3592	NNU_008339	240	0.94	68	1.30E-21	57
NNU_26806	2632	NNU_016133	1128	0.95	455	0	383
NNU_26806	2632	NNU_010247	2872	0.84	235	2.51E-57	121
NNU_26807	2235	NNU_022139	1062	0.98	81	2.21E-32	76
NNU_26812	621	NNU_003523	5621	0.94	122	2.10E-47	102
NNU_26813	2744	NNU_005411	1487	0.91	122	4.48E-40	90
NNU_26813	2744	NNU_002170	2242	0.96	90	5.84E-34	79
NNU_26813	2744	NNU_026493	2025	0.93	43	1.68E-09	35
NNU_26814	2003	NNU_007397	555	0.86	170	1.95E-42	94
NNU_26816	1169	NNU_021438	3246	0.94	51	2.53E-14	43
NNU_26817	981	NNU_022480	864	0.90	162	9.29E-53	112
NNU_26819	627	NNU_018358	3427	0.91	252	1.19E-94	187
NNU_26820	2743	NNU_004918	321	0.96	52	1.29E-15	46
NNU_26822	1427	NNU_020911	1942	0.85	361	2.75E-99	196
NNU_26822	1427	NNU_010376	799	0.85	348	1.28E-97	193
NNU_26822	1427	NNU_007644	517	0.88	172	2.28E-50	108
NNU_26822	1427	NNU_018787	1068	0.93	83	1.82E-26	65
NNU_26825	5215	NNU_018731	1407	1.00	29	1.93E-06	30
NNU_26826	1134	NNU_012640	912	0.91	85	6.72E-25	62
NNU_26827	1995	NNU_024881	2068	0.92	237	3.02E-90	180
NNU_26827	1995	NNU_022209	1172	0.89	226	6.69E-72	147
NNU_26827	1995	NNU_017053	435	0.93	160	1.14E-59	125
NNU_26827	1995	NNU_002311	459	0.91	242	6.55E-87	174
NNU_26831	531	NNU_024923	912	0.92	121	6.47E-42	92
NNU_26832	661	NNU_005126	423	0.97	99	2.92E-41	91
NNU_26832	661	NNU_005124	423	0.97	99	2.92E-41	91
NNU_26832	661	NNU_005125	252	0.99	70	1.78E-28	68
NNU_26833	398	NNU_005125	252	0.99	98	2.85E-44	96
NNU_26833	398	NNU_005126	423	0.99	98	2.85E-44	96
NNU_26833	398	NNU_005124	423	0.99	98	2.85E-44	96
NNU_26833	398	NNU_005129	414	0.87	98	2.93E-24	60

NNU_26834	1632	NNU_018694	1109	0.94	98	9.59E-35	80
NNU_26840	844	NNU_001156	1757	1.00	33	1.82E-09	34
NNU_26841	502	NNU_011078	346	1.00	46	6.32E-17	47
NNU_26841	502	NNU_001080	1156	0.97	74	3.72E-29	69
NNU_26841	502	NNU_021585	389	1.00	47	1.76E-17	48
NNU_26842	294	NNU_002335	2572	0.93	60	9.93E-18	48
NNU_26842	294	NNU_011026	588	0.95	59	7.67E-19	50
NNU_26842	294	NNU_013103	320	0.94	103	7.46E-39	86
NNU_26844	637	NNU_015858	2227	0.97	484	0	440
NNU_26845	1940	NNU_024881	2068	0.93	185	6.50E-72	147
NNU_26845	1940	NNU_022209	1172	0.89	226	6.50E-72	147
NNU_26845	1940	NNU_002311	459	0.91	434	2.63E-170	324
NNU_26845	1940	NNU_017053	435	0.93	160	1.10E-59	125
NNU_26846	810	NNU_003240	4821	0.95	236	5.50E-104	204
NNU_26846	810	NNU_001754	1638	0.99	68	2.84E-27	66
NNU_26846	810	NNU_008327	3038	0.98	41	2.90E-12	39
NNU_26848	668	NNU_014840	1005	0.94	176	1.02E-70	144
NNU_26848	668	NNU_020839	1449	0.93	484	0	390
NNU_26850	546	NNU_019338	708	0.97	246	2.16E-116	226
NNU_26852	707	NNU_020268	1368	0.98	118	1.84E-53	113
NNU_26852	707	NNU_003501	2994	0.93	166	3.91E-65	134
NNU_26852	707	NNU_019657	2058	0.96	139	1.42E-59	124
NNU_26852	707	NNU_020709	1813	0.94	106	1.46E-39	88
NNU_26852	707	NNU_026156	834	0.96	76	1.91E-28	68
NNU_26852	707	NNU_019006	783	1.00	47	2.51E-17	48
NNU_26852	707	NNU_020607	11238	1.00	47	2.51E-17	48
NNU_26852	707	NNU_006247	12398	0.98	47	1.17E-15	45
NNU_26853	640	NNU_019657	2058	0.93	407	1.10E-164	313
NNU_26853	640	NNU_016153	3082	0.86	407	5.42E-123	238
NNU_26853	640	NNU_020268	1368	0.95	239	9.26E-106	207
NNU_26854	1671	NNU_019396	1534	0.87	1236	0	761
NNU_26854	1671	NNU_013094	3807	0.78	991	1.04E-173	330
NNU_26855	1409	NNU_026370	598	0.97	289	7.17E-135	260
NNU_26855	1409	NNU_014334	343	0.98	59	1.81E-21	56
NNU_26855	1409	NNU_024161	393	0.90	283	1.26E-97	193
NNU_26855	1409	NNU_025141	375	0.96	90	2.97E-34	79
NNU_26855	1409	NNU_024022	2305	0.84	136	2.99E-29	70
NNU_26855	1409	NNU_022546	842	0.94	67	1.81E-21	56
NNU_26855	1409	NNU_014577	3299	0.90	76	8.44E-20	53
NNU_26855	1409	NNU_012624	2304	0.95	57	1.41E-17	49
NNU_26858	3482	NNU_015963	2227	0.87	334	8.69E-103	203
NNU_26861	1199	NNU_010700	1074	0.91	571	0	422
NNU_26861	1199	NNU_025929	2439	0.92	178	2.42E-64	133
NNU_26861	1199	NNU_024209	816	0.73	427	1.17E-32	76

NNU_26862	3284	NNU_009987	2338	0.80	799	9.73E-162	309
NNU_26862	3284	NNU_015737	3181	0.90	124	3.23E-37	85
NNU_26867	3264	NNU_000105	8491	0.85	195	1.89E-49	107
NNU_26867	3264	NNU_018295	1659	0.92	96	5.41E-30	72
NNU_26867	3264	NNU_012954	4811	0.80	173	1.51E-25	64
NNU_26867	3264	NNU_023190	3293	0.97	35	9.31E-08	32
NNU_26871	2961	NNU_004709	651	0.92	624	0	481
NNU_26881	5627	NNU_006793	2234	0.95	141	5.40E-57	121
NNU_26881	5627	NNU_014030	1406	0.99	68	2.03E-26	66
NNU_26893	986	NNU_008140	2424	0.89	217	2.53E-73	149
NNU_26896	2129	NNU_022365	180	0.90	145	1.59E-48	105
NNU_26896	2129	NNU_016228	3982	0.96	65	7.67E-22	57
NNU_26896	2129	NNU_020181	1280	0.88	72	9.99E-16	46
NNU_26897	5917	NNU_023587	6443	0.88	189	1.58E-57	122
NNU_26897	5917	NNU_025181	4436	0.91	154	5.72E-52	112
NNU_26897	5917	NNU_021398	1920	0.87	80	7.77E-16	47
NNU_26898	1517	NNU_012659	2863	0.97	37	9.23E-10	35
NNU_26899	2582	NNU_005775	408	0.97	155	4.04E-70	144
NNU_26899	2582	NNU_019869	833	0.93	168	5.26E-64	133
NNU_26900	924	NNU_012719	3170	1.00	32	7.20E-09	33
NNU_26900	924	NNU_005228	950	0.88	45	3.35E-07	30
NNU_26916	620	NNU_005419	714	0.87	449	6.54E-147	281
NNU_26916	620	NNU_019894	381	0.86	359	1.15E-109	214
NNU_26916	620	NNU_010891	2188	0.89	287	1.95E-97	192
NNU_26916	620	NNU_015549	588	0.84	392	7.02E-97	191
NNU_26916	620	NNU_002891	429	0.86	262	5.58E-78	157
NNU_26917	2965	NNU_026179	931	0.88	168	6.18E-49	106
NNU_26917	2965	NNU_018192	1620	0.87	103	1.78E-24	62
NNU_26918	859	NNU_026229	783	0.92	118	1.78E-39	88
NNU_26921	985	NNU_010626	1381	0.98	56	1.63E-20	54
NNU_26921	985	NNU_005789	609	0.97	59	5.86E-20	53
NNU_26921	985	NNU_010248	324	0.94	46	1.28E-11	38
NNU_26925	1437	NNU_003770	1495	0.92	193	3.73E-68	140
NNU_26928	1352	NNU_015918	1215	0.93	216	4.42E-87	174
NNU_26928	1352	NNU_009765	1224	0.82	546	4.17E-127	246
NNU_26928	1352	NNU_008272	819	1.00	27	6.40E-06	28
NNU_26928	1352	NNU_013947	591	0.94	66	6.26E-21	55
NNU_26929	2755	NNU_022582	3065	0.94	208	1.52E-84	170
NNU_26931	480	NNU_009548	813	0.78	355	5.74E-52	110
NNU_26932	1418	NNU_026413	1746	0.93	185	4.73E-72	147
NNU_26933	714	NNU_000817	3097	0.92	73	3.25E-21	55
NNU_26935	1895	NNU_012606	428	0.93	75	4.07E-24	61
NNU_26940	1616	NNU_010626	1381	0.98	56	2.70E-20	54
NNU_26940	1616	NNU_005789	609	0.97	59	9.70E-20	53

NNU_26940	1616	NNU_012122	1214	0.92	47	2.74E-10	36
NNU_26940	1616	NNU_010248	324	0.94	46	2.11E-11	38
NNU_26941	3195	NNU_025539	2646	0.93	241	3.73E-96	191
NNU_26941	3195	NNU_012068	1650	0.95	91	8.80E-33	77
NNU_26941	3195	NNU_020512	795	0.93	398	9.40E-167	318
NNU_26941	3195	NNU_019302	300	0.96	243	1.31E-110	217
NNU_26941	3195	NNU_019303	339	0.83	369	6.38E-79	160
NNU_26941	3195	NNU_026066	2602	0.84	238	8.49E-58	122
NNU_26945	749	NNU_001509	1115	0.88	287	6.65E-93	184
NNU_26947	969	NNU_002374	1254	0.94	192	2.46E-78	158
NNU_26947	969	NNU_007328	1665	0.90	189	3.25E-62	129
NNU_26947	969	NNU_003770	1495	1.00	63	4.42E-26	64
NNU_26950	1009	NNU_000870	4044	0.90	273	8.98E-98	193
NNU_26950	1009	NNU_021663	474	0.95	62	1.67E-20	54
NNU_26950	1009	NNU_019675	440	0.96	88	2.74E-33	77
NNU_26950	1009	NNU_023065	228	0.95	77	3.56E-27	66
NNU_26951	1359	NNU_015918	1215	0.93	216	4.44E-87	174
NNU_26951	1359	NNU_009765	1224	0.82	546	4.19E-127	246
NNU_26951	1359	NNU_008272	819	1.00	27	6.43E-06	28
NNU_26951	1359	NNU_013947	591	0.94	66	6.30E-21	55
NNU_26953	1660	NNU_006263	3879	0.96	461	0	405
NNU_26953	1660	NNU_024464	2401	0.79	441	1.52E-82	166
NNU_26953	1660	NNU_018326	1371	0.99	147	7.18E-71	145
NNU_26953	1660	NNU_006264	417	0.95	128	5.71E-52	111
NNU_26956	1359	NNU_005587	2744	0.95	125	2.17E-50	108
NNU_26961	573	NNU_026673	1164	1.00	94	1.51E-43	95
NNU_26963	2467	NNU_012606	428	0.93	75	5.32E-24	61
NNU_26964	759	NNU_001141	1191	0.94	67	9.63E-22	56
NNU_26964	759	NNU_012765	3480	0.87	68	5.84E-14	42
NNU_26968	3653	NNU_012338	3819	0.88	300	7.15E-94	187
NNU_26971	2905	NNU_020133	1047	0.95	38	2.30E-08	33
NNU_26974	1819	NNU_021508	1830	0.82	548	1.21E-128	249
NNU_26974	1819	NNU_025936	1427	0.81	531	3.42E-119	232
NNU_26974	1819	NNU_021507	1008	0.81	504	7.45E-111	217
NNU_26975	1231	NNU_006627	1059	0.89	134	1.99E-40	90
NNU_26980	1736	NNU_024572	1353	0.85	87	1.75E-17	49
NNU_26982	1930	NNU_021561	1383	0.90	110	1.47E-33	78
NNU_26985	1015	NNU_022434	585	0.91	386	2.34E-148	284
NNU_26985	1015	NNU_021897	1865	0.94	243	4.17E-101	199
NNU_26985	1015	NNU_004188	1973	0.95	202	5.51E-85	170
NNU_26985	1015	NNU_012596	297	0.89	183	9.56E-58	121
NNU_26985	1015	NNU_005149	234	0.93	143	7.44E-54	114
NNU_26990	3012	NNU_025476	974	0.94	84	8.36E-28	68
NNU_26990	3012	NNU_019144	2645	0.99	84	1.38E-35	82

NNU_26990	3012	NNU_005379	690	1.00	55	3.92E-21	56
NNU_26990	3012	NNU_013070	1851	0.97	88	1.78E-34	80
NNU_26990	3012	NNU_024447	315	0.96	91	1.78E-34	80
NNU_26990	3012	NNU_024547	800	0.94	85	4.99E-30	72
NNU_26990	3012	NNU_000671	2394	0.84	113	1.09E-21	57
NNU_26990	3012	NNU_017273	368	0.84	106	1.41E-20	55
NNU_26990	3012	NNU_007381	1297	0.83	111	1.41E-20	55
NNU_26990	3012	NNU_015383	2314	0.86	95	5.06E-20	54
NNU_26996	862	NNU_020286	531	0.96	153	3.71E-66	136
NNU_26996	862	NNU_017947	1445	0.87	166	1.06E-46	101
NNU_26999	3011	NNU_024628	357	1.00	45	1.42E-15	46
NNU_27000	801	NNU_024628	357	0.93	72	7.87E-23	58
NNU_27007	3441	NNU_006202	1832	0.93	179	9.02E-68	140
NNU_27009	516	NNU_018146	2614	0.93	202	1.28E-78	158
NNU_27009	516	NNU_010316	2507	0.87	102	3.86E-24	60
NNU_27014	1054	NNU_020298	2205	0.81	761	3.06E-167	318
NNU_27014	1054	NNU_020300	2358	0.85	417	9.13E-118	229
NNU_27014	1054	NNU_017794	2289	0.76	762	2.61E-98	194
NNU_27015	1770	NNU_021106	861	0.94	114	1.72E-42	94
NNU_27015	1770	NNU_009800	471	0.94	108	8.00E-41	91
NNU_27015	1770	NNU_025703	372	0.92	112	1.34E-38	87
NNU_27015	1770	NNU_010852	721	0.89	71	8.28E-16	46
NNU_27016	3661	NNU_017816	3246	0.85	300	7.27E-84	169
NNU_27019	673	NNU_011077	1364	0.89	60	1.85E-13	41
NNU_27020	440	NNU_025027	627	0.97	109	5.27E-47	101
NNU_27020	440	NNU_016246	219	0.93	104	1.49E-37	84
NNU_27022	1076	NNU_021600	1011	0.84	572	1.48E-150	288
NNU_27023	1765	NNU_005601	2759	0.95	77	6.30E-27	66
NNU_27023	1765	NNU_005288	1245	0.94	151	3.61E-59	124
NNU_27023	1765	NNU_009451	3613	0.91	42	1.80E-07	31
NNU_27026	653	NNU_020665	1657	0.96	225	3.42E-100	197
NNU_27031	1014	NNU_017621	3087	0.94	256	1.15E-106	209
NNU_27031	1014	NNU_023451	972	0.91	195	2.02E-69	142
NNU_27031	1014	NNU_010557	1044	0.94	152	2.05E-59	124
NNU_27031	1014	NNU_012342	2118	0.95	79	2.77E-28	68
NNU_27031	1014	NNU_000085	237	0.93	79	6.00E-25	62
NNU_27034	1369	NNU_023139	567	0.83	487	3.29E-123	239
NNU_27034	1369	NNU_022594	291	0.90	286	7.33E-100	197
NNU_27035	3294	NNU_013518	1225	0.87	333	4.95E-100	198
NNU_27035	3294	NNU_007140	669	0.82	370	5.12E-75	153
NNU_27035	3294	NNU_000126	297	0.89	129	3.24E-37	85
NNU_27035	3294	NNU_013131	788	0.94	66	1.54E-20	55
NNU_27036	4470	NNU_022584	1295	0.86	935	0	529
NNU_27036	4470	NNU_022583	899	0.89	465	1.72E-160	307

NNU_27037	1686	NNU_011989	2150	0.96	149	1.23E-63	132
NNU_27037	1686	NNU_022585	543	0.80	179	3.62E-24	61
NNU_27037	1686	NNU_025702	1017	0.97	36	3.69E-09	34
NNU_27039	1739	NNU_004737	1642	0.92	92	2.86E-30	72
NNU_27041	883	NNU_012633	1817	1.00	47	3.15E-17	48
NNU_27041	883	NNU_024797	711	0.96	53	3.15E-17	48
NNU_27043	1269	NNU_018697	795	0.95	74	2.10E-25	63
NNU_27043	1269	NNU_022510	3503	0.86	118	3.48E-28	68
NNU_27043	1269	NNU_020083	1309	0.82	179	2.67E-34	79
NNU_27043	1269	NNU_016528	2420	0.90	77	2.11E-20	54
NNU_27043	1269	NNU_008204	708	1.00	38	4.60E-12	39
NNU_27044	805	NNU_015985	1728	0.94	150	5.82E-59	123
NNU_27046	1579	NNU_015118	603	0.95	140	5.39E-57	120
NNU_27052	715	NNU_014487	234	0.91	102	6.90E-33	76
NNU_27053	2091	NNU_007140	669	0.82	370	3.24E-75	153
NNU_27053	2091	NNU_013131	788	0.94	66	9.74E-21	55
NNU_27056	3630	NNU_015478	585	0.89	557	0	365
NNU_27056	3630	NNU_021223	573	0.89	453	1.81E-154	296
NNU_27059	1584	NNU_015614	2541	0.93	72	1.58E-22	58
NNU_27059	1584	NNU_023770	1012	0.85	117	2.62E-25	63
NNU_27059	1584	NNU_010891	2188	0.94	50	1.24E-13	42
NNU_27063	975	NNU_003605	1604	0.96	81	4.42E-31	73
NNU_27066	559	NNU_013613	2280	0.93	244	1.76E-97	192
NNU_27067	1892	NNU_003680	2768	0.92	63	1.90E-17	49
NNU_27068	948	NNU_021815	1485	0.96	450	0	403
NNU_27068	948	NNU_016236	972	0.96	453	0	399
NNU_27068	948	NNU_017752	1728	0.95	450	0	379
NNU_27068	948	NNU_000013	1668	0.86	406	2.26E-123	239
NNU_27068	948	NNU_014128	231	0.96	139	2.48E-58	122
NNU_27070	1077	NNU_026606	567	0.90	237	5.86E-85	170
NNU_27073	1978	NNU_004478	1815	0.90	1523	0	1077
NNU_27078	1100	NNU_020029	460	0.96	70	6.52E-25	62
NNU_27078	1100	NNU_005456	1281	0.93	56	6.61E-15	44
NNU_27079	3198	NNU_015963	2227	0.89	229	2.30E-78	159
NNU_27085	633	NNU_012103	1062	0.94	287	4.14E-124	240
NNU_27087	1070	NNU_020863	420	0.80	173	8.19E-24	60
NNU_27092	7108	NNU_012719	3170	1.00	29	2.64E-06	30
NNU_27093	1017	NNU_004213	1496	0.94	411	2.93E-172	327
NNU_27093	1017	NNU_002377	1446	0.93	80	1.67E-25	63
NNU_27096	797	NNU_023441	708	0.91	162	2.68E-57	120
NNU_27097	5736	NNU_007740	4566	0.89	256	3.17E-84	170
NNU_27097	5736	NNU_007469	1122	0.87	117	3.43E-29	71
NNU_27100	2294	NNU_002370	2306	0.82	1510	0	654
NNU_27102	5833	NNU_006122	2814	0.90	154	2.62E-50	109

NNU_27106	1649	NNU_011667	3946	0.86	258	4.26E-73	149
NNU_27108	1100	NNU_015025	3165	0.77	287	4.97E-36	82
NNU_27109	2203	NNU_002583	882	0.96	45	2.24E-12	40
NNU_27114	936	NNU_006782	481	0.84	205	8.92E-48	103
NNU_27120	495	NNU_022866	1822	0.85	205	9.92E-50	106
NNU_27120	495	NNU_016509	1670	0.86	181	1.28E-48	104
NNU_27121	761	NNU_011428	1274	0.92	119	1.21E-40	90
NNU_27121	761	NNU_021735	4484	0.96	70	4.46E-25	62
NNU_27123	964	NNU_015142	1007	0.83	171	9.32E-38	85
NNU_27123	964	NNU_016437	948	0.89	111	9.39E-33	76
NNU_27124	2523	NNU_024122	531	0.88	74	9.17E-17	48
NNU_27126	1082	NNU_019484	2201	0.92	126	6.23E-45	98
NNU_27129	546	NNU_018023	665	0.97	91	6.71E-37	83
NNU_27131	3705	NNU_023603	1334	0.82	999	0	454
NNU_27131	3705	NNU_023604	477	0.80	214	2.82E-38	87
NNU_27131	3705	NNU_013891	4066	0.75	163	2.27E-09	35
NNU_27132	2312	NNU_000105	8491	0.89	254	9.83E-86	172
NNU_27133	904	NNU_009850	1861	0.88	409	4.53E-140	269
NNU_27133	904	NNU_012922	3084	0.96	79	5.29E-30	71
NNU_27133	904	NNU_020809	481	1.00	32	7.04E-09	33
NNU_27143	858	NNU_017710	998	0.94	71	6.53E-24	60
NNU_27143	858	NNU_018356	1248	0.92	97	1.39E-30	72
NNU_27143	858	NNU_022987	1672	0.99	73	5.01E-30	71
NNU_27143	858	NNU_010778	2015	0.94	66	3.93E-21	55
NNU_27146	1523	NNU_026179	931	0.91	54	1.54E-12	40
NNU_27148	1414	NNU_001270	1211	0.88	368	9.44E-124	240
NNU_27148	1414	NNU_015258	2673	0.97	127	8.06E-55	116
NNU_27149	5828	NNU_011536	2652	0.94	131	9.42E-50	108
NNU_27149	5828	NNU_015379	2005	0.95	131	2.03E-51	111
NNU_27149	5828	NNU_011799	3165	0.90	104	2.08E-31	75
NNU_27149	5828	NNU_018374	327	0.94	67	7.60E-21	56
NNU_27149	5828	NNU_010689	12272	0.95	55	7.66E-16	47
NNU_27150	751	NNU_006463	2141	0.92	163	1.95E-58	122
NNU_27153	4951	NNU_018306	3653	0.76	1277	0	358
NNU_27161	3086	NNU_002668	6805	0.77	171	2.43E-13	42
NNU_27163	956	NNU_011763	1384	0.84	349	1.11E-91	182
NNU_27164	2788	NNU_003015	1310	0.86	88	2.18E-18	51
NNU_27164	2788	NNU_019907	1719	0.87	78	2.82E-17	49
NNU_27166	550	NNU_006520	716	0.84	91	6.96E-17	47
NNU_27172	1942	NNU_021126	758	0.82	120	3.25E-20	54
NNU_27172	1942	NNU_004544	413	0.92	87	1.93E-27	67
NNU_27172	1942	NNU_010144	437	0.89	73	5.44E-18	50
NNU_27172	1942	NNU_000752	673	0.85	393	1.33E-108	213
NNU_27172	1942	NNU_018454	774	0.83	427	1.04E-104	206

NNU_27172	1942	NNU_018474	305	0.93	57	9.10E-16	46
NNU_27172	1942	NNU_003286	414	0.88	112	1.15E-29	71
NNU_27172	1942	NNU_023930	743	0.91	112	2.46E-36	83
NNU_27172	1942	NNU_021045	297	0.90	209	1.40E-73	150
NNU_27172	1942	NNU_025156	411	0.94	85	1.15E-29	71
NNU_27172	1942	NNU_004540	309	0.87	240	2.34E-71	146
NNU_27172	1942	NNU_022375	423	0.84	260	6.55E-67	138
NNU_27172	1942	NNU_015186	287	0.86	227	5.10E-63	131
NNU_27172	1942	NNU_001444	744	0.87	108	6.94E-27	66
NNU_27172	1942	NNU_014619	558	0.92	170	6.60E-62	129
NNU_27172	1942	NNU_022647	499	0.93	134	8.66E-51	109
NNU_27172	1942	NNU_017240	300	0.86	222	1.43E-58	123
NNU_27172	1942	NNU_021363	282	0.86	209	1.43E-58	123
NNU_27172	1942	NNU_005319	467	0.92	145	5.18E-53	113
NNU_27172	1942	NNU_007191	336	0.89	172	4.00E-54	115
NNU_27172	1942	NNU_017939	301	0.91	151	1.86E-52	112
NNU_27172	1942	NNU_018147	318	0.91	151	8.66E-51	109
NNU_27172	1942	NNU_009701	318	0.91	151	8.66E-51	109
NNU_27172	1942	NNU_019355	1881	0.80	270	3.11E-50	108
NNU_27172	1942	NNU_018925	283	0.91	134	3.14E-45	99
NNU_27172	1942	NNU_021125	4062	0.82	117	3.25E-20	54
NNU_27172	1942	NNU_018119	422	0.90	135	4.06E-44	97
NNU_27172	1942	NNU_026333	552	0.93	111	4.09E-39	88
NNU_27172	1942	NNU_008359	216	0.86	170	5.25E-43	95
NNU_27172	1942	NNU_008167	1470	0.91	88	2.49E-26	65
NNU_27172	1942	NNU_017151	639	0.78	114	7.08E-12	39
NNU_27172	1942	NNU_018477	960	0.81	213	3.16E-40	90
NNU_27172	1942	NNU_021315	669	0.84	112	1.17E-19	53
NNU_27172	1942	NNU_024125	225	0.84	116	5.40E-23	59
NNU_27172	1942	NNU_001817	729	0.84	109	2.51E-21	56
NNU_27172	1942	NNU_002516	1158	0.98	45	4.23E-14	43
NNU_27172	1942	NNU_016433	405	0.90	89	3.23E-25	63
NNU_27172	1942	NNU_012332	615	0.86	115	3.23E-25	63
NNU_27172	1942	NNU_015825	482	0.88	93	4.17E-24	61
NNU_27172	1942	NNU_001683	2407	0.90	81	1.94E-22	58
NNU_27172	1942	NNU_025100	1056	0.96	49	1.18E-14	44
NNU_27172	1942	NNU_004982	345	0.90	57	1.97E-12	40
NNU_27172	1942	NNU_023030	500	0.86	69	1.97E-12	40
NNU_27172	1942	NNU_007518	391	1.00	29	7.14E-07	30
NNU_27174	1945	NNU_018179	2813	0.89	78	4.21E-19	52
NNU_27175	2181	NNU_001077	1788	0.83	369	1.54E-88	177
NNU_27175	2181	NNU_020981	903	0.84	381	1.52E-98	195
NNU_27175	2181	NNU_007252	468	0.84	368	7.07E-97	192
NNU_27175	2181	NNU_010260	795	0.84	369	1.53E-93	186

NNU_27175	2181	NNU_019069	828	0.80	275	1.26E-49	107
NNU_27177	3096	NNU_001849	1626	0.97	127	1.78E-54	116
NNU_27177	3096	NNU_018787	1068	0.93	111	1.82E-39	89
NNU_27178	902	NNU_000080	511	0.90	74	5.35E-20	53
NNU_27180	2495	NNU_009048	3900	0.99	68	8.94E-27	66
NNU_27184	1741	NNU_021526	216	0.89	191	5.91E-62	129
NNU_27185	654	NNU_025927	2007	1.00	166	2.11E-82	165
NNU_27188	495	NNU_017212	363	0.95	76	6.14E-27	65
NNU_27190	442	NNU_017657	1451	0.94	69	4.24E-23	58
NNU_27190	442	NNU_011752	551	1.00	27	2.02E-06	28
NNU_27190	442	NNU_026054	1862	1.00	27	2.02E-06	28
NNU_27195	3839	NNU_008981	3358	0.89	73	5.03E-16	47
NNU_27201	4566	NNU_012111	525	0.95	145	4.38E-57	121
NNU_27201	4566	NNU_000462	630	1.00	30	4.70E-07	31
NNU_27201	4566	NNU_006777	2034	0.97	97	2.69E-39	89
NNU_27203	845	NNU_025476	974	0.97	70	1.07E-26	65
NNU_27206	1631	NNU_011182	2134	0.93	400	3.68E-168	320
NNU_27206	1631	NNU_024239	579	0.82	151	1.25E-28	69
NNU_27206	1631	NNU_008507	435	0.91	340	8.38E-130	251
NNU_27206	1631	NNU_007556	2754	0.97	255	6.57E-121	235
NNU_27206	1631	NNU_026622	1329	0.95	253	4.01E-108	212
NNU_27206	1631	NNU_024963	321	0.94	224	4.10E-93	185
NNU_27206	1631	NNU_010365	486	0.89	112	4.46E-33	77
NNU_27208	190	NNU_025983	582	1.00	53	2.84E-21	54
NNU_27214	722	NNU_020278	657	0.85	163	1.15E-40	90
NNU_27216	1550	NNU_009292	385	0.95	98	1.51E-37	85
NNU_27217	724	NNU_021773	3093	0.97	186	1.40E-84	169
NNU_27220	2810	NNU_002856	1368	0.90	521	0	366
NNU_27220	2810	NNU_018359	1682	0.90	196	3.43E-66	137
NNU_27220	2810	NNU_002851	3157	0.82	199	2.74E-42	94
NNU_27223	1238	NNU_000747	1250	0.92	607	0	459
NNU_27223	1238	NNU_020290	459	0.81	254	2.55E-49	106
NNU_27224	2476	NNU_009426	450	0.96	444	0	385
NNU_27226	1035	NNU_019539	2996	0.81	918	0	385
NNU_27227	1954	NNU_022558	1152	0.93	247	1.05E-99	197
NNU_27227	1954	NNU_011153	4018	0.84	152	5.36E-33	77
NNU_27231	9995	NNU_011479	3229	0.93	60	3.65E-16	48
NNU_27231	9995	NNU_002617	4309	0.87	280	2.58E-82	167
NNU_27232	705	NNU_011428	1274	0.92	119	1.12E-40	90
NNU_27233	663	NNU_017671	3786	0.90	227	4.63E-79	159
NNU_27234	3572	NNU_008383	6243	0.94	355	2.31E-153	294
NNU_27234	3572	NNU_019602	4736	0.78	215	1.66E-25	64
NNU_27235	1363	NNU_011377	1803	0.93	262	4.33E-107	210
NNU_27235	1363	NNU_004510	2124	0.97	129	6.01E-56	118

NNU_27235	1363	NNU_015496	726	0.97	60	6.31E-21	55
NNU_27235	1363	NNU_018995	1149	0.95	57	1.37E-17	49
NNU_27235	1363	NNU_022414	2268	0.88	56	2.30E-10	36
NNU_27240	913	NNU_004289	2769	0.92	287	2.85E-112	219
NNU_27243	651	NNU_008198	1032	0.95	165	5.96E-68	139
NNU_27243	651	NNU_025639	261	0.93	165	1.29E-64	133
NNU_27247	4223	NNU_008383	6243	0.94	355	2.73E-153	294
NNU_27247	4223	NNU_008327	3038	0.85	173	5.34E-41	92
NNU_27247	4223	NNU_019602	4736	0.87	76	5.54E-16	47
NNU_27248	950	NNU_006615	2381	1.00	37	1.23E-11	38
NNU_27250	677	NNU_022715	2134	0.89	208	3.73E-65	134
NNU_27250	677	NNU_017018	1341	0.98	46	4.01E-15	44
NNU_27251	7093	NNU_003586	1512	0.86	374	3.77E-114	224
NNU_27251	7093	NNU_004770	2166	0.83	124	2.57E-21	57
NNU_27251	7093	NNU_025977	5169	0.88	66	1.56E-13	43
NNU_27254	9942	NNU_025173	3253	0.94	49	2.83E-12	41
NNU_27254	9942	NNU_006219	2926	0.92	48	4.74E-10	37
NNU_27265	932	NNU_001329	4100	0.92	72	4.28E-21	55
NNU_27265	932	NNU_025289	1250	0.87	137	1.17E-36	83
NNU_27266	800	NNU_002064	1194	0.94	62	6.12E-19	51
NNU_27267	1197	NNU_018001	2025	0.97	108	5.34E-46	100
NNU_27267	1197	NNU_014983	324	0.97	92	4.19E-37	84
NNU_27269	650	NNU_004910	3684	0.88	409	1.51E-133	257
NNU_27273	737	NNU_015858	2227	0.94	153	5.31E-59	123
NNU_27273	737	NNU_002744	630	1.00	57	7.22E-23	58
NNU_27273	737	NNU_001142	2459	0.98	59	2.60E-22	57
NNU_27277	2275	NNU_003163	4594	0.95	192	2.09E-82	166
NNU_27277	2275	NNU_013632	373	0.87	136	6.25E-33	77
NNU_27280	7866	NNU_001144	2131	0.83	1040	0	520
NNU_27283	5344	NNU_019568	10194	0.83	432	2.85E-109	215
NNU_27283	5344	NNU_005160	2298	0.83	87	3.26E-14	44
NNU_27287	690	NNU_010137	2418	0.95	128	1.08E-50	108
NNU_27287	690	NNU_016948	1683	0.93	125	1.09E-45	99
NNU_27289	1567	NNU_010340	819	0.98	94	9.14E-40	89
NNU_27289	1567	NNU_010849	2426	0.88	136	1.53E-37	85
NNU_27289	1567	NNU_006638	630	0.95	92	4.28E-33	77
NNU_27289	1567	NNU_006548	666	0.92	92	9.27E-30	71
NNU_27290	6124	NNU_006638	630	0.93	268	3.27E-109	215
NNU_27290	6124	NNU_006548	666	0.91	55	1.74E-12	41
NNU_27290	6124	NNU_004044	1086	0.96	68	4.77E-23	60
NNU_27290	6124	NNU_024894	240	0.94	226	3.34E-94	188
NNU_27290	6124	NNU_023862	1091	0.98	47	1.04E-14	45
NNU_27303	3110	NNU_017663	987	0.96	68	2.42E-23	60
NNU_27304	2210	NNU_022260	1308	0.87	679	0	419

NNU_27304	2210	NNU_022258	468	0.93	236	3.33E-95	189
NNU_27309	1481	NNU_012323	1206	0.89	348	5.96E-121	235
NNU_27310	2238	NNU_006966	2569	0.97	205	5.65E-93	185
NNU_27310	2238	NNU_013468	4060	0.96	204	3.40E-90	180
NNU_27311	1992	NNU_023277	1563	0.94	835	0	685
NNU_27312	10635	NNU_019220	1089	0.95	101	2.26E-38	88
NNU_27312	10635	NNU_012882	1458	0.95	90	2.94E-32	77
NNU_27315	13307	NNU_004610	768	0.89	208	7.59E-64	134
NNU_27317	1185	NNU_024689	2106	0.93	601	0	477
NNU_27317	1185	NNU_018847	633	0.94	347	7.62E-149	285
NNU_27318	2320	NNU_023841	1522	0.85	79	1.82E-13	42
NNU_27320	2294	NNU_003989	289	0.96	66	2.30E-22	58
NNU_27320	2294	NNU_018261	312	0.93	58	3.87E-15	45
NNU_27320	2294	NNU_005767	1998	0.97	130	2.83E-56	119
NNU_27320	2294	NNU_019839	2445	0.91	145	4.77E-49	106
NNU_27320	2294	NNU_018175	3180	0.95	58	6.44E-18	50
NNU_27322	3436	NNU_001495	1802	0.87	673	0	406
NNU_27323	1018	NNU_013518	1225	0.89	213	7.31E-69	141
NNU_27323	1018	NNU_000126	297	0.87	128	3.57E-32	75
NNU_27325	1298	NNU_010485	4363	0.91	344	1.85E-130	252
NNU_27328	1533	NNU_024119	1033	0.96	198	1.39E-87	175
NNU_27328	1533	NNU_000516	2817	0.97	115	8.81E-50	107
NNU_27328	1533	NNU_014098	2278	0.94	116	1.15E-43	96
NNU_27330	617	NNU_024335	1449	0.98	61	1.67E-23	59
NNU_27332	1791	NNU_022769	5052	0.92	982	0	759
NNU_27332	1791	NNU_001772	894	0.94	231	3.48E-94	187
NNU_27332	1791	NNU_012847	1012	0.96	74	2.30E-26	65
NNU_27332	1791	NNU_001771	510	0.91	300	1.58E-112	220
NNU_27332	1791	NNU_022770	453	0.88	335	9.48E-110	215
NNU_27332	1791	NNU_020289	252	0.92	185	3.61E-69	142
NNU_27332	1791	NNU_011441	626	0.90	132	1.74E-42	94
NNU_27339	3096	NNU_003989	289	0.95	65	1.12E-21	57
NNU_27339	3096	NNU_018261	312	0.95	54	1.89E-14	44
NNU_27339	3096	NNU_005767	1998	0.97	130	3.83E-56	119
NNU_27339	3096	NNU_019839	2445	0.91	145	6.46E-49	106
NNU_27339	3096	NNU_018175	3180	0.95	58	8.71E-18	50
NNU_27351	2455	NNU_008628	1389	0.93	125	3.98E-45	99
NNU_27353	7510	NNU_024993	2661	0.94	63	1.64E-18	52
NNU_27353	7510	NNU_019568	10194	0.85	79	5.94E-13	42
NNU_27357	3774	NNU_010118	2266	0.87	120	1.74E-30	73
NNU_27362	1627	NNU_005395	885	0.87	333	1.12E-103	204
NNU_27362	1627	NNU_006096	1803	1.00	31	4.61E-08	32
NNU_27362	1627	NNU_003637	3363	0.88	186	1.54E-57	121
NNU_27365	4431	NNU_019963	1331	0.86	186	1.99E-50	109

NNU_27365	4431	NNU_022532	1385	0.90	115	2.63E-34	80
NNU_27365	4431	NNU_014577	3299	0.96	51	2.09E-15	46
NNU_27368	1847	NNU_016081	717	0.98	63	3.97E-24	61
NNU_27368	1847	NNU_010339	2791	0.96	67	5.13E-23	59
NNU_27368	1847	NNU_007622	1965	0.90	205	2.22E-71	146
NNU_27368	1847	NNU_014290	865	0.84	96	1.86E-17	49
NNU_27371	1082	NNU_010025	1320	0.87	150	1.04E-42	94
NNU_27373	564	NNU_021207	1461	0.91	98	9.04E-31	72
NNU_27374	1152	NNU_013999	2203	0.87	203	1.81E-60	126
NNU_27375	534	NNU_009693	3296	0.89	72	5.21E-18	49
NNU_27376	936	NNU_022585	543	0.83	320	8.55E-78	157
NNU_27381	1634	NNU_013994	1843	0.91	1176	0	865
NNU_27381	1634	NNU_016182	1863	0.80	1142	0	456
NNU_27383	5858	NNU_005840	1809	0.88	85	9.88E-20	54
NNU_27384	1096	NNU_012277	1174	1.00	32	8.58E-09	33
NNU_27386	2388	NNU_021293	1422	0.89	70	8.67E-17	48
NNU_27386	2388	NNU_009835	1263	0.97	37	1.46E-09	35
NNU_27387	916	NNU_006722	2107	0.85	486	7.68E-138	265
NNU_27388	225	NNU_006722	2107	0.94	219	8.65E-92	181
NNU_27389	1288	NNU_026168	2643	0.89	279	4.14E-97	192
NNU_27393	276	NNU_004717	1147	0.97	86	1.95E-34	78
NNU_27393	276	NNU_004713	1384	0.94	79	3.29E-27	65
NNU_27393	276	NNU_022650	672	0.96	82	3.26E-32	74
NNU_27393	276	NNU_022653	534	0.94	86	4.22E-31	72
NNU_27393	276	NNU_004715	748	0.94	79	3.29E-27	65
NNU_27407	4172	NNU_003309	2535	0.97	33	4.29E-07	31
NNU_27408	1842	NNU_007864	4444	0.86	277	1.32E-78	159
NNU_27408	1842	NNU_021868	3754	0.83	280	2.89E-65	135
NNU_27412	413	NNU_026678	768	0.99	99	2.29E-45	98
NNU_27412	413	NNU_007379	1316	0.91	118	1.39E-37	84
NNU_27412	413	NNU_020429	375	0.94	99	5.00E-37	83
NNU_27412	413	NNU_007380	1324	0.89	118	3.01E-34	78
NNU_27414	916	NNU_011376	2115	0.84	484	4.69E-125	242
NNU_27416	1942	NNU_025238	2223	0.96	917	0	800
NNU_27416	1942	NNU_008440	3497	0.87	152	8.78E-41	91
NNU_27416	1942	NNU_005197	4773	0.89	126	6.84E-37	84
NNU_27416	1942	NNU_016509	1670	0.93	71	6.99E-22	57
NNU_27416	1942	NNU_013582	2698	0.90	77	4.20E-19	52
NNU_27416	1942	NNU_008260	1606	0.98	40	2.55E-11	38
NNU_27418	5246	NNU_001397	1838	0.78	312	3.07E-44	98
NNU_27420	2542	NNU_025197	607	0.92	134	8.85E-47	102
NNU_27422	7468	NNU_011368	1694	0.82	157	9.61E-31	74
NNU_27428	1834	NNU_000105	8491	0.78	360	1.76E-52	112
NNU_27430	3163	NNU_025238	2223	0.93	194	1.06E-76	156

NNU_27430	3163	NNU_008440	3497	0.87	152	1.44E-40	91
NNU_27430	3163	NNU_005197	4773	0.89	126	1.12E-36	84
NNU_27430	3163	NNU_016509	1670	0.93	71	1.14E-21	57
NNU_27430	3163	NNU_013582	2698	0.90	77	6.88E-19	52
NNU_27430	3163	NNU_008260	1606	0.98	40	4.17E-11	38
NNU_27433	3378	NNU_012817	870	0.97	159	1.47E-70	145
NNU_27434	1566	NNU_013059	7552	0.91	168	1.92E-56	119
NNU_27434	1566	NNU_017456	1068	0.93	84	1.55E-27	67
NNU_27439	1395	NNU_023195	2039	0.94	89	1.77E-31	74
NNU_27440	2235	NNU_010577	855	0.97	380	0	343
NNU_27440	2235	NNU_005639	1620	0.88	466	2.41E-151	290
NNU_27440	2235	NNU_012342	2118	0.93	338	8.79E-141	271
NNU_27440	2235	NNU_000086	444	0.91	185	9.78E-66	136
NNU_27440	2235	NNU_006711	657	0.94	162	4.55E-64	133
NNU_27442	1075	NNU_010761	1222	0.92	216	4.55E-81	163
NNU_27443	2147	NNU_018605	3015	0.94	374	1.06E-159	305
NNU_27443	2147	NNU_020482	2307	0.90	122	1.63E-38	87
NNU_27443	2147	NNU_020483	261	0.83	157	4.56E-34	79
NNU_27444	2289	NNU_012342	2118	0.94	491	0	398
NNU_27444	2289	NNU_010577	855	0.96	354	2.42E-166	317
NNU_27444	2289	NNU_000086	444	0.86	338	1.61E-93	186
NNU_27444	2289	NNU_005639	1620	0.93	174	1.00E-65	136
NNU_27444	2289	NNU_006711	657	0.93	136	2.84E-51	110
NNU_27445	7831	NNU_006709	3256	0.86	319	4.31E-89	179
NNU_27445	7831	NNU_006009	2228	0.80	386	1.24E-64	135
NNU_27445	7831	NNU_019670	1557	0.93	94	1.01E-30	74
NNU_27445	7831	NNU_024899	3722	0.83	158	4.69E-29	71
NNU_27448	486	NNU_015330	2378	0.98	45	1.02E-14	43
NNU_27448	486	NNU_017715	2110	0.98	62	3.63E-24	60
NNU_27448	486	NNU_019106	1804	0.90	62	7.90E-16	45
NNU_27453	837	NNU_018987	588	1.00	32	6.50E-09	33
NNU_27453	837	NNU_004213	1496	1.00	42	1.80E-14	43
NNU_27453	837	NNU_010299	471	0.90	182	1.68E-59	124
NNU_27453	837	NNU_020895	171	0.90	96	8.18E-28	67
NNU_27454	3369	NNU_022071	1878	0.87	77	3.41E-17	49
NNU_27454	3369	NNU_007766	4299	0.85	76	2.65E-13	42
NNU_27460	1155	NNU_009590	1260	0.85	320	1.74E-90	180
NNU_27464	4596	NNU_009142	2628	0.81	277	1.22E-57	122
NNU_27464	4596	NNU_004745	492	0.87	96	4.63E-22	58
NNU_27467	4132	NNU_019087	300	1.00	62	6.91E-25	63
NNU_27469	2757	NNU_002388	2638	0.77	2281	0	659
NNU_27471	916	NNU_025929	2439	0.95	146	1.43E-60	126
NNU_27471	916	NNU_023677	1617	0.94	92	3.21E-32	75
NNU_27474	4314	NNU_002927	654	0.94	168	4.07E-67	139

NNU_27477	754	NNU_023138	561	0.93	272	2.34E-112	219
NNU_27477	754	NNU_008554	810	0.86	262	1.14E-75	153
NNU_27478	333	NNU_023138	561	0.96	142	1.78E-60	125
NNU_27484	6914	NNU_005539	3351	0.83	218	2.42E-46	102
NNU_27485	1864	NNU_011205	1746	0.82	1079	0	502
NNU_27486	617	NNU_026208	2283	0.93	579	0	451
NNU_27487	2156	NNU_019154	4083	0.92	237	2.53E-91	182
NNU_27487	2156	NNU_013237	4619	0.98	45	4.71E-14	43
NNU_27487	2156	NNU_018795	666	0.97	37	1.32E-09	35
NNU_27487	2156	NNU_021212	4133	0.96	165	9.36E-71	145
NNU_27487	2156	NNU_002886	752	0.83	139	2.77E-26	65
NNU_27488	1280	NNU_021678	2946	0.92	290	8.66E-114	222
NNU_27488	1280	NNU_013416	1143	0.86	359	1.46E-106	209
NNU_27489	2295	NNU_020911	1942	0.93	388	1.47E-158	303
NNU_27489	2295	NNU_010376	799	0.92	372	1.15E-149	287
NNU_27489	2295	NNU_007644	517	0.89	276	1.25E-89	179
NNU_27489	2295	NNU_026128	522	0.95	39	5.05E-09	34
NNU_27493	1250	NNU_023586	4593	1.00	28	1.64E-06	29
NNU_27493	1250	NNU_005451	2106	0.97	31	1.64E-06	29
NNU_27497	3036	NNU_000451	3033	0.92	576	0	451
NNU_27497	3036	NNU_011203	3321	0.85	180	4.93E-45	99
NNU_27497	3036	NNU_000456	408	0.90	134	1.07E-41	93
NNU_27497	3036	NNU_010891	2188	0.91	120	6.42E-39	88
NNU_27497	3036	NNU_023770	1012	0.96	49	1.85E-14	44
NNU_27498	984	NNU_006803	487	0.95	104	5.69E-40	89
NNU_27503	698	NNU_008482	462	0.88	154	6.64E-43	94
NNU_27506	688	NNU_000105	8491	0.91	54	6.82E-13	40
NNU_27506	688	NNU_023190	3293	0.93	44	1.14E-10	36
NNU_27506	688	NNU_022848	3240	1.00	30	6.87E-08	31
NNU_27507	3574	NNU_006672	1925	0.98	43	1.01E-12	41
NNU_27507	3574	NNU_022664	1232	0.84	66	7.89E-09	34
NNU_27507	3574	NNU_001234	2239	1.00	29	1.32E-06	30
NNU_27512	1676	NNU_002750	2172	0.90	116	7.62E-36	82
NNU_27512	1676	NNU_001299	1423	0.88	65	1.31E-13	42
NNU_27512	1676	NNU_020594	1389	0.95	38	1.32E-08	33
NNU_27513	3542	NNU_010002	666	0.87	567	3.70E-176	335
NNU_27513	3542	NNU_010003	489	0.95	95	5.83E-35	81
NNU_27513	3542	NNU_010001	342	0.95	65	1.28E-21	57
NNU_27513	3542	NNU_005031	2346	0.98	43	1.00E-12	41
NNU_27513	3542	NNU_024617	2883	1.00	28	4.70E-06	29
NNU_27519	5203	NNU_011285	1338	0.90	343	7.56E-125	243
NNU_27519	5203	NNU_012814	1566	0.95	97	6.64E-36	83
NNU_27519	5203	NNU_025653	2265	0.87	153	1.10E-43	97
NNU_27519	5203	NNU_002907	1040	0.92	121	5.10E-42	94

NNU_27520	530	NNU_019886	225	0.93	140	1.77E-52	111
NNU_27520	530	NNU_011748	1422	1.00	57	5.14E-23	58
NNU_27521	2292	NNU_013333	4081	0.92	72	1.07E-20	55
NNU_27522	1923	NNU_024477	2439	0.93	134	1.43E-48	105
NNU_27523	1767	NNU_017224	1056	0.82	254	2.83E-50	108
NNU_27532	358	NNU_010937	984	0.97	33	3.46E-08	31
NNU_27535	992	NNU_004374	1620	0.89	197	9.27E-63	130
NNU_27541	4078	NNU_024718	1382	0.82	127	1.14E-22	59
NNU_27545	1769	NNU_005103	862	0.83	216	1.02E-49	107
NNU_27547	1756	NNU_014624	518	0.90	126	3.69E-39	88
NNU_27548	1046	NNU_024165	885	0.89	86	3.72E-22	57
NNU_27553	1700	NNU_013999	2203	0.88	134	1.28E-38	87
NNU_27554	995	NNU_014047	2120	0.86	445	1.40E-135	261
NNU_27555	1941	NNU_003942	3947	0.89	192	1.83E-62	130
NNU_27558	992	NNU_009667	4617	0.79	323	4.34E-56	118
NNU_27558	992	NNU_015379	2005	0.85	119	3.50E-27	66
NNU_27562	5812	NNU_016437	948	0.93	302	8.45E-125	243
NNU_27562	5812	NNU_025067	991	0.93	293	8.51E-120	234
NNU_27562	5812	NNU_015142	1007	0.86	92	9.81E-20	54
NNU_27562	5812	NNU_023274	1008	0.89	314	8.69E-105	207
NNU_27566	3548	NNU_023248	3440	0.89	148	2.07E-44	98
NNU_27567	3097	NNU_025710	3204	0.97	31	4.11E-06	29
NNU_27569	1289	NNU_019602	4736	0.88	213	4.33E-67	138
NNU_27569	1289	NNU_000710	1088	0.87	117	2.74E-29	70
NNU_27570	3015	NNU_012316	729	0.93	269	3.48E-106	209
NNU_27570	3015	NNU_003745	1275	0.91	210	2.80E-77	157
NNU_27571	4485	NNU_008372	282	0.90	281	6.75E-100	198
NNU_27575	2914	NNU_000235	907	0.91	586	0	436
NNU_27575	2914	NNU_009639	2438	0.93	57	1.37E-15	46
NNU_27575	2914	NNU_016587	4607	1.00	28	3.87E-06	29
NNU_27576	1972	NNU_019833	531	0.86	118	1.96E-27	67
NNU_27580	768	NNU_021656	489	0.96	50	1.27E-15	45
NNU_27580	768	NNU_014832	633	1.00	32	5.95E-09	33
NNU_27581	483	NNU_018748	466	0.93	252	4.16E-103	202
NNU_27582	588	NNU_007815	438	0.97	66	1.23E-24	61
NNU_27583	1134	NNU_015225	1041	0.86	562	7.08E-169	321
NNU_27583	1134	NNU_024502	558	0.83	353	2.85E-88	176
NNU_27587	2403	NNU_026333	552	0.88	264	1.02E-85	172
NNU_27587	2403	NNU_021315	669	0.88	126	1.10E-35	82
NNU_27587	2403	NNU_001817	729	0.82	236	6.47E-48	104
NNU_27587	2403	NNU_021125	4062	0.90	111	5.11E-34	79
NNU_27587	2403	NNU_021126	758	0.90	111	5.11E-34	79
NNU_27587	2403	NNU_025099	1215	1.00	30	2.46E-07	31
NNU_27587	2403	NNU_017151	639	0.86	156	3.03E-41	92

NNU_27587	2403	NNU_025458	4304	0.85	127	1.85E-28	69
NNU_27587	2403	NNU_023930	743	0.88	265	1.32E-84	170
NNU_27587	2403	NNU_014225	1257	0.86	118	1.85E-28	69
NNU_27587	2403	NNU_010612	424	0.95	58	6.75E-18	50
NNU_27587	2403	NNU_025156	411	0.93	111	5.07E-39	88
NNU_27587	2403	NNU_025100	1056	0.81	96	5.25E-14	43
NNU_27587	2403	NNU_007918	2702	0.95	56	8.73E-17	48
NNU_27587	2403	NNU_022375	423	0.84	151	1.84E-33	78
NNU_27587	2403	NNU_004165	551	0.82	237	2.31E-52	112
NNU_27587	2403	NNU_007206	747	0.86	76	1.46E-14	44
NNU_27587	2403	NNU_001683	2407	0.94	93	2.38E-32	76
NNU_27587	2403	NNU_021170	498	0.85	118	3.09E-26	65
NNU_27587	2403	NNU_015793	441	1.00	32	1.90E-08	33
NNU_27587	2403	NNU_008167	1470	0.89	173	1.79E-53	114
NNU_27587	2403	NNU_001444	744	0.83	169	3.95E-35	81
NNU_27587	2403	NNU_019042	540	0.80	225	3.05E-36	83
NNU_27587	2403	NNU_001633	924	0.85	150	1.10E-35	82
NNU_27587	2403	NNU_016201	3092	0.83	177	1.41E-39	89
NNU_27587	2403	NNU_004544	413	0.87	196	8.25E-57	120
NNU_27587	2403	NNU_007420	687	0.82	200	3.92E-40	90
NNU_27587	2403	NNU_006479	1491	0.84	92	8.73E-17	48
NNU_27587	2403	NNU_016672	387	1.00	32	1.90E-08	33
NNU_27587	2403	NNU_003572	359	0.82	248	3.86E-50	108
NNU_27587	2403	NNU_022949	352	0.83	100	8.73E-17	48
NNU_27587	2403	NNU_004982	345	0.88	126	3.95E-35	81
NNU_27587	2403	NNU_007322	430	0.83	100	1.88E-18	51
NNU_27587	2403	NNU_007419	350	0.87	90	4.03E-20	54
NNU_27587	2403	NNU_018992	459	0.78	267	1.41E-39	89
NNU_27587	2403	NNU_006747	480	0.85	105	2.41E-22	58
NNU_27587	2403	NNU_013480	687	0.80	267	1.07E-50	109
NNU_27587	2403	NNU_015836	555	0.91	44	1.90E-08	33
NNU_27587	2403	NNU_015825	482	0.86	98	8.67E-22	57
NNU_27587	2403	NNU_015226	534	0.85	98	4.03E-20	54
NNU_27587	2403	NNU_018474	305	0.88	135	1.41E-39	89
NNU_27587	2403	NNU_010144	437	0.86	111	2.39E-27	67
NNU_27587	2403	NNU_013479	632	0.78	301	1.39E-49	107
NNU_27587	2403	NNU_017907	360	0.87	90	4.03E-20	54
NNU_27587	2403	NNU_016987	353	0.78	246	6.61E-33	77
NNU_27587	2403	NNU_025816	339	0.84	90	5.25E-14	43
NNU_27587	2403	NNU_002162	339	0.79	183	2.39E-27	67
NNU_27587	2403	NNU_005976	616	0.82	207	6.51E-43	95
NNU_27587	2403	NNU_015186	287	0.88	128	8.48E-37	84
NNU_27587	2403	NNU_017062	341	0.86	75	5.25E-14	43
NNU_27587	2403	NNU_025369	564	0.86	87	6.75E-18	50

NNU_27587	2403	NNU_021129	1320	0.87	90	4.03E-20	54
NNU_27587	2403	NNU_000752	673	0.83	100	1.88E-18	51
NNU_27587	2403	NNU_025626	420	0.81	94	6.79E-13	41
NNU_27587	2403	NNU_015837	564	0.82	92	1.89E-13	42
NNU_27587	2403	NNU_019043	328	0.85	108	5.18E-24	61
NNU_27587	2403	NNU_004778	380	0.93	58	3.14E-16	47
NNU_27587	2403	NNU_016831	409	0.77	239	2.39E-27	67
NNU_27587	2403	NNU_013908	234	0.86	90	1.88E-18	51
NNU_27587	2403	NNU_019020	344	0.80	124	6.75E-18	50
NNU_27587	2403	NNU_004010	351	0.76	169	3.14E-16	47
NNU_27587	2403	NNU_016433	405	0.88	123	5.11E-34	79
NNU_27587	2403	NNU_021030	837	0.83	98	8.73E-17	48
NNU_27587	2403	NNU_010609	267	0.84	128	6.65E-28	68
NNU_27587	2403	NNU_025207	540	0.79	122	1.13E-15	46
NNU_27587	2403	NNU_024125	225	0.85	104	8.67E-22	57
NNU_27587	2403	NNU_002516	1158	0.90	88	1.44E-24	62
NNU_27587	2403	NNU_022647	499	0.93	87	5.14E-29	70
NNU_27587	2403	NNU_019901	248	0.84	78	2.44E-12	40
NNU_27587	2403	NNU_010664	378	0.78	92	1.90E-08	33
NNU_27587	2403	NNU_019582	441	0.95	77	8.61E-27	66
NNU_27587	2403	NNU_006183	225	0.86	58	1.47E-09	35
NNU_27587	2403	NNU_023030	500	0.97	68	4.00E-25	63
NNU_27587	2403	NNU_000692	588	0.76	204	2.41E-22	58
NNU_27587	2403	NNU_010706	511	0.79	147	1.45E-19	53
NNU_27587	2403	NNU_010886	279	0.80	127	1.88E-18	51
NNU_27587	2403	NNU_003121	619	0.86	87	6.75E-18	50
NNU_27587	2403	NNU_018477	960	0.76	173	2.44E-12	40
NNU_27587	2403	NNU_012332	615	0.85	88	5.25E-14	43
NNU_27587	2403	NNU_017098	386	0.90	59	1.89E-13	42
NNU_27587	2403	NNU_000606	414	0.89	62	1.89E-13	42
NNU_27587	2403	NNU_017705	304	0.76	144	8.79E-12	39
NNU_27587	2403	NNU_017938	277	0.97	36	5.29E-09	34
NNU_27587	2403	NNU_011351	1155	0.97	33	2.46E-07	31
NNU_27588	2323	NNU_012316	729	0.93	269	2.67E-106	209
NNU_27588	2323	NNU_003745	1275	0.91	206	3.60E-75	153
NNU_27590	2368	NNU_026178	1896	0.94	98	3.89E-35	81
NNU_27590	2368	NNU_001897	1028	0.84	113	2.37E-22	58
NNU_27590	2368	NNU_015247	624	0.95	41	4.03E-10	36
NNU_27592	2221	NNU_022014	1998	0.89	144	1.29E-44	98
NNU_27592	2221	NNU_019873	1204	0.88	148	1.67E-43	96
NNU_27592	2221	NNU_017130	705	1.00	33	4.88E-09	34
NNU_27598	4353	NNU_015987	1887	0.92	170	1.49E-61	129
NNU_27598	4353	NNU_006037	1923	0.80	169	5.63E-26	65
NNU_27599	3507	NNU_002015	5061	0.93	552	0	442

NNU_27599	3507	NNU_004779	3289	0.90	196	9.20E-68	140
NNU_27600	817	NNU_021561	1383	0.96	113	3.61E-46	100
NNU_27601	3848	NNU_015919	6104	0.93	82	4.97E-26	65
NNU_27601	3848	NNU_000970	2641	0.93	56	6.52E-15	45
NNU_27603	968	NNU_023709	1481	0.80	588	3.89E-116	226
NNU_27605	949	NNU_014922	1433	0.88	168	1.51E-45	99
NNU_27607	3608	NNU_023483	327	0.96	87	9.94E-33	77
NNU_27609	1038	NNU_022546	842	0.96	44	3.75E-12	39
NNU_27611	1569	NNU_024580	2493	0.86	491	6.10E-146	280
NNU_27615	1868	NNU_014495	1994	0.92	62	6.76E-17	48
NNU_27616	1184	NNU_003058	3282	0.94	205	6.45E-85	170
NNU_27616	1184	NNU_013225	2031	0.87	258	1.10E-72	148
NNU_27616	1184	NNU_017408	1737	0.88	119	8.96E-34	78
NNU_27620	2599	NNU_020668	4831	0.83	1045	0	498
NNU_27620	2599	NNU_017676	734	0.90	174	5.34E-59	124
NNU_27624	2718	NNU_009055	2313	0.95	117	1.23E-45	100
NNU_27626	1991	NNU_012859	939	0.93	392	2.73E-160	306
NNU_27633	1507	NNU_010827	2468	0.92	78	3.23E-24	61
NNU_27636	2181	NNU_021310	2371	0.98	108	2.11E-47	103
NNU_27639	1418	NNU_002434	708	0.85	295	1.30E-82	166
NNU_27640	7260	NNU_023248	3440	0.95	149	1.16E-59	126
NNU_27642	2338	NNU_020402	1894	0.83	246	3.73E-55	117
NNU_27642	2338	NNU_017877	1472	0.87	110	1.80E-28	69
NNU_27642	2338	NNU_019144	2645	0.89	78	1.82E-18	51
NNU_27642	2338	NNU_017273	368	0.83	68	1.85E-08	33
NNU_27642	2338	NNU_014471	4021	0.89	43	8.61E-07	30
NNU_27647	5299	NNU_015858	2227	0.95	106	1.87E-41	93
NNU_27647	5299	NNU_000462	630	1.00	29	1.96E-06	30
NNU_27650	1781	NNU_017478	2936	0.93	138	7.93E-51	109
NNU_27650	1781	NNU_007466	423	0.95	54	8.34E-16	46
NNU_27653	2543	NNU_025522	876	0.93	122	6.90E-43	95
NNU_27654	5113	NNU_022260	1308	0.85	176	1.39E-42	95
NNU_27654	5113	NNU_011450	2220	0.84	176	5.01E-42	94
NNU_27657	4209	NNU_022274	1099	0.95	334	1.28E-146	282
NNU_27657	4209	NNU_001967	4057	0.97	272	1.31E-126	246
NNU_27657	4209	NNU_010315	1133	0.96	252	3.73E-112	220
NNU_27657	4209	NNU_013035	1521	0.95	252	8.07E-109	214
NNU_27657	4209	NNU_004639	651	0.94	252	3.76E-107	211
NNU_27657	4209	NNU_013555	198	0.97	108	1.90E-45	100
NNU_27657	4209	NNU_012123	1374	0.98	95	6.89E-40	90
NNU_27660	6674	NNU_017502	4259	0.93	131	6.50E-47	103
NNU_27661	1632	NNU_017478	2936	0.93	138	7.26E-51	109
NNU_27661	1632	NNU_007466	423	0.95	54	7.63E-16	46
NNU_27668	1057	NNU_007378	1017	0.90	164	2.16E-54	115

NNU_27669	1291	NNU_011955	1354	0.93	68	2.15E-20	54
NNU_27673	2348	NNU_020635	955	0.87	361	4.45E-114	223
NNU_27673	2348	NNU_016892	651	0.93	149	3.75E-55	117
NNU_27673	2348	NNU_021346	1875	0.82	78	3.99E-10	36
NNU_27673	2348	NNU_015263	879	1.00	31	6.68E-08	32
NNU_27674	2084	NNU_020629	465	0.95	263	3.05E-115	225
NNU_27674	2084	NNU_017155	656	0.88	324	5.14E-108	212
NNU_27674	2084	NNU_016040	2194	0.99	85	2.64E-36	83
NNU_27674	2084	NNU_010522	996	0.92	70	1.26E-19	53
NNU_27677	974	NNU_016692	1244	0.93	971	0	774
NNU_27680	4519	NNU_005729	1706	0.90	87	9.78E-24	61
NNU_27680	4519	NNU_014878	2550	0.96	54	1.65E-16	48
NNU_27680	4519	NNU_011461	2998	0.78	102	1.29E-07	32
NNU_27681	792	NNU_008440	3497	0.82	135	2.80E-22	57
NNU_27682	2049	NNU_015235	891	0.97	89	3.36E-35	81
NNU_27682	2049	NNU_022533	223	1.00	49	5.74E-18	50
NNU_27682	2049	NNU_014093	303	0.96	53	2.67E-16	47
NNU_27682	2049	NNU_020882	885	0.96	45	5.78E-13	41
NNU_27684	948	NNU_014476	1638	0.90	157	3.23E-52	111
NNU_27684	948	NNU_023822	1630	0.92	131	3.25E-47	102
NNU_27686	944	NNU_006462	1350	0.82	219	9.00E-48	103
NNU_27689	888	NNU_002303	1584	0.92	100	2.40E-33	77
NNU_27689	888	NNU_007886	855	0.83	246	8.33E-58	121
NNU_27689	888	NNU_002301	2170	0.90	168	1.39E-55	117
NNU_27689	888	NNU_026274	1977	0.89	100	2.42E-28	68
NNU_27689	888	NNU_016111	568	0.99	68	3.13E-27	66
NNU_27691	1038	NNU_022560	1183	0.91	159	4.55E-56	118
NNU_27692	983	NNU_021251	2214	0.93	405	1.70E-169	322
NNU_27692	983	NNU_011226	2535	0.82	441	3.12E-102	201
NNU_27692	983	NNU_011227	488	0.98	56	1.63E-20	54
NNU_27696	2753	NNU_006076	2741	0.91	267	5.34E-99	196
NNU_27699	2668	NNU_014662	3165	0.97	222	1.43E-104	206
NNU_27699	2668	NNU_003926	4078	0.96	107	2.01E-43	96
NNU_27699	2668	NNU_001382	2592	0.98	63	5.76E-24	61
NNU_27699	2668	NNU_002436	2286	0.96	65	9.63E-22	57
NNU_27701	1395	NNU_020911	1942	0.83	465	2.64E-109	214
NNU_27701	1395	NNU_024020	675	0.83	238	4.78E-52	111
NNU_27701	1395	NNU_001772	894	0.88	160	1.04E-48	105
NNU_27702	2545	NNU_019707	3183	0.86	105	5.49E-24	61
NNU_27702	2545	NNU_000908	1704	1.00	28	3.37E-06	29
NNU_27703	1544	NNU_022743	5044	0.85	422	3.77E-113	221
NNU_27704	377	NNU_020501	1689	0.88	126	1.63E-36	82
NNU_27705	787	NNU_002086	3983	0.94	121	3.47E-46	100
NNU_27705	787	NNU_013333	4081	0.97	38	1.31E-10	36

NNU_27706	2398	NNU_012991	889	0.84	342	1.01E-90	181
NNU_27709	824	NNU_002746	588	0.85	324	9.50E-92	182
NNU_27710	1482	NNU_002734	1272	0.77	617	4.84E-87	174
NNU_27713	4432	NNU_009407	1035	0.88	451	6.21E-150	288
NNU_27713	4432	NNU_017435	1029	0.87	86	7.47E-20	54
NNU_27714	1696	NNU_021251	2214	0.93	405	2.96E-169	322
NNU_27714	1696	NNU_011227	488	0.94	282	4.11E-118	230
NNU_27714	1696	NNU_011226	2535	0.94	124	1.63E-47	103
NNU_27714	1696	NNU_007456	3350	0.90	162	1.62E-52	112
NNU_27714	1696	NNU_023931	3240	0.88	93	2.19E-21	56
NNU_27715	900	NNU_017482	1358	0.94	99	4.04E-36	82
NNU_27715	900	NNU_012576	1794	0.93	82	1.14E-26	65
NNU_27720	852	NNU_011649	1149	0.89	220	1.01E-71	146
NNU_27723	3528	NNU_008009	1538	0.91	269	3.19E-97	193
NNU_27723	3528	NNU_016581	2166	0.93	262	1.13E-106	210
NNU_27723	3528	NNU_007143	428	0.90	87	7.62E-24	61
NNU_27723	3528	NNU_000248	1086	0.91	181	1.56E-60	127
NNU_27723	3528	NNU_013013	1606	0.76	216	4.59E-21	56
NNU_27724	1118	NNU_026054	1862	0.90	401	2.01E-144	277
NNU_27724	1118	NNU_021184	740	0.93	202	2.85E-78	158
NNU_27724	1118	NNU_008267	1284	0.91	194	8.04E-69	141
NNU_27724	1118	NNU_011752	551	0.97	99	5.01E-41	91
NNU_27730	869	NNU_008028	788	0.91	138	2.30E-48	104
NNU_27730	869	NNU_014978	3620	0.88	80	6.67E-19	51
NNU_27733	591	NNU_018421	1628	0.87	450	1.76E-137	264
NNU_27734	879	NNU_014626	1159	0.95	175	2.24E-73	149
NNU_27735	616	NNU_014616	819	0.85	256	3.36E-70	143
NNU_27736	5465	NNU_016879	1170	0.81	141	4.26E-23	60
NNU_27738	1083	NNU_015982	322	0.84	315	1.28E-81	164
NNU_27740	2504	NNU_002653	2707	0.83	117	3.25E-21	56
NNU_27740	2504	NNU_011106	3772	0.80	117	3.27E-16	47
NNU_27740	2504	NNU_022917	2152	0.92	65	1.96E-18	51
NNU_27740	2504	NNU_000944	2282	0.78	117	3.30E-11	38
NNU_27740	2504	NNU_006968	1126	1.00	34	1.53E-09	35
NNU_27740	2504	NNU_000616	1798	0.97	36	5.51E-09	34
NNU_27740	2504	NNU_020232	1772	1.00	29	9.23E-07	30
NNU_27746	844	NNU_013963	1894	0.87	504	1.49E-154	295
NNU_27748	506	NNU_024326	1806	0.97	174	4.53E-78	157
NNU_27748	506	NNU_023166	679	0.94	180	4.56E-73	148
NNU_27751	608	NNU_020635	955	1.00	39	6.00E-13	40
NNU_27751	608	NNU_015481	1327	0.98	39	2.79E-11	37
NNU_27751	608	NNU_009893	1567	0.90	112	3.48E-35	80
NNU_27751	608	NNU_023123	1803	0.93	88	3.51E-30	71
NNU_27751	608	NNU_021346	1875	1.00	70	3.51E-30	71

NNU_27751	608	NNU_003163	4594	0.97	59	9.90E-21	54
NNU_27751	608	NNU_016892	651	0.90	58	4.64E-14	42
NNU_27751	608	NNU_008505	1910	1.00	39	6.00E-13	40
NNU_27757	890	NNU_006250	819	0.89	375	9.71E-132	254
NNU_27757	890	NNU_012140	1428	0.98	131	3.86E-61	127
NNU_27757	890	NNU_017618	518	0.95	128	3.02E-52	111
NNU_27764	712	NNU_007210	1792	0.91	430	1.23E-164	313
NNU_27764	712	NNU_008743	2063	0.88	474	1.60E-158	302
NNU_27766	955	NNU_014080	852	0.77	186	9.43E-23	58
NNU_27766	955	NNU_022480	864	0.89	108	4.33E-31	73
NNU_27767	955	NNU_015836	555	0.77	194	7.34E-19	51
NNU_27767	955	NNU_004165	551	0.75	230	2.64E-18	50
NNU_27767	955	NNU_011522	478	0.78	154	1.23E-16	47
NNU_27767	955	NNU_023441	708	0.78	154	4.42E-16	46
NNU_27767	955	NNU_002162	339	0.76	209	1.59E-15	45
NNU_27767	955	NNU_015837	564	0.77	148	2.66E-13	41
NNU_27773	888	NNU_019144	2645	0.91	248	1.03E-91	182
NNU_27773	888	NNU_011357	700	0.78	165	3.17E-17	48
NNU_27773	888	NNU_010333	868	0.98	51	8.82E-18	49
NNU_27775	5032	NNU_021231	543	0.83	205	6.33E-46	101
NNU_27775	5032	NNU_016970	1221	0.99	74	8.36E-30	72
NNU_27775	5032	NNU_023731	204	0.96	45	5.14E-12	40
NNU_27775	5032	NNU_000795	6599	0.97	31	6.70E-06	29
NNU_27776	1461	NNU_000353	2331	0.96	266	4.54E-122	237
NNU_27777	1535	NNU_023123	1803	0.95	80	1.17E-28	69
NNU_27778	1723	NNU_004089	1847	0.95	172	1.24E-73	150
NNU_27778	1723	NNU_014193	2476	0.98	168	1.59E-77	157
NNU_27779	3007	NNU_015881	1614	0.96	178	7.77E-78	158
NNU_27779	3007	NNU_008078	492	0.82	106	6.59E-14	43
NNU_27780	902	NNU_011418	633	0.98	376	0	359
NNU_27780	902	NNU_016490	492	0.86	185	1.43E-50	108
NNU_27781	5156	NNU_021231	543	0.97	37	3.17E-09	35
NNU_27781	5156	NNU_016970	1221	0.99	74	8.57E-30	72
NNU_27781	5156	NNU_023731	204	0.96	45	5.27E-12	40
NNU_27781	5156	NNU_000795	6599	0.97	31	6.87E-06	29
NNU_27783	731	NNU_002064	1194	0.87	345	2.30E-102	201
NNU_27783	731	NNU_011608	1282	0.88	177	6.86E-53	112
NNU_27783	731	NNU_011752	551	0.96	51	3.36E-16	46
NNU_27783	731	NNU_026054	1862	0.93	54	1.56E-14	43
NNU_27786	950	NNU_002064	1194	0.94	152	1.48E-60	126
NNU_27786	950	NNU_021357	1912	0.88	68	1.58E-15	45
NNU_27790	1723	NNU_013064	905	0.98	44	1.35E-13	42
NNU_27791	2504	NNU_014507	2971	0.94	376	1.24E-159	305
NNU_27793	573	NNU_018538	561	0.98	58	7.21E-22	56

NNU_27793	573	NNU_002714	1336	0.98	58	7.21E-22	56
NNU_27799	6404	NNU_018705	1149	0.90	314	5.69E-112	220
NNU_27799	6404	NNU_026246	2310	0.90	314	1.23E-108	214
NNU_27799	6404	NNU_025786	2578	0.87	351	5.73E-107	211
NNU_27799	6404	NNU_018711	2412	0.83	404	2.09E-96	192
NNU_27799	6404	NNU_006009	2228	0.89	233	1.00E-74	153
NNU_27799	6404	NNU_006709	3256	0.84	297	3.60E-74	152
NNU_27799	6404	NNU_021593	2310	0.91	125	2.26E-41	93
NNU_27799	6404	NNU_019670	1557	0.93	103	8.18E-36	83
NNU_27799	6404	NNU_020739	1559	0.88	118	1.77E-32	77
NNU_27799	6404	NNU_018712	1788	0.96	75	6.41E-27	67
NNU_27799	6404	NNU_020985	2709	0.86	113	2.31E-26	66
NNU_27799	6404	NNU_001676	1042	0.93	72	2.32E-21	57
NNU_27799	6404	NNU_014349	2377	0.93	44	3.94E-09	35
NNU_27820	4395	NNU_003312	837	0.92	131	2.57E-44	98
NNU_27820	4395	NNU_020638	249	0.95	54	2.07E-15	46
NNU_27823	776	NNU_024384	3239	0.90	179	1.20E-60	126
NNU_27824	1264	NNU_002281	4928	0.94	104	3.42E-38	86
NNU_27825	247	NNU_025710	3204	0.94	108	1.03E-41	91
NNU_27829	1075	NNU_024551	1782	0.96	68	8.23E-24	60
NNU_27830	1927	NNU_005606	2043	0.92	777	0	586
NNU_27833	1272	NNU_019303	339	0.89	104	9.71E-29	69
NNU_27833	1272	NNU_026178	1896	0.93	42	2.78E-09	34
NNU_27843	3782	NNU_011432	1004	0.81	201	3.72E-37	85
NNU_27843	3782	NNU_009020	2526	0.92	109	4.82E-36	83
NNU_27843	3782	NNU_011126	1541	0.97	35	3.00E-08	33
NNU_27843	3782	NNU_020344	1952	0.87	69	8.29E-14	43
NNU_27845	686	NNU_010005	615	0.93	53	5.26E-14	42
NNU_27846	2548	NNU_025974	968	1.00	46	3.33E-16	47
NNU_27849	966	NNU_015440	669	0.80	541	2.35E-108	212
NNU_27851	2342	NNU_016670	3038	0.96	997	0	877
NNU_27851	2342	NNU_014847	3105	0.85	993	0	555
NNU_27851	2342	NNU_004264	4121	0.93	315	2.02E-127	247
NNU_27851	2342	NNU_003438	1074	0.97	36	5.15E-09	34
NNU_27853	1170	NNU_014427	1700	0.78	665	6.11E-115	224
NNU_27854	664	NNU_004683	602	0.93	99	1.37E-34	79
NNU_27854	664	NNU_024088	591	0.85	265	2.19E-67	138
NNU_27854	664	NNU_000669	3070	0.93	74	5.01E-24	60
NNU_27854	664	NNU_009077	111	0.95	57	6.53E-18	49
NNU_27854	664	NNU_024089	966	0.96	53	2.35E-17	48
NNU_27858	3887	NNU_020201	507	0.93	121	2.27E-44	98
NNU_27858	3887	NNU_022817	467	0.93	73	1.09E-22	59
NNU_27858	3887	NNU_023071	2152	0.97	38	6.64E-10	36
NNU_27858	3887	NNU_021066	480	0.95	75	1.81E-25	64

NNU_27858	3887	NNU_022816	291	0.98	65	6.50E-25	63
NNU_27858	3887	NNU_010033	443	0.96	54	3.94E-17	49
NNU_27859	2287	NNU_016820	1452	1.00	28	3.03E-06	29
NNU_27864	4203	NNU_015465	2401	0.95	860	0	737
NNU_27864	4203	NNU_021070	1625	0.93	134	8.78E-49	106
NNU_27864	4203	NNU_026054	1862	0.88	156	2.46E-44	98
NNU_27865	6840	NNU_005451	2106	0.91	45	1.52E-08	34
NNU_27865	6840	NNU_014471	4021	1.00	32	5.45E-08	33
NNU_27867	702	NNU_001927	1219	0.90	187	5.02E-64	132
NNU_27868	2687	NNU_022726	2539	0.86	306	8.85E-87	174
NNU_27869	6584	NNU_005451	2106	0.91	45	1.46E-08	34
NNU_27869	6584	NNU_014471	4021	1.00	32	5.24E-08	33
NNU_27871	2538	NNU_016010	481	0.88	281	1.39E-89	179
NNU_27871	2538	NNU_005650	1854	0.94	84	5.43E-29	70
NNU_27876	340	NNU_024814	408	0.94	100	4.06E-37	83
NNU_27876	340	NNU_014813	807	0.95	55	4.18E-17	47
NNU_27876	340	NNU_016676	456	0.96	45	3.25E-13	40
NNU_27881	4637	NNU_000710	1088	0.97	96	3.53E-38	87
NNU_27881	4637	NNU_007596	462	0.89	96	7.76E-25	63
NNU_27883	2004	NNU_007872	3355	0.86	448	1.72E-132	256
NNU_27884	1770	NNU_024711	1437	0.81	88	1.08E-09	35
NNU_27894	1910	NNU_011403	537	0.90	230	1.37E-78	159
NNU_27895	1191	NNU_005567	1281	0.83	544	6.05E-135	260
NNU_27895	1191	NNU_016126	1035	0.80	704	9.91E-148	283
NNU_27895	1191	NNU_014558	1029	0.88	110	1.95E-30	72
NNU_27897	1508	NNU_018867	690	0.92	154	4.00E-53	113
NNU_27898	7574	NNU_005503	2607	0.76	285	1.26E-29	72
NNU_27898	7574	NNU_010865	6040	0.87	126	5.86E-28	69
NNU_27898	7574	NNU_008830	5597	0.82	149	4.56E-24	62
NNU_27906	1440	NNU_018867	690	0.92	155	1.06E-53	114
NNU_27908	1978	NNU_005580	1328	0.96	90	1.51E-33	78
NNU_27908	1978	NNU_016234	923	0.94	88	2.52E-31	74
NNU_27909	1102	NNU_016234	923	0.87	195	1.04E-57	121
NNU_27909	1102	NNU_025746	1260	0.87	173	1.75E-50	108
NNU_27909	1102	NNU_000166	4670	0.94	115	2.95E-43	95
NNU_27909	1102	NNU_011207	952	0.90	116	1.79E-35	81
NNU_27909	1102	NNU_018809	2653	0.88	116	3.87E-32	75
NNU_27909	1102	NNU_015540	999	0.85	132	1.80E-30	72
NNU_27909	1102	NNU_024930	1176	0.97	70	1.40E-26	65
NNU_27911	747	NNU_025063	931	0.92	200	1.13E-75	153
NNU_27911	747	NNU_010734	936	0.93	197	5.24E-79	159
NNU_27911	747	NNU_025640	372	0.92	185	1.14E-70	144
NNU_27911	747	NNU_022054	381	0.93	131	5.46E-49	105
NNU_27912	2000	NNU_018778	1633	0.87	186	4.12E-54	115

NNU_27912	2000	NNU_011285	1338	0.84	166	5.45E-38	86
NNU_27913	2756	NNU_021132	403	0.93	110	2.09E-38	87
NNU_27914	952	NNU_002701	846	0.85	395	8.28E-113	220
NNU_27916	1163	NNU_014391	1290	0.83	221	2.41E-44	97
NNU_27917	2967	NNU_002701	846	0.98	44	2.34E-13	42
NNU_27919	1865	NNU_021207	1461	0.96	155	6.29E-67	138
NNU_27919	1865	NNU_006798	2454	0.83	196	1.40E-43	96
NNU_27919	1865	NNU_011863	1312	0.93	115	8.43E-41	91
NNU_27919	1865	NNU_011624	426	0.94	93	1.84E-32	76
NNU_27919	1865	NNU_026142	702	0.88	112	8.55E-31	73
NNU_27919	1865	NNU_016768	1308	0.93	88	1.11E-29	71
NNU_27919	1865	NNU_023275	669	0.98	60	1.86E-22	58
NNU_27919	1865	NNU_020594	1389	0.90	78	8.67E-21	55
NNU_27921	2521	NNU_025028	1847	0.90	216	1.09E-75	154
NNU_27921	2521	NNU_000649	1353	0.93	112	1.48E-39	89
NNU_27921	2521	NNU_007612	1801	0.85	276	1.82E-73	150
NNU_27923	1872	NNU_025089	1671	0.90	342	3.49E-124	241
NNU_27924	2495	NNU_015940	766	1.00	32	1.98E-08	33
NNU_27924	2495	NNU_002813	1845	0.97	35	1.98E-08	33
NNU_27924	2495	NNU_013804	5355	0.97	35	1.98E-08	33
NNU_27924	2495	NNU_012525	2229	1.00	31	7.11E-08	32
NNU_27924	2495	NNU_006133	1971	0.95	35	9.19E-07	30
NNU_27930	279	NNU_011677	240	0.95	202	3.05E-87	173
NNU_27935	368	NNU_026208	2283	0.91	116	1.23E-37	84
NNU_27941	5402	NNU_018290	4967	0.79	524	2.31E-85	172
NNU_27941	5402	NNU_008990	1250	0.79	253	5.29E-42	94
NNU_27941	5402	NNU_024338	2566	0.92	107	8.92E-35	81
NNU_27941	5402	NNU_004797	1906	0.91	88	5.41E-27	67
NNU_27941	5402	NNU_015993	3325	0.87	100	9.05E-25	63
NNU_27941	5402	NNU_022137	4555	0.96	70	3.25E-24	62
NNU_27941	5402	NNU_005327	2341	0.92	82	3.25E-24	62
NNU_27941	5402	NNU_016507	1238	0.84	98	5.48E-17	49
NNU_27941	5402	NNU_001653	1563	0.86	78	2.55E-15	46
NNU_27942	1002	NNU_020887	1011	0.95	352	1.77E-154	295
NNU_27942	1002	NNU_019388	3006	1.00	32	7.83E-09	33
NNU_27947	1639	NNU_007519	1714	0.95	65	5.88E-22	57
NNU_27948	972	NNU_011450	2220	0.96	56	7.48E-19	51
NNU_27948	972	NNU_018295	1659	0.89	53	1.63E-10	36
NNU_27949	3800	NNU_015919	6104	0.95	36	3.90E-07	31
NNU_27950	2434	NNU_009733	465	0.87	180	2.34E-52	112
NNU_27955	6555	NNU_022546	842	0.97	66	1.42E-23	61
NNU_27955	6555	NNU_012624	2304	0.93	53	5.18E-13	42
NNU_27956	1615	NNU_004937	2734	0.90	255	2.44E-90	180
NNU_27956	1615	NNU_014046	678	0.88	255	2.48E-80	162

NNU_27956	1615	NNU_010614	2304	0.95	41	2.73E-10	36
NNU_27957	2401	NNU_018049	1542	0.90	170	8.24E-57	120
NNU_27959	866	NNU_022613	929	0.88	232	2.86E-72	147
NNU_27960	781	NNU_001534	1822	0.86	338	1.92E-98	194
NNU_27961	1885	NNU_010935	908	0.87	321	7.83E-101	199
NNU_27962	615	NNU_024473	866	0.91	150	2.67E-51	109
NNU_27963	1261	NNU_007430	3771	0.98	98	4.38E-42	93
NNU_27963	1261	NNU_007412	1770	0.76	485	2.56E-59	124
NNU_27969	4321	NNU_002559	3905	0.81	656	1.32E-141	273
NNU_27969	4321	NNU_019253	977	0.98	210	6.50E-100	198
NNU_27970	420	NNU_010836	1176	0.98	102	3.02E-44	96
NNU_27971	967	NNU_017710	998	0.95	188	1.47E-80	162
NNU_27971	967	NNU_022987	1672	0.96	202	2.42E-88	176
NNU_27971	967	NNU_010778	2015	0.91	187	1.16E-66	137
NNU_27971	967	NNU_018356	1248	0.95	92	7.28E-34	78
NNU_27972	840	NNU_017936	2380	0.79	372	1.68E-64	133
NNU_27972	840	NNU_017941	2109	0.78	372	1.69E-59	124
NNU_27977	1742	NNU_010696	1587	0.93	278	7.13E-111	217
NNU_27977	1742	NNU_008882	1110	0.94	65	1.05E-19	53
NNU_27979	1036	NNU_026103	570	0.90	406	1.43E-150	288
NNU_27979	1036	NNU_017976	571	1.00	29	3.77E-07	30
NNU_27980	3533	NNU_000638	2162	0.91	148	7.37E-49	106
NNU_27980	3533	NNU_012838	2072	0.75	452	3.43E-47	103
NNU_27980	3533	NNU_002583	882	0.97	36	7.80E-09	34
NNU_27982	1543	NNU_007987	2895	0.92	329	7.92E-130	251
NNU_27985	1244	NNU_005602	5230	0.93	275	1.09E-112	220
NNU_27985	1244	NNU_004123	1319	0.93	99	2.62E-34	79
NNU_27985	1244	NNU_003085	1122	0.91	99	5.67E-31	73
NNU_27986	984	NNU_006803	487	0.89	82	1.26E-21	56
NNU_27987	528	NNU_019316	295	0.92	224	7.81E-86	171
NNU_27987	528	NNU_006803	487	0.91	226	4.70E-83	166
NNU_27996	2848	NNU_019794	1320	0.91	312	5.38E-119	232
NNU_27997	1017	NNU_016317	984	1.00	60	2.16E-24	61
NNU_27998	399	NNU_025284	195	0.96	194	1.62E-86	172
NNU_27999	580	NNU_014389	8136	0.97	35	4.46E-09	33
NNU_28000	1287	NNU_004903	681	0.99	104	4.44E-47	102
NNU_28000	1287	NNU_014389	8136	0.96	171	7.13E-75	152
NNU_28000	1287	NNU_005761	1869	0.97	128	5.67E-56	118
NNU_28000	1287	NNU_023515	351	0.98	104	2.07E-45	99
NNU_28000	1287	NNU_020709	1813	0.94	106	2.69E-39	88
NNU_28000	1287	NNU_026156	834	0.96	76	3.53E-28	68
NNU_28000	1287	NNU_020268	1368	0.96	74	4.57E-27	66
NNU_28000	1287	NNU_009885	747	0.95	61	7.70E-20	53
NNU_28000	1287	NNU_013956	2400	0.98	46	7.76E-15	44

NNU_28005	849	NNU_013915	912	0.93	183	7.79E-73	148
NNU_28005	849	NNU_013911	1175	0.96	162	1.69E-69	142
NNU_28006	2227	NNU_000013	1668	0.94	483	0	391
NNU_28006	2227	NNU_011224	1452	0.93	101	3.66E-35	81
NNU_28009	885	NNU_024185	522	0.97	257	2.12E-118	230
NNU_28012	4740	NNU_020826	1551	0.89	463	1.82E-160	307
NNU_28012	4740	NNU_025184	609	0.93	365	3.09E-148	285
NNU_28012	4740	NNU_009405	772	0.87	390	1.16E-117	230
NNU_28012	4740	NNU_023562	582	0.87	429	8.84E-129	250
NNU_28012	4740	NNU_010526	3510	0.94	226	1.20E-92	185
NNU_28012	4740	NNU_023613	2535	0.88	260	3.39E-83	168
NNU_28012	4740	NNU_019873	1204	0.88	152	7.71E-45	99
NNU_28012	4740	NNU_023222	1590	0.85	163	1.00E-38	88
NNU_28012	4740	NNU_017130	705	1.00	39	4.84E-12	40
NNU_28015	2192	NNU_017850	1275	0.84	1166	0	592
NNU_28015	2192	NNU_024338	2566	0.81	112	1.72E-13	42
NNU_28015	2192	NNU_015810	2059	0.97	38	3.73E-10	36
NNU_28016	1590	NNU_018901	675	0.90	395	4.81E-142	273
NNU_28017	818	NNU_006542	9798	0.90	180	1.27E-60	126
NNU_28017	818	NNU_008586	711	0.90	159	2.77E-52	111
NNU_28017	818	NNU_004532	612	0.90	121	7.88E-38	85
NNU_28017	818	NNU_015654	3309	0.94	59	8.10E-18	49
NNU_28017	818	NNU_025024	631	0.97	37	4.91E-10	35
NNU_28026	763	NNU_004905	888	0.90	95	2.67E-27	66
NNU_28027	1254	NNU_003058	3282	0.96	208	3.14E-93	185
NNU_28027	1254	NNU_004143	3876	0.84	136	7.40E-30	71
NNU_28027	1254	NNU_017408	1737	0.85	119	4.45E-27	66
NNU_28027	1254	NNU_013225	2031	1.00	28	1.65E-06	29
NNU_28035	1692	NNU_010668	285	0.93	94	2.15E-31	74
NNU_28037	2756	NNU_016448	285	0.98	53	2.15E-18	51
NNU_28037	2756	NNU_009220	1218	0.85	88	7.75E-18	50
NNU_28041	1386	NNU_023274	1008	0.90	87	2.96E-24	61
NNU_28042	872	NNU_006458	2037	0.90	146	2.98E-47	102
NNU_28042	872	NNU_024143	1987	0.80	182	5.10E-30	71
NNU_28046	5625	NNU_012851	1700	0.76	345	1.18E-43	97
NNU_28047	880	NNU_014663	1895	0.83	435	3.58E-106	208
NNU_28048	4837	NNU_026078	762	0.88	91	1.35E-22	59
NNU_28050	839	NNU_025445	624	0.93	628	0	486
NNU_28050	839	NNU_025444	309	0.94	308	1.53E-129	250
NNU_28050	839	NNU_023992	306	0.95	292	5.50E-129	249
NNU_28050	839	NNU_025443	306	0.94	292	5.54E-124	240
NNU_28050	839	NNU_025448	372	0.98	171	1.64E-79	160
NNU_28051	2066	NNU_012761	1967	0.94	69	2.07E-22	58
NNU_28051	2066	NNU_012760	2111	0.93	70	2.67E-21	56

NNU_28051	2066	NNU_010220	1947	0.97	34	5.87E-08	32
NNU_28054	742	NNU_019571	3398	0.81	500	3.88E-105	206
NNU_28056	935	NNU_019775	2365	0.95	419	0	354
NNU_28056	935	NNU_011700	1031	0.91	237	8.42E-88	175
NNU_28056	935	NNU_011752	551	0.92	124	2.49E-43	95
NNU_28056	935	NNU_008267	1284	0.88	124	2.53E-33	77
NNU_28061	1599	NNU_004250	3927	0.96	255	3.02E-114	223
NNU_28062	811	NNU_007943	5273	0.94	271	1.95E-113	221
NNU_28062	811	NNU_002634	453	0.93	271	4.22E-110	215
NNU_28062	811	NNU_025377	894	0.93	124	4.63E-45	98
NNU_28064	2838	NNU_020144	405	0.88	123	6.04E-34	79
NNU_28068	1408	NNU_007743	1584	0.97	127	8.02E-55	116
NNU_28068	1408	NNU_023406	769	0.88	252	3.60E-78	158
NNU_28068	1408	NNU_012260	1158	0.89	201	2.85E-64	133
NNU_28069	2215	NNU_013706	3309	0.93	586	0	455
NNU_28069	2215	NNU_002333	1491	0.89	456	5.06E-163	311
NNU_28069	2215	NNU_021022	1485	0.81	456	2.58E-96	191
NNU_28069	2215	NNU_021804	1767	0.87	139	7.81E-37	84
NNU_28069	2215	NNU_010547	6438	0.96	72	1.03E-25	64
NNU_28071	2738	NNU_020520	3654	0.82	142	5.91E-24	61
NNU_28073	3624	NNU_026078	762	0.88	86	6.10E-20	54
NNU_28075	933	NNU_021310	2371	0.88	128	1.51E-35	81
NNU_28075	933	NNU_023841	1522	0.83	134	3.29E-27	66
NNU_28075	933	NNU_003672	516	0.89	95	4.26E-26	64
NNU_28081	2885	NNU_007732	4012	0.90	274	7.25E-98	194
NNU_28081	2885	NNU_019632	501	0.94	144	1.28E-55	118
NNU_28081	2885	NNU_022071	1878	0.92	106	1.71E-34	80
NNU_28081	2885	NNU_017147	2032	0.95	79	2.88E-27	67
NNU_28081	2885	NNU_022433	1819	0.93	70	3.75E-21	56
NNU_28081	2885	NNU_022055	324	0.96	56	2.26E-18	51
NNU_28081	2885	NNU_010316	2507	0.85	84	4.88E-15	45
NNU_28081	2885	NNU_011193	1926	0.97	36	6.36E-09	34
NNU_28084	4137	NNU_025600	3060	0.73	936	1.38E-81	165
NNU_28085	615	NNU_015767	492	0.92	95	2.75E-31	73
NNU_28085	615	NNU_004531	810	0.90	85	4.63E-24	60
NNU_28088	1859	NNU_010907	540	0.87	294	4.68E-93	185
NNU_28088	1859	NNU_025759	738	0.81	281	3.80E-59	124
NNU_28090	2848	NNU_021251	2214	0.95	335	3.10E-146	281
NNU_28090	2848	NNU_023931	3240	1.00	33	6.28E-09	34
NNU_28090	2848	NNU_011227	488	0.97	214	7.15E-98	194
NNU_28090	2848	NNU_011226	2535	0.95	94	1.69E-34	80
NNU_28090	2848	NNU_007456	3350	0.90	159	7.62E-53	113
NNU_28091	2512	NNU_008009	1538	0.93	645	0	503
NNU_28091	2512	NNU_007143	428	0.89	388	1.30E-129	251

NNU_28091	2512	NNU_013013	1606	0.80	641	1.68E-128	249
NNU_28091	2512	NNU_016581	2166	0.92	262	3.77E-100	198
NNU_28091	2512	NNU_000248	1086	0.91	170	1.85E-58	123
NNU_28096	2007	NNU_002301	2170	0.84	298	3.11E-75	153
NNU_28096	2007	NNU_002357	3025	0.95	458	0	398
NNU_28096	2007	NNU_001398	696	0.96	129	4.14E-54	115
NNU_28096	2007	NNU_010719	967	0.93	182	6.73E-72	147
NNU_28096	2007	NNU_016111	568	0.95	130	2.49E-51	110
NNU_28096	2007	NNU_012743	222	0.89	158	1.16E-49	107
NNU_28096	2007	NNU_002303	1584	0.94	124	5.39E-48	104
NNU_28096	2007	NNU_011620	2976	0.74	486	6.97E-47	102
NNU_28098	884	NNU_009714	345	0.92	153	1.80E-54	115
NNU_28098	884	NNU_011739	192	0.90	144	8.41E-48	103
NNU_28100	3990	NNU_000967	5446	0.83	393	6.04E-95	189
NNU_28100	3990	NNU_006722	2107	0.84	144	1.84E-30	73
NNU_28100	3990	NNU_004737	1642	0.90	88	2.40E-24	62
NNU_28100	3990	NNU_004292	4400	0.84	84	8.75E-14	43
NNU_28102	3974	NNU_024473	866	0.95	142	1.06E-57	122
NNU_28108	938	NNU_010229	271	0.98	171	1.84E-79	160
NNU_28108	938	NNU_011446	1556	0.93	199	2.38E-78	158
NNU_28108	938	NNU_024547	800	0.97	127	5.30E-55	116
NNU_28111	2793	NNU_021251	2214	0.95	335	3.04E-146	281
NNU_28111	2793	NNU_011226	2535	0.95	91	7.69E-33	77
NNU_28111	2793	NNU_007456	3350	0.90	159	7.47E-53	113
NNU_28111	2793	NNU_023931	3240	1.00	33	6.16E-09	34
NNU_28111	2793	NNU_011227	488	0.95	91	7.69E-33	77
NNU_28113	3589	NNU_017785	5079	1.00	37	4.73E-11	38
NNU_28115	2324	NNU_007818	2578	0.87	87	5.04E-19	52
NNU_28117	194	NNU_002388	2638	0.93	41	1.37E-09	33
NNU_28122	725	NNU_025468	582	0.96	241	1.75E-108	212
NNU_28127	4519	NNU_025692	959	0.86	206	5.60E-56	119
NNU_28127	4519	NNU_025693	629	0.88	120	1.25E-32	77
NNU_28130	601	NNU_018887	645	0.83	520	6.51E-127	245
NNU_28131	1192	NNU_022416	381	0.92	218	3.02E-83	167
NNU_28131	1192	NNU_011765	489	0.94	110	6.93E-40	89
NNU_28131	1192	NNU_005681	1575	0.97	33	1.21E-07	31
NNU_28133	2385	NNU_025754	2512	0.84	1006	0	529
NNU_28133	2385	NNU_003938	1461	0.83	366	1.31E-84	170
NNU_28133	2385	NNU_003632	1976	0.84	116	1.85E-23	60
NNU_28137	2269	NNU_008151	408	0.86	406	4.24E-124	241
NNU_28137	2269	NNU_007637	1155	0.88	150	4.75E-44	97
NNU_28138	2020	NNU_009276	2774	0.93	552	0	422
NNU_28139	915	NNU_022627	857	0.89	71	3.27E-17	48
NNU_28142	2521	NNU_012577	1437	0.91	67	7.08E-18	50

NNU_28144	1701	NNU_012560	483	0.77	459	3.45E-64	133
NNU_28145	518	NNU_008051	3098	0.91	86	8.33E-26	63
NNU_28145	518	NNU_023559	1557	0.86	101	1.39E-23	59
NNU_28148	4026	NNU_015695	606	0.91	120	8.52E-39	88
NNU_28154	1788	NNU_002032	1457	0.89	575	0	382
NNU_28154	1788	NNU_020669	420	0.90	370	1.96E-136	263
NNU_28154	1788	NNU_013949	2878	0.92	294	3.38E-114	223
NNU_28154	1788	NNU_023037	570	0.90	290	2.65E-105	207
NNU_28154	1788	NNU_009278	573	0.89	273	1.62E-92	184
NNU_28154	1788	NNU_025174	1496	0.94	87	8.19E-31	73
NNU_28155	1143	NNU_016509	1670	0.87	301	6.14E-95	188
NNU_28155	1143	NNU_022866	1822	0.91	125	5.13E-41	91
NNU_28158	1790	NNU_007881	1192	0.90	800	0	561
NNU_28161	258	NNU_025710	3204	0.92	219	1.31E-85	170
NNU_28161	258	NNU_021271	1723	0.90	219	2.86E-77	155
NNU_28161	258	NNU_021256	1536	0.87	219	2.90E-67	137
NNU_28163	1228	NNU_011501	6083	0.95	130	4.21E-52	111
NNU_28165	3565	NNU_009814	1262	0.89	75	7.76E-19	52
NNU_28166	3623	NNU_022017	3736	0.81	1263	0	533
NNU_28168	433	NNU_011558	985	0.98	94	2.43E-40	89
NNU_28170	2810	NNU_002301	2170	0.90	546	0	381
NNU_28170	2810	NNU_010719	967	0.89	457	2.99E-161	308
NNU_28170	2810	NNU_001395	1147	0.88	457	3.02E-156	299
NNU_28170	2810	NNU_016111	568	0.89	177	2.69E-57	121
NNU_28170	2810	NNU_012743	222	0.88	163	5.85E-49	106
NNU_28170	2810	NNU_002303	1584	0.90	123	5.94E-39	88
NNU_28170	2810	NNU_014091	2319	0.83	73	4.79E-10	36
NNU_28170	2810	NNU_014090	4357	0.83	73	4.79E-10	36
NNU_28171	1512	NNU_002357	3025	0.95	455	0	384
NNU_28171	1512	NNU_001395	1147	0.95	58	4.22E-18	50
NNU_28174	1071	NNU_001928	3259	0.92	258	7.38E-99	195
NNU_28176	766	NNU_012589	1763	0.97	34	2.13E-08	32
NNU_28178	755	NNU_007001	2352	0.82	443	1.11E-100	198
NNU_28180	2062	NNU_015858	2227	0.95	489	0	412
NNU_28180	2062	NNU_001142	2459	0.97	274	4.95E-128	248
NNU_28180	2062	NNU_014002	1506	0.97	38	3.50E-10	36
NNU_28181	4408	NNU_014619	558	0.97	32	1.63E-06	30
NNU_28181	4408	NNU_001444	744	0.95	146	3.26E-58	123
NNU_28181	4408	NNU_021045	297	0.98	46	2.69E-14	44
NNU_28181	4408	NNU_004544	413	0.92	211	5.27E-81	164
NNU_28181	4408	NNU_018119	422	1.00	30	4.53E-07	31
NNU_28181	4408	NNU_021363	282	0.95	75	2.05E-25	64
NNU_28181	4408	NNU_010144	437	0.87	251	5.31E-76	155
NNU_28181	4408	NNU_021126	758	0.91	212	6.87E-75	153

NNU_28181	4408	NNU_018454	774	0.84	290	8.89E-74	151
NNU_28181	4408	NNU_009701	318	0.83	87	3.48E-13	42
NNU_28181	4408	NNU_017240	300	0.96	79	2.63E-29	71
NNU_28181	4408	NNU_007191	336	0.88	218	1.16E-67	140
NNU_28181	4408	NNU_018474	305	0.94	171	4.16E-67	139
NNU_28181	4408	NNU_005319	467	0.91	151	1.18E-52	113
NNU_28181	4408	NNU_018147	318	0.87	225	5.39E-66	137
NNU_28181	4408	NNU_003286	414	0.92	171	9.01E-64	133
NNU_28181	4408	NNU_016434	301	0.92	165	1.95E-60	127
NNU_28181	4408	NNU_017939	301	0.87	210	2.52E-59	125
NNU_28181	4408	NNU_000606	414	1.00	30	4.53E-07	31
NNU_28181	4408	NNU_012332	615	0.84	157	3.38E-33	78
NNU_28181	4408	NNU_023807	374	0.85	214	5.50E-51	110
NNU_28181	4408	NNU_019355	1881	0.88	164	3.31E-48	105
NNU_28181	4408	NNU_019020	344	0.93	44	7.53E-10	36
NNU_28181	4408	NNU_004540	309	0.91	146	4.28E-47	103
NNU_28181	4408	NNU_022647	499	0.92	134	4.28E-47	103
NNU_28181	4408	NNU_018925	283	0.90	134	3.33E-43	96
NNU_28181	4408	NNU_026495	467	0.93	83	1.58E-26	66
NNU_28181	4408	NNU_017098	386	0.89	137	5.58E-41	92
NNU_28181	4408	NNU_015186	287	0.88	134	1.21E-37	86
NNU_28181	4408	NNU_025460	279	0.87	142	1.21E-37	86
NNU_28181	4408	NNU_018992	459	0.88	129	7.27E-35	81
NNU_28181	4408	NNU_018477	960	0.92	100	1.22E-32	77
NNU_28181	4408	NNU_016727	410	0.78	101	4.53E-07	31
NNU_28181	4408	NNU_001633	924	0.86	112	2.05E-25	64
NNU_28181	4408	NNU_015825	482	0.87	94	1.23E-22	59
NNU_28181	4408	NNU_023930	743	0.86	101	1.23E-22	59
NNU_28181	4408	NNU_018463	180	0.85	104	4.44E-22	58
NNU_28181	4408	NNU_021315	669	0.96	51	2.08E-15	46
NNU_28181	4408	NNU_016433	405	0.96	50	7.48E-15	45
NNU_28181	4408	NNU_019582	441	0.98	44	3.48E-13	42
NNU_28181	4408	NNU_017938	277	0.90	57	4.50E-12	40
NNU_28181	4408	NNU_007518	391	1.00	29	1.63E-06	30
NNU_28182	516	NNU_024350	645	0.93	118	1.35E-43	95
NNU_28184	2093	NNU_014577	3299	0.92	69	1.63E-18	51
NNU_28186	1299	NNU_001740	614	0.84	350	4.21E-92	183
NNU_28187	499	NNU_020801	886	0.86	317	3.38E-94	186
NNU_28187	499	NNU_020197	867	0.87	302	3.38E-94	186
NNU_28188	1579	NNU_001348	1875	0.96	296	2.24E-135	261
NNU_28188	1579	NNU_001358	1302	0.96	433	0	386
NNU_28189	842	NNU_001348	1875	1.00	83	2.92E-37	84
NNU_28189	842	NNU_001358	1302	1.00	79	4.88E-35	80
NNU_28189	842	NNU_001357	351	0.95	40	5.06E-10	35

NNU_28191	853	NNU_010721	706	0.98	90	8.22E-38	85
NNU_28192	3077	NNU_000581	2976	0.84	403	1.64E-109	215
NNU_28195	1705	NNU_008916	895	0.94	93	6.04E-32	75
NNU_28195	1705	NNU_012589	1763	0.97	34	4.83E-08	32
NNU_28198	4299	NNU_007097	1101	0.98	50	1.57E-16	48
NNU_28199	906	NNU_007082	3790	0.92	156	8.50E-58	121
NNU_28200	2601	NNU_007078	783	0.92	61	3.40E-16	47
NNU_28201	820	NNU_001548	1137	1.00	91	1.01E-41	92
NNU_28208	2784	NNU_014303	2672	0.94	47	1.02E-11	39
NNU_28212	3086	NNU_023985	2509	0.95	150	1.36E-60	127
NNU_28212	3086	NNU_016314	519	0.93	122	5.01E-45	99
NNU_28212	3086	NNU_010154	4600	0.89	128	3.93E-36	83
NNU_28215	748	NNU_007969	266	0.93	54	1.60E-14	43
NNU_28218	1883	NNU_019470	6016	0.80	280	3.88E-54	115
NNU_28218	1883	NNU_020149	2414	0.79	179	2.42E-26	65
NNU_28219	763	NNU_003545	1059	0.91	408	2.22E-157	300
NNU_28220	1794	NNU_000448	806	0.89	147	1.35E-43	96
NNU_28223	8451	NNU_019536	2530	0.94	204	7.83E-82	166
NNU_28223	8451	NNU_019537	625	0.97	108	3.83E-45	100
NNU_28223	8451	NNU_005823	4719	0.86	176	2.96E-46	102
NNU_28224	947	NNU_026248	606	0.84	172	2.55E-38	86
NNU_28227	1276	NNU_021293	1422	0.89	130	7.42E-40	89
NNU_28227	1276	NNU_009102	507	0.90	88	2.11E-25	63
NNU_28231	989	NNU_002195	740	0.95	289	5.07E-125	242
NNU_28231	989	NNU_006673	912	0.96	79	5.80E-30	71
NNU_28232	2189	NNU_013333	4081	0.89	109	2.80E-31	74
NNU_28236	894	NNU_001672	1422	0.89	220	1.06E-71	146
NNU_28237	2364	NNU_025560	1677	1.00	75	2.34E-32	76
NNU_28238	992	NNU_011756	518	0.83	166	5.78E-35	80
NNU_28240	1258	NNU_016696	2793	0.95	467	0	398
NNU_28244	1171	NNU_021089	1348	0.96	512	0	455
NNU_28244	1171	NNU_022786	1350	0.92	128	1.88E-45	99
NNU_28244	1171	NNU_011377	1803	0.97	78	5.33E-31	73
NNU_28244	1171	NNU_015876	3162	0.96	78	2.48E-29	70
NNU_28248	349	NNU_023677	1617	0.86	199	2.44E-54	114
NNU_28253	416	NNU_015379	2005	0.98	42	4.04E-13	40
NNU_28253	416	NNU_009912	270	0.92	83	6.61E-26	63
NNU_28253	416	NNU_019615	2404	0.93	66	8.61E-20	52
NNU_28253	416	NNU_022191	213	0.94	48	4.04E-13	40
NNU_28256	902	NNU_012878	657	0.92	578	0	427
NNU_28256	902	NNU_007900	979	0.93	341	2.70E-142	273
NNU_28256	902	NNU_012877	219	0.98	51	8.96E-18	49
NNU_28259	7902	NNU_018289	735	0.88	142	1.30E-39	90
NNU_28259	7902	NNU_002414	2537	0.90	90	3.68E-25	64

NNU_28259	7902	NNU_008019	3229	0.81	147	7.97E-22	58
NNU_28259	7902	NNU_026405	1252	1.00	36	3.76E-10	37
NNU_28261	541	NNU_018278	1653	0.98	56	8.78E-21	54
NNU_28262	4351	NNU_022030	762	0.93	71	1.58E-21	57
NNU_28262	4351	NNU_021513	600	0.92	65	3.41E-18	51
NNU_28266	5724	NNU_000255	1650	0.88	83	3.47E-19	53
NNU_28266	5724	NNU_007381	1297	0.84	100	4.49E-18	51
NNU_28266	5724	NNU_012705	3171	0.95	58	1.62E-17	50
NNU_28266	5724	NNU_004409	1540	0.87	88	5.81E-17	49
NNU_28266	5724	NNU_019035	2257	1.00	37	7.57E-11	38
NNU_28266	5724	NNU_004207	865	0.93	41	4.56E-08	33
NNU_28270	3318	NNU_009850	1861	1.00	32	2.63E-08	33
NNU_28275	1254	NNU_006940	4006	0.78	511	1.49E-76	155
NNU_28275	1254	NNU_001260	1102	1.00	33	2.74E-09	34
NNU_28276	1646	NNU_005533	3858	0.95	159	7.17E-66	136
NNU_28277	868	NNU_005748	1009	0.97	127	4.90E-55	116
NNU_28280	3102	NNU_002727	2707	0.96	70	1.86E-24	62
NNU_28280	3102	NNU_001988	853	0.87	208	8.19E-63	131
NNU_28283	757	NNU_003487	1908	0.85	285	8.95E-72	146
NNU_28283	757	NNU_011453	846	0.84	122	2.65E-27	66
NNU_28284	2163	NNU_011378	1380	0.92	1354	0	1045
NNU_28284	2163	NNU_000536	483	0.91	472	0	350
NNU_28285	3730	NNU_011381	381	0.88	319	2.59E-103	204
NNU_28285	3730	NNU_006709	3256	0.90	131	1.70E-40	91
NNU_28285	3730	NNU_024899	3722	0.93	67	2.26E-19	53
NNU_28286	3190	NNU_011393	4349	0.96	484	0	424
NNU_28287	7026	NNU_004737	1642	0.88	88	9.17E-21	56
NNU_28292	1850	NNU_020118	3498	0.89	86	6.65E-22	57
NNU_28294	169	NNU_026065	899	0.88	136	4.04E-39	86
NNU_28309	3239	NNU_019568	10194	0.81	631	2.77E-137	265
NNU_28315	1247	NNU_007902	1695	0.96	97	1.21E-37	85
NNU_28315	1247	NNU_025173	3253	0.93	90	5.69E-31	73
NNU_28315	1247	NNU_020668	4831	0.83	107	1.25E-17	49
NNU_28315	1247	NNU_006219	2926	0.96	44	4.52E-12	39
NNU_28317	2819	NNU_022004	2094	0.90	99	2.17E-28	69
NNU_28325	4252	NNU_018016	1017	0.93	215	2.35E-84	170
NNU_28329	901	NNU_000416	3802	0.91	284	2.19E-108	212
NNU_28338	1516	NNU_016611	2724	0.91	96	3.22E-29	70
NNU_28339	934	NNU_020284	291	0.88	136	1.50E-40	90
NNU_28339	934	NNU_014409	291	0.88	136	2.51E-38	86
NNU_28339	934	NNU_004564	189	0.90	110	2.53E-33	77
NNU_28346	930	NNU_017731	1042	0.83	307	5.11E-75	152
NNU_28346	930	NNU_017733	1061	0.86	188	1.14E-51	110
NNU_28346	930	NNU_026580	243	0.99	78	9.05E-33	76

NNU_28348	1305	NNU_005722	2399	0.96	626	0	552
NNU_28348	1305	NNU_005592	1156	0.97	67	7.76E-25	62
NNU_28349	646	NNU_009567	1884	0.99	108	1.31E-49	106
NNU_28349	646	NNU_007775	3381	0.96	72	2.91E-26	64
NNU_28349	646	NNU_003501	2994	0.98	43	1.78E-13	41
NNU_28349	646	NNU_005963	2744	0.95	43	8.26E-12	38
NNU_28350	1899	NNU_008440	3497	0.89	119	5.20E-33	77
NNU_28350	1899	NNU_004498	794	0.88	111	1.13E-29	71
NNU_28350	1899	NNU_005032	3027	0.86	105	1.47E-23	60
NNU_28353	2245	NNU_023606	1245	0.84	230	9.96E-56	118
NNU_28354	1240	NNU_012449	2365	0.84	303	1.47E-76	155
NNU_28357	937	NNU_019486	779	0.89	124	1.17E-36	83
NNU_28357	937	NNU_001141	1191	0.94	63	2.00E-19	52
NNU_28357	937	NNU_012765	3480	0.89	64	5.61E-15	44
NNU_28361	1456	NNU_010270	502	0.90	105	2.39E-30	72
NNU_28361	1456	NNU_008738	495	0.83	119	5.21E-22	57
NNU_28361	1456	NNU_012888	2064	0.93	45	6.84E-11	37
NNU_28364	2005	NNU_002773	1383	0.97	191	1.41E-88	177
NNU_28364	2005	NNU_022786	1350	0.84	149	1.53E-33	78
NNU_28366	756	NNU_002333	1491	0.91	116	5.61E-39	87
NNU_28366	756	NNU_021022	1485	0.88	116	2.63E-32	75
NNU_28371	582	NNU_000763	3023	0.90	560	0	393
NNU_28371	582	NNU_021051	1449	0.97	30	2.69E-06	28
NNU_28377	3052	NNU_012433	2810	0.98	41	1.12E-11	39
NNU_28377	3052	NNU_010334	3857	0.93	39	3.13E-07	31
NNU_28380	1740	NNU_004125	1905	0.81	1002	0	422
NNU_28380	1740	NNU_025547	831	0.94	376	1.11E-158	303
NNU_28380	1740	NNU_002001	618	0.85	440	7.02E-121	235
NNU_28380	1740	NNU_013784	600	0.95	215	4.38E-93	185
NNU_28380	1740	NNU_023177	1257	0.91	142	1.68E-47	103
NNU_28382	9652	NNU_009876	669	0.82	375	8.95E-82	166
NNU_28382	9652	NNU_003878	4985	0.86	119	3.48E-26	66
NNU_28389	3035	NNU_003361	1587	0.91	363	2.59E-137	265
NNU_28389	3035	NNU_001291	1866	0.87	103	6.56E-24	61
NNU_28389	3035	NNU_008902	1689	0.86	139	1.39E-35	82
NNU_28389	3035	NNU_007415	837	0.89	230	3.65E-76	155
NNU_28389	3035	NNU_022459	1113	0.90	116	1.80E-34	80
NNU_28389	3035	NNU_003871	297	0.98	49	3.97E-16	47
NNU_28394	10759	NNU_024722	644	0.89	508	0	347
NNU_28394	10759	NNU_026321	2270	0.90	244	1.28E-85	173
NNU_28394	10759	NNU_013276	2499	0.84	277	6.09E-69	143
NNU_28394	10759	NNU_002318	3126	0.85	176	1.75E-44	99
NNU_28394	10759	NNU_002306	1530	0.84	176	3.80E-41	93
NNU_28394	10759	NNU_015383	2314	0.79	259	1.06E-36	85

NNU_28394	10759	NNU_007381	1297	0.85	153	6.40E-34	80
NNU_28394	10759	NNU_000671	2394	0.83	129	1.80E-24	63
NNU_28394	10759	NNU_019144	2645	0.84	112	1.09E-21	58
NNU_28397	1067	NNU_006690	2930	0.95	470	0	403
NNU_28397	1067	NNU_022623	2679	0.85	221	4.68E-56	118
NNU_28397	1067	NNU_022620	3298	0.77	167	8.23E-19	51
NNU_28399	2209	NNU_015647	939	0.84	956	0	495
NNU_28399	2209	NNU_004368	5914	0.93	269	3.26E-110	216
NNU_28399	2209	NNU_013312	857	0.92	220	5.65E-83	167
NNU_28401	2111	NNU_014063	843	0.90	264	4.11E-94	187
NNU_28401	2111	NNU_000652	471	0.92	132	2.04E-47	103
NNU_28402	716	NNU_015777	1373	0.96	47	5.50E-14	42
NNU_28403	841	NNU_008146	846	0.87	188	1.72E-49	106
NNU_28403	841	NNU_025919	2427	0.94	223	7.50E-93	184
NNU_28403	841	NNU_009099	1218	0.93	127	1.03E-46	101
NNU_28404	556	NNU_005791	411	0.96	76	1.49E-28	68
NNU_28405	3682	NNU_010471	499	0.84	339	1.56E-90	181
NNU_28405	3682	NNU_014041	788	0.98	39	1.75E-10	37
NNU_28405	3682	NNU_001397	1838	0.82	329	1.25E-66	138
NNU_28405	3682	NNU_023930	743	0.81	298	1.64E-55	118
NNU_28405	3682	NNU_004195	3693	0.91	90	3.68E-27	67
NNU_28405	3682	NNU_024129	1491	0.94	53	6.24E-15	45
NNU_28405	3682	NNU_010851	1272	0.86	56	2.92E-08	33
NNU_28407	2521	NNU_004250	3927	0.96	255	2.21E-117	229
NNU_28407	2521	NNU_018754	5368	0.90	225	8.42E-77	156
NNU_28409	1997	NNU_023020	2024	0.91	453	3.47E-174	331
NNU_28409	1997	NNU_010736	2063	0.77	349	8.91E-51	109
NNU_28412	2938	NNU_023397	1842	0.99	97	7.98E-43	95
NNU_28412	2938	NNU_013934	2234	0.94	102	2.89E-37	85
NNU_28416	3294	NNU_011702	1845	0.92	60	1.55E-15	46
NNU_28419	2439	NNU_004250	3927	0.97	271	2.73E-126	245
NNU_28419	2439	NNU_018754	5368	0.90	155	3.03E-51	110
NNU_28420	1451	NNU_002301	2170	0.87	766	0	477
NNU_28420	1451	NNU_002303	1584	0.89	285	1.31E-92	184
NNU_28420	1451	NNU_011620	2976	0.75	470	6.40E-56	118
NNU_28420	1451	NNU_001398	696	0.93	60	5.23E-17	48
NNU_28420	1451	NNU_001395	1147	0.90	161	3.85E-53	113
NNU_28420	1451	NNU_010719	967	0.89	161	1.79E-51	110
NNU_28420	1451	NNU_012743	222	0.88	158	8.39E-45	98
NNU_28420	1451	NNU_026274	1977	0.78	258	1.41E-37	85
NNU_28420	1451	NNU_002302	237	0.94	77	2.40E-25	63
NNU_28421	468	NNU_005641	679	0.92	105	9.55E-35	79
NNU_28422	4058	NNU_000465	925	0.88	465	9.45E-153	293
NNU_28427	4253	NNU_001397	1838	0.80	315	1.13E-57	122

NNU_28427	4253	NNU_023248	3440	0.90	149	1.15E-47	104
NNU_28427	4253	NNU_022014	1998	0.88	149	1.16E-42	95
NNU_28427	4253	NNU_000195	1494	1.00	28	5.66E-06	29
NNU_28428	899	NNU_001397	1838	0.87	241	8.26E-73	148
NNU_28428	899	NNU_010471	499	0.92	48	1.16E-11	38
NNU_28428	899	NNU_014041	788	0.97	33	9.06E-08	31
NNU_28429	1062	NNU_005576	1991	0.91	463	3.96E-171	325
NNU_28429	1062	NNU_009557	3129	0.87	226	3.55E-67	138
NNU_28437	759	NNU_022262	3001	0.91	218	1.48E-79	160
NNU_28437	759	NNU_018527	480	0.92	135	7.19E-48	103
NNU_28437	759	NNU_008638	485	0.92	98	2.64E-32	75
NNU_28437	759	NNU_012157	441	0.89	100	2.06E-28	68
NNU_28442	5826	NNU_003217	2653	0.95	77	2.10E-26	66
NNU_28442	5826	NNU_026673	1164	0.95	196	3.22E-84	170
NNU_28442	5826	NNU_001964	1086	0.95	72	1.26E-23	61
NNU_28442	5826	NNU_024084	408	0.96	65	2.11E-21	57
NNU_28442	5826	NNU_015449	546	0.92	71	9.83E-20	54
NNU_28442	5826	NNU_015614	2541	0.98	48	2.75E-15	46
NNU_28442	5826	NNU_025749	3272	0.95	40	3.59E-09	35
NNU_28443	819	NNU_026285	2207	0.91	693	0	500
NNU_28443	819	NNU_026287	1449	0.89	746	0	499
NNU_28443	819	NNU_023680	1719	1.00	55	1.04E-21	56
NNU_28445	3172	NNU_010133	3573	0.87	527	1.56E-164	314
NNU_28445	3172	NNU_009171	3678	0.80	456	2.26E-83	168
NNU_28445	3172	NNU_001346	441	0.90	220	1.06E-76	156
NNU_28445	3172	NNU_013984	576	0.92	124	8.62E-43	95
NNU_28445	3172	NNU_017061	3351	0.95	40	1.95E-09	35
NNU_28445	3172	NNU_021337	3929	0.97	31	4.21E-06	29
NNU_28446	1233	NNU_020955	1000	0.92	744	0	558
NNU_28447	7315	NNU_019395	4780	0.96	89	5.63E-33	78
NNU_28447	7315	NNU_002814	2832	0.86	130	2.02E-32	77
NNU_28447	7315	NNU_000105	8491	0.88	95	5.71E-23	60
NNU_28450	7120	NNU_011023	1303	0.81	148	2.00E-22	59
NNU_28454	4453	NNU_008441	1168	0.97	99	2.03E-40	91
NNU_28454	4453	NNU_011232	593	0.96	100	2.62E-39	89
NNU_28454	4453	NNU_008482	462	0.96	44	1.64E-11	39
NNU_28454	4453	NNU_003464	1536	0.86	83	1.26E-17	50
NNU_28454	4453	NNU_019075	3355	0.91	67	4.52E-17	49
NNU_28454	4453	NNU_016634	48	0.98	47	7.56E-15	45
NNU_28455	3554	NNU_013385	5210	0.91	100	4.56E-31	74
NNU_28455	3554	NNU_016012	2716	0.84	200	2.67E-48	105
NNU_28456	1640	NNU_013369	4150	0.94	338	4.93E-147	282
NNU_28456	1640	NNU_011118	3176	0.95	41	7.72E-11	37
NNU_28458	6027	NNU_011691	716	0.89	352	4.08E-123	240

NNU_28458	6027	NNU_016810	1833	0.91	151	2.10E-51	111
NNU_28458	6027	NNU_015720	1528	0.92	193	1.57E-72	149
NNU_28458	6027	NNU_000617	611	0.85	191	7.59E-46	101
NNU_28458	6027	NNU_000616	1798	0.88	153	9.82E-45	99
NNU_28458	6027	NNU_018096	330	0.92	102	1.29E-33	79
NNU_28458	6027	NNU_021229	863	0.93	87	4.67E-28	69
NNU_28458	6027	NNU_018498	1528	0.93	72	2.19E-21	57
NNU_28459	3317	NNU_000105	8491	0.97	32	1.22E-06	30
NNU_28459	3317	NNU_026179	931	0.87	168	5.38E-45	99
NNU_28459	3317	NNU_023123	1803	0.83	171	1.17E-36	84
NNU_28459	3317	NNU_018179	2813	0.81	151	1.54E-25	64
NNU_28459	3317	NNU_018192	1620	0.91	85	1.99E-24	62
NNU_28459	3317	NNU_001712	2005	0.88	80	2.59E-18	51
NNU_28459	3317	NNU_022853	2276	0.97	31	4.40E-06	29
NNU_28461	1155	NNU_002499	2448	0.91	207	8.26E-74	150
NNU_28462	5585	NNU_000455	1143	0.90	458	7.62E-170	324
NNU_28462	5585	NNU_003153	2814	0.97	265	6.28E-126	245
NNU_28462	5585	NNU_023878	807	0.90	208	2.44E-70	145
NNU_28462	5585	NNU_016785	1215	0.97	108	2.53E-45	100
NNU_28462	5585	NNU_000069	897	0.86	129	5.55E-32	76
NNU_28462	5585	NNU_013776	432	0.95	60	1.22E-18	52
NNU_28462	5585	NNU_011994	1295	0.89	69	2.64E-15	46
NNU_28462	5585	NNU_010747	1522	0.91	63	2.64E-15	46
NNU_28462	5585	NNU_005327	2341	1.00	28	7.44E-06	29
NNU_28464	1449	NNU_002566	458	0.79	318	1.38E-52	112
NNU_28464	1449	NNU_005558	2975	0.91	145	1.08E-48	105
NNU_28464	1449	NNU_013492	2276	0.91	132	1.08E-43	96
NNU_28464	1449	NNU_003537	1734	0.92	101	3.06E-34	79
NNU_28464	1449	NNU_019808	1393	0.92	78	3.10E-24	61
NNU_28464	1449	NNU_009177	924	0.88	72	6.76E-16	46
NNU_28464	1449	NNU_012659	2863	0.92	48	6.81E-11	37
NNU_28464	1449	NNU_015961	1861	0.95	39	3.17E-09	34
NNU_28464	1449	NNU_008327	3038	1.00	27	6.86E-06	28
NNU_28468	2038	NNU_001695	4395	0.96	1575	0	1379
NNU_28470	2425	NNU_000428	1323	0.86	468	3.41E-145	279
NNU_28472	2210	NNU_021419	3880	0.92	211	2.05E-77	157
NNU_28472	2210	NNU_000559	1791	0.91	55	6.24E-13	41
NNU_28472	2210	NNU_013044	1126	1.00	37	2.90E-11	38
NNU_28472	2210	NNU_001338	702	0.95	40	1.35E-09	35
NNU_28474	2430	NNU_022765	1175	0.88	274	6.18E-88	176
NNU_28475	880	NNU_002755	2085	0.87	62	5.29E-10	35
NNU_28476	3710	NNU_014851	3748	0.86	280	5.73E-80	162
NNU_28476	3710	NNU_000278	3486	0.96	46	1.05E-12	41
NNU_28481	1502	NNU_012072	1213	0.93	368	5.79E-151	289

NNU_28481	1502	NNU_005247	1065	0.93	200	4.98E-77	156
NNU_28481	1502	NNU_006726	543	0.88	95	1.16E-23	60
NNU_28485	865	NNU_000455	1143	0.90	456	1.49E-169	322
NNU_28485	865	NNU_023878	807	0.89	206	4.78E-70	143
NNU_28485	865	NNU_016785	1215	0.97	108	3.83E-46	100
NNU_28485	865	NNU_000069	897	0.86	129	8.40E-33	76
NNU_28485	865	NNU_003153	2814	0.98	62	6.59E-24	60
NNU_28488	3291	NNU_011458	1326	0.89	147	1.48E-45	100
NNU_28492	1429	NNU_022777	1804	0.94	63	3.08E-19	52
NNU_28493	914	NNU_001674	2038	0.97	37	5.50E-10	35
NNU_28496	859	NNU_001056	3362	0.98	41	3.09E-12	39
NNU_28496	859	NNU_002653	2707	0.94	47	1.11E-11	38
NNU_28501	847	NNU_014257	363	0.90	76	1.40E-20	54
NNU_28502	1049	NNU_026092	718	0.87	684	0	425
NNU_28502	1049	NNU_020337	1344	0.86	280	3.43E-82	165
NNU_28502	1049	NNU_024154	531	0.93	116	1.31E-41	92
NNU_28506	769	NNU_017502	4259	0.91	112	3.44E-36	82
NNU_28508	824	NNU_026335	1878	0.85	790	0	412
NNU_28509	2044	NNU_006984	1329	0.85	298	8.74E-81	163
NNU_28509	2044	NNU_006985	2443	0.93	122	3.30E-45	99
NNU_28509	2044	NNU_021438	3246	0.83	104	1.59E-18	51
NNU_28509	2044	NNU_020407	4104	1.00	28	2.70E-06	29
NNU_28510	959	NNU_001530	990	0.93	702	0	553
NNU_28510	959	NNU_023482	3228	0.91	665	0	491
NNU_28510	959	NNU_006660	432	0.92	377	7.88E-153	292
NNU_28510	959	NNU_000051	303	0.94	303	2.94E-127	246
NNU_28510	959	NNU_000052	456	0.95	206	8.64E-88	175
NNU_28510	959	NNU_007653	447	0.93	72	9.47E-23	58
NNU_28514	1566	NNU_003151	2354	0.94	198	6.67E-81	163
NNU_28514	1566	NNU_010006	2060	0.91	178	1.48E-62	130
NNU_28514	1566	NNU_003156	2439	0.88	197	3.19E-59	124
NNU_28520	2620	NNU_019366	2871	0.76	984	8.15E-127	246
NNU_28522	2964	NNU_024406	2545	0.81	478	1.59E-104	206
NNU_28524	210	NNU_026026	2658	0.84	181	2.39E-42	92
NNU_28528	3332	NNU_021169	1709	0.95	446	0	376
NNU_28528	3332	NNU_006830	1020	0.88	446	6.03E-149	286
NNU_28528	3332	NNU_025056	572	0.90	392	1.02E-141	273
NNU_28528	3332	NNU_024562	614	0.90	392	1.70E-139	269
NNU_28528	3332	NNU_004854	2502	0.91	332	2.25E-123	240
NNU_28528	3332	NNU_017436	470	0.89	350	3.76E-121	236
NNU_28528	3332	NNU_014922	1433	0.88	165	5.41E-45	99
NNU_28528	3332	NNU_007352	2396	0.92	119	1.51E-40	91
NNU_28529	2295	NNU_007952	1029	0.85	1004	0	543
NNU_28531	1166	NNU_001865	2929	0.93	654	0	526

NNU_28532	2737	NNU_016842	4408	0.94	61	7.69E-18	50
NNU_28532	2737	NNU_015993	3325	0.84	110	1.65E-19	53
NNU_28532	2737	NNU_016507	1238	0.85	88	3.58E-16	47
NNU_28532	2737	NNU_005327	2341	0.80	120	2.79E-12	40
NNU_28532	2737	NNU_005729	1706	0.92	77	2.12E-23	60
NNU_28532	2737	NNU_004797	1906	0.84	101	2.77E-17	49
NNU_28532	2737	NNU_001653	1563	0.98	40	3.61E-11	38
NNU_28532	2737	NNU_003185	3835	0.95	59	2.14E-18	51
NNU_28532	2737	NNU_015940	766	0.83	108	9.95E-17	48
NNU_28532	2737	NNU_013492	2276	0.91	64	9.95E-17	48
NNU_28532	2737	NNU_009451	3613	1.00	28	3.63E-06	29
NNU_28532	2737	NNU_000605	1684	0.83	93	4.63E-15	45
NNU_28532	2737	NNU_019304	2730	0.91	55	7.75E-13	41
NNU_28532	2737	NNU_014878	2550	0.92	51	1.00E-11	39
NNU_28532	2737	NNU_000616	1798	1.00	37	3.61E-11	38
NNU_28532	2737	NNU_003455	2936	0.95	41	4.66E-10	36
NNU_28532	2737	NNU_014191	2828	0.93	42	6.03E-09	34
NNU_28532	2737	NNU_005621	2435	0.88	49	2.81E-07	31
NNU_28541	1643	NNU_006753	1722	0.97	276	1.40E-132	256
NNU_28541	1643	NNU_013733	3601	0.80	984	0	382
NNU_28542	1323	NNU_003455	2936	0.97	31	1.74E-06	29
NNU_28544	2455	NNU_009419	1538	0.91	103	2.43E-32	76
NNU_28546	1232	NNU_009336	1949	0.78	146	3.43E-18	50
NNU_28552	646	NNU_017077	597	0.93	94	2.23E-32	75
NNU_28557	1257	NNU_013464	350	0.96	48	2.72E-14	43
NNU_28558	5287	NNU_019074	1785	0.77	359	3.98E-48	105
NNU_28558	5287	NNU_022853	2276	0.91	97	2.44E-30	73
NNU_28558	5287	NNU_005931	2442	0.83	115	6.89E-21	56
NNU_28558	5287	NNU_010441	4874	0.78	111	9.04E-10	36
NNU_28560	717	NNU_026435	2456	0.87	256	6.54E-73	148
NNU_28565	3228	NNU_023685	2283	0.84	720	0	362
NNU_28566	1067	NNU_018729	2377	0.90	100	6.27E-30	71
NNU_28567	7870	NNU_003938	1461	0.90	351	1.14E-124	243
NNU_28567	7870	NNU_026227	994	0.83	249	2.72E-56	120
NNU_28567	7870	NNU_014041	788	0.89	165	5.88E-53	114
NNU_28567	7870	NNU_010471	499	0.84	157	2.80E-36	84
NNU_28567	7870	NNU_015956	4289	0.89	150	3.56E-45	100
NNU_28567	7870	NNU_010311	631	0.89	121	1.01E-35	83
NNU_28567	7870	NNU_007129	3240	0.79	209	2.81E-31	75
NNU_28567	7870	NNU_004346	1190	0.94	84	1.69E-28	70
NNU_28567	7870	NNU_009192	1273	0.94	82	2.19E-27	68
NNU_28567	7870	NNU_015993	3325	0.89	96	1.02E-25	65
NNU_28567	7870	NNU_011994	1295	0.94	66	3.69E-20	55
NNU_28567	7870	NNU_002511	1735	0.84	112	1.33E-19	54

NNU_28567	7870	NNU_005327	2341	0.90	72	1.72E-18	52
NNU_28567	7870	NNU_013804	5355	0.82	115	2.22E-17	50
NNU_28567	7870	NNU_003416	3965	0.86	79	3.72E-15	46
NNU_28567	7870	NNU_008550	1698	0.81	105	3.72E-15	46
NNU_28567	7870	NNU_023405	3245	0.80	122	3.72E-15	46
NNU_28567	7870	NNU_004195	3693	1.00	43	4.81E-14	44
NNU_28567	7870	NNU_024129	1491	1.00	43	4.81E-14	44
NNU_28567	7870	NNU_000024	971	0.97	36	1.74E-08	34
NNU_28568	1342	NNU_019568	10194	0.87	52	3.79E-08	32
NNU_28569	2483	NNU_005364	2328	0.96	238	1.03E-105	208
NNU_28569	2483	NNU_014622	1068	0.86	267	6.41E-78	158
NNU_28569	2483	NNU_004777	2187	0.85	237	2.35E-62	130
NNU_28569	2483	NNU_000654	354	0.98	88	1.13E-35	82
NNU_28569	2483	NNU_009472	1173	0.98	39	1.18E-10	37
NNU_28570	274	NNU_013603	2154	0.95	146	1.87E-59	123
NNU_28572	930	NNU_011002	300	0.98	50	3.33E-17	48
NNU_28572	930	NNU_024428	429	0.91	65	3.33E-17	48
NNU_28572	930	NNU_018134	558	0.83	87	2.00E-14	43
NNU_28577	3463	NNU_006105	2786	0.83	165	7.38E-34	79
NNU_28577	3463	NNU_013010	2008	0.90	72	7.53E-19	52
NNU_28579	671	NNU_014840	1005	0.94	126	6.33E-48	103
NNU_28579	671	NNU_020839	1449	0.89	401	5.57E-138	265
NNU_28580	2283	NNU_005364	2328	0.96	238	9.44E-106	208
NNU_28580	2283	NNU_004777	2187	0.85	237	2.16E-62	130
NNU_28580	2283	NNU_014622	1068	0.90	147	7.94E-47	102
NNU_28580	2283	NNU_000654	354	0.98	88	1.04E-35	82
NNU_28580	2283	NNU_009472	1173	0.98	39	1.08E-10	37
NNU_28585	904	NNU_007068	2041	0.93	72	8.92E-23	58
NNU_28589	1146	NNU_014711	5268	0.84	166	6.65E-40	89
NNU_28603	741	NNU_008796	1347	0.90	80	7.26E-23	58
NNU_28605	1282	NNU_018787	1068	0.96	52	5.97E-16	46
NNU_28607	1278	NNU_008214	1188	0.77	1034	2.26E-159	304
NNU_28610	635	NNU_013662	393	0.94	160	3.49E-65	134
NNU_28613	967	NNU_002335	2572	0.96	147	6.98E-64	132
NNU_28613	967	NNU_011026	588	0.95	147	1.51E-60	126
NNU_28613	967	NNU_013103	320	0.94	145	9.09E-58	121
NNU_28616	659	NNU_011677	240	0.95	212	2.09E-92	183
NNU_28616	659	NNU_010366	3723	0.88	84	2.99E-21	55
NNU_28616	659	NNU_015892	4195	1.00	44	1.08E-15	45
NNU_28618	903	NNU_021231	543	0.86	266	3.83E-76	154
NNU_28618	903	NNU_016970	1221	1.00	86	6.74E-39	87
NNU_28618	903	NNU_000784	492	0.97	37	5.44E-10	35
NNU_28620	1138	NNU_020705	4766	0.89	69	1.90E-15	45
NNU_28622	1661	NNU_020874	1487	0.85	449	6.65E-126	244

NNU_28622	1661	NNU_026227	994	0.82	396	3.25E-89	178
NNU_28622	1661	NNU_012322	549	0.87	262	5.51E-77	156
NNU_28622	1661	NNU_014707	1115	0.85	245	5.59E-67	138
NNU_28622	1661	NNU_008084	837	0.86	241	2.01E-66	137
NNU_28624	2201	NNU_005022	840	0.80	109	1.34E-14	44
NNU_28625	1355	NNU_026399	936	0.85	441	5.41E-126	244
NNU_28625	1355	NNU_018349	806	0.88	380	1.95E-125	243
NNU_28625	1355	NNU_020352	591	0.86	218	4.59E-62	129
NNU_28629	595	NNU_003367	2771	0.97	63	5.80E-23	58
NNU_28633	3444	NNU_001379	859	0.89	618	0	405
NNU_28633	3444	NNU_026226	696	0.89	455	7.95E-158	302
NNU_28633	3444	NNU_008783	1206	0.93	311	3.86E-126	245
NNU_28633	3444	NNU_001397	1838	0.83	508	6.50E-119	232
NNU_28633	3444	NNU_016446	1615	0.93	98	2.64E-33	78
NNU_28633	3444	NNU_009070	302	0.91	179	1.18E-61	129
NNU_28633	3444	NNU_016450	1756	0.93	160	1.97E-59	125
NNU_28633	3444	NNU_016447	1581	0.90	172	9.16E-58	122
NNU_28633	3444	NNU_007684	1038	0.92	108	1.58E-35	82
NNU_28633	3444	NNU_015388	612	0.91	105	3.41E-32	76
NNU_28633	3444	NNU_004195	3693	0.91	105	1.23E-31	75
NNU_28633	3444	NNU_024129	1491	0.98	53	2.69E-18	51
NNU_28633	3444	NNU_023930	743	0.89	69	1.62E-15	46
NNU_28633	3444	NNU_000688	3067	1.00	28	4.57E-06	29
NNU_28634	1450	NNU_025796	2419	0.89	163	2.32E-50	108
NNU_28635	6113	NNU_003067	4300	0.94	466	0	383
NNU_28635	6113	NNU_006430	2037	0.93	212	1.57E-82	167
NNU_28635	6113	NNU_025092	276	0.86	205	2.11E-56	120
NNU_28635	6113	NNU_019944	552	0.95	82	1.32E-28	70
NNU_28637	3671	NNU_011772	1487	0.95	182	4.42E-76	155
NNU_28637	3671	NNU_022688	2838	0.93	115	4.64E-41	92
NNU_28637	3671	NNU_020613	1272	0.95	51	2.24E-14	44
NNU_28638	1198	NNU_005574	3376	0.87	159	6.91E-45	98
NNU_28645	3350	NNU_021147	1509	0.98	65	5.59E-25	63
NNU_28646	661	NNU_016425	303	0.98	111	1.34E-49	106
NNU_28646	661	NNU_002214	1327	0.89	149	2.24E-47	102
NNU_28646	661	NNU_021147	1509	0.99	66	1.07E-25	63
NNU_28647	6480	NNU_009115	3241	0.95	286	1.22E-123	241
NNU_28647	6480	NNU_010378	5250	0.93	134	4.88E-48	105
NNU_28647	6480	NNU_014548	480	0.96	67	1.82E-22	59
NNU_28647	6480	NNU_014549	447	0.96	84	6.44E-32	76
NNU_28647	6480	NNU_019873	1204	0.84	160	2.98E-35	82
NNU_28647	6480	NNU_017130	705	1.00	39	6.63E-12	40
NNU_28647	6480	NNU_024724	626	1.00	38	2.38E-11	39
NNU_28647	6480	NNU_018956	2814	1.00	28	8.64E-06	29

NNU_28648	899	NNU_004937	2734	0.91	317	5.99E-119	231
NNU_28648	899	NNU_014046	678	0.89	319	1.02E-106	209
NNU_28648	899	NNU_010614	2304	0.95	41	1.50E-10	36
NNU_28654	545	NNU_016963	745	0.94	122	3.07E-45	98
NNU_28654	545	NNU_025024	631	0.90	106	2.43E-31	73
NNU_28656	1814	NNU_003378	1052	1.00	41	1.42E-13	42
NNU_28656	1814	NNU_019303	339	0.84	224	6.25E-52	111
NNU_28657	1821	NNU_003825	345	0.92	249	3.54E-94	187
NNU_28657	1821	NNU_016509	1670	0.92	50	6.64E-12	39
NNU_28657	1821	NNU_022866	1822	0.83	166	1.08E-29	71
NNU_28660	766	NNU_015030	2648	0.97	117	3.35E-51	109
NNU_28660	766	NNU_021722	854	0.98	49	9.79E-17	47
NNU_28660	766	NNU_021065	2644	0.94	118	4.37E-45	98
NNU_28660	766	NNU_009284	2117	1.00	33	1.65E-09	34
NNU_28660	766	NNU_012949	637	0.93	69	3.50E-21	55
NNU_28670	1924	NNU_013930	1257	0.88	57	9.08E-11	37
NNU_28672	2354	NNU_022494	1632	0.98	62	1.82E-23	60
NNU_28672	2354	NNU_013089	180	0.88	67	1.43E-14	44
NNU_28675	2442	NNU_022510	3503	0.83	222	3.93E-50	108
NNU_28675	2442	NNU_022743	5044	0.86	354	3.66E-100	198
NNU_28675	2442	NNU_018697	795	0.98	42	8.93E-12	39
NNU_28684	2659	NNU_003997	1795	0.82	204	3.36E-41	92
NNU_28685	996	NNU_017063	3077	0.93	302	2.38E-123	239
NNU_28685	996	NNU_008519	246	0.92	157	9.37E-58	121
NNU_28686	1220	NNU_024270	2380	0.93	73	3.37E-23	59
NNU_28686	1220	NNU_009057	981	0.95	123	2.51E-49	106
NNU_28686	1220	NNU_018173	2463	0.94	49	3.42E-13	41
NNU_28687	369	NNU_003981	1589	0.86	186	7.27E-50	106
NNU_28689	877	NNU_002431	1983	0.95	56	3.13E-17	48
NNU_28690	1633	NNU_012991	889	0.85	76	1.28E-13	42
NNU_28692	1762	NNU_022523	768	0.89	155	1.02E-49	107
NNU_28694	524	NNU_025632	498	0.87	442	4.28E-143	274
NNU_28694	524	NNU_004910	3684	0.88	126	1.08E-34	79
NNU_28695	1082	NNU_026563	1121	0.96	71	1.78E-25	63
NNU_28700	655	NNU_018985	300	0.91	46	3.90E-10	35
NNU_28700	655	NNU_001523	4868	0.91	46	3.90E-10	35
NNU_28700	655	NNU_006711	657	0.91	46	3.90E-10	35
NNU_28702	1777	NNU_019074	1785	0.82	407	5.86E-82	165
NNU_28708	2226	NNU_011387	183	0.96	111	1.68E-43	96
NNU_28708	2226	NNU_003539	468	0.97	101	7.80E-42	93
NNU_28708	2226	NNU_012886	1791	0.97	101	7.80E-42	93
NNU_28708	2226	NNU_014813	807	0.89	117	1.70E-33	78
NNU_28708	2226	NNU_001103	3172	0.93	97	6.11E-33	77
NNU_28712	920	NNU_024989	2483	0.96	114	4.08E-46	100

NNU_28712	920	NNU_010270	502	0.99	140	1.43E-65	135
NNU_28712	920	NNU_024277	2705	0.89	84	1.17E-21	56
NNU_28715	1068	NNU_002769	2863	0.83	138	2.26E-29	70
NNU_28719	3532	NNU_025173	3253	0.89	92	5.90E-25	63
NNU_28719	3532	NNU_006219	2926	0.94	46	1.68E-10	37
NNU_28719	3532	NNU_024732	858	1.00	28	4.69E-06	29
NNU_28721	3166	NNU_020417	430	0.94	134	3.95E-51	110
NNU_28726	592	NNU_020095	2386	0.96	78	1.23E-29	70
NNU_28726	592	NNU_021769	964	0.95	132	5.53E-53	112
NNU_28726	592	NNU_016948	1683	0.92	128	9.31E-46	99
NNU_28726	592	NNU_002607	1519	0.97	92	2.03E-37	84
NNU_28729	3458	NNU_009015	3113	0.96	53	1.26E-16	48
NNU_28730	3039	NNU_009021	3381	0.87	185	2.26E-53	114
NNU_28730	3039	NNU_001192	2961	1.00	28	4.04E-06	29
NNU_28732	2425	NNU_009072	2083	0.92	64	6.81E-18	50
NNU_28734	2344	NNU_009118	531	0.77	461	2.20E-67	139
NNU_28736	516	NNU_016711	2328	0.93	93	2.29E-31	73
NNU_28740	5231	NNU_019149	2211	1.00	28	6.97E-06	29
NNU_28741	593	NNU_020782	303	0.98	95	9.40E-41	90
NNU_28741	593	NNU_004639	651	0.95	63	2.69E-21	55
NNU_28743	789	NNU_014645	1863	0.84	137	1.28E-30	72
NNU_28743	789	NNU_007898	777	0.85	130	4.60E-30	71
NNU_28745	6384	NNU_014556	1290	0.99	157	3.59E-74	152
NNU_28745	6384	NNU_014089	362	0.92	77	4.98E-23	60
NNU_28745	6384	NNU_014560	1095	1.00	50	5.01E-18	51
NNU_28745	6384	NNU_015054	1132	1.00	38	2.35E-11	39
NNU_28746	2958	NNU_008609	1661	0.99	68	1.06E-26	66
NNU_28747	597	NNU_014041	788	0.97	194	5.31E-88	175
NNU_28747	597	NNU_012832	2403	0.97	68	9.66E-26	63
NNU_28747	597	NNU_009420	2846	0.83	193	5.65E-43	94
NNU_28747	597	NNU_024547	800	0.98	44	4.56E-14	42
NNU_28748	1140	NNU_012832	2403	1.00	80	1.85E-35	81
NNU_28748	1140	NNU_009420	2846	0.89	78	2.45E-19	52
NNU_28750	3672	NNU_026508	858	0.84	873	0	440
NNU_28750	3672	NNU_026507	279	0.94	181	9.56E-73	149
NNU_28752	723	NNU_001168	1290	0.95	148	4.03E-60	125
NNU_28752	723	NNU_011457	1234	0.87	88	4.26E-20	53
NNU_28754	674	NNU_008568	1751	0.82	257	1.36E-54	115
NNU_28764	1178	NNU_011440	1806	0.89	186	2.39E-59	124
NNU_28765	4491	NNU_017394	2550	0.82	133	9.72E-24	61
NNU_28766	1958	NNU_017589	578	0.87	259	1.40E-78	159
NNU_28768	6088	NNU_023767	381	0.88	135	2.16E-36	84
NNU_28768	6088	NNU_006968	1126	0.95	40	3.75E-09	35
NNU_28768	6088	NNU_020232	1772	0.95	36	6.27E-07	31

NNU_28768	6088	NNU_011106	3772	0.97	32	2.26E-06	30
NNU_28769	6099	NNU_020676	198	0.91	102	6.06E-32	76
NNU_28773	917	NNU_014831	2029	0.90	413	5.82E-154	294
NNU_28776	474	NNU_001932	1260	0.81	137	1.27E-23	59
NNU_28777	6989	NNU_023123	1803	0.88	170	2.43E-51	111
NNU_28777	6989	NNU_018179	2813	0.88	158	8.81E-46	101
NNU_28777	6989	NNU_018192	1620	0.89	99	7.00E-27	67
NNU_28777	6989	NNU_008174	2745	0.95	38	5.57E-08	33
NNU_28777	6989	NNU_020244	2056	0.93	40	7.20E-07	31
NNU_28780	3875	NNU_010096	3417	0.93	152	1.33E-56	120
NNU_28781	998	NNU_016507	1238	0.81	73	3.63E-07	30
NNU_28783	3121	NNU_012895	1911	0.88	212	4.93E-65	135
NNU_28783	3121	NNU_011702	1845	0.85	110	3.14E-22	58
NNU_28783	3121	NNU_015766	1262	1.00	50	2.44E-18	51
NNU_28785	1082	NNU_010449	7439	0.94	95	2.27E-34	79
NNU_28785	1082	NNU_024928	957	0.94	152	7.89E-59	123
NNU_28785	1082	NNU_013547	7682	0.87	115	6.36E-30	71
NNU_28789	1124	NNU_012311	1306	0.89	51	2.45E-09	34
NNU_28790	1626	NNU_026200	730	0.92	109	2.05E-36	83
NNU_28790	1626	NNU_000448	806	0.96	45	1.64E-12	40
NNU_28790	1626	NNU_002847	1707	0.84	521	1.78E-136	263
NNU_28790	1626	NNU_002846	1692	0.84	494	1.07E-133	258
NNU_28790	1626	NNU_019361	318	0.93	122	1.22E-43	96
NNU_28790	1626	NNU_008979	254	0.83	167	1.24E-33	78
NNU_28791	3250	NNU_018787	1068	0.94	85	6.97E-29	70
NNU_28791	3250	NNU_001017	5170	0.98	51	3.29E-17	49
NNU_28791	3250	NNU_006459	1602	0.96	45	3.31E-12	40
NNU_28793	1315	NNU_000448	806	0.96	45	1.33E-12	40
NNU_28793	1315	NNU_026200	730	0.92	109	1.66E-36	83
NNU_28793	1315	NNU_002847	1707	0.82	290	2.66E-64	133
NNU_28793	1315	NNU_008979	254	0.83	166	3.59E-33	77
NNU_28793	1315	NNU_019361	318	0.91	87	6.05E-26	64
NNU_28797	732	NNU_019278	2216	0.89	175	1.48E-54	115
NNU_28801	854	NNU_020456	1926	0.90	92	3.00E-27	66
NNU_28805	2320	NNU_000767	889	1.00	40	6.56E-13	41
NNU_28811	1642	NNU_015471	525	0.91	225	7.00E-81	163
NNU_28811	1642	NNU_003211	2967	0.88	233	3.28E-74	151
NNU_28812	1606	NNU_008990	1250	0.85	742	0	391
NNU_28812	1606	NNU_004694	1266	0.90	458	2.17E-170	324
NNU_28813	3044	NNU_001911	3248	0.80	293	3.79E-51	110
NNU_28814	2503	NNU_001908	2718	0.86	499	1.63E-148	285
NNU_28815	806	NNU_001886	1377	0.87	295	2.01E-88	176
NNU_28816	824	NNU_006163	690	1.00	67	2.24E-28	68
NNU_28818	971	NNU_026411	1916	0.82	309	5.42E-65	134

NNU_28819	1700	NNU_014024	1499	0.85	247	7.46E-61	127
NNU_28819	1700	NNU_021510	4261	0.97	115	4.55E-48	104
NNU_28821	1129	NNU_020034	1148	0.84	622	3.26E-172	327
NNU_28829	2573	NNU_020141	1094	0.89	815	0	528
NNU_28829	2573	NNU_003103	867	0.89	305	8.29E-102	201
NNU_28829	2573	NNU_020911	1942	0.87	266	2.39E-77	157
NNU_28829	2573	NNU_008212	1914	0.94	110	1.51E-39	89
NNU_28829	2573	NNU_021319	2166	0.90	120	2.53E-37	85
NNU_28829	2573	NNU_010094	3573	1.00	43	1.56E-14	44
NNU_28831	947	NNU_020635	955	0.94	308	1.34E-130	252
NNU_28831	947	NNU_015481	1327	0.92	224	1.10E-86	173
NNU_28831	947	NNU_023123	1803	0.91	89	3.34E-27	66
NNU_28831	947	NNU_003163	4594	0.98	50	3.39E-17	48
NNU_28833	2310	NNU_020141	1094	0.89	815	0	528
NNU_28833	2310	NNU_003103	867	0.89	305	7.44E-102	201
NNU_28833	2310	NNU_020911	1942	0.87	267	5.96E-78	158
NNU_28833	2310	NNU_008212	1914	0.94	110	1.35E-39	89
NNU_28833	2310	NNU_021319	2166	0.90	120	2.27E-37	85
NNU_28833	2310	NNU_010094	3573	1.00	43	1.40E-14	44
NNU_28834	2085	NNU_002949	2466	0.87	262	1.49E-78	159
NNU_28834	2085	NNU_005948	3374	0.90	189	4.24E-64	133
NNU_28834	2085	NNU_017127	951	0.91	100	2.66E-31	74
NNU_28834	2085	NNU_021409	210	0.91	100	2.66E-31	74
NNU_28836	3990	NNU_021097	5202	0.92	1146	0	859
NNU_28836	3990	NNU_009478	1294	0.87	403	2.07E-129	251
NNU_28836	3990	NNU_020461	999	0.96	135	1.78E-55	118
NNU_28836	3990	NNU_004475	691	0.95	55	5.23E-16	47
NNU_28837	836	NNU_019074	1785	0.92	86	2.94E-27	66
NNU_28838	707	NNU_014145	835	0.94	51	1.51E-14	43
NNU_28838	707	NNU_020169	1194	0.88	106	6.87E-28	67
NNU_28842	2673	NNU_026562	660	0.98	51	2.70E-17	49
NNU_28842	2673	NNU_026456	961	0.89	174	5.53E-54	115
NNU_28842	2673	NNU_026537	251	0.90	160	7.15E-53	113
NNU_28842	2673	NNU_015157	549	0.89	158	2.00E-48	105
NNU_28842	2673	NNU_018691	1866	0.95	55	3.50E-16	47
NNU_28842	2673	NNU_006163	690	0.93	76	5.77E-24	61
NNU_28843	177	NNU_006163	690	1.00	71	2.58E-31	72
NNU_28845	739	NNU_011399	1687	0.86	401	8.19E-117	227
NNU_28845	739	NNU_004871	1827	0.91	273	3.01E-101	199
NNU_28846	521	NNU_018102	4107	0.98	87	2.30E-36	82
NNU_28846	521	NNU_001090	3043	0.97	59	8.44E-21	54
NNU_28847	2348	NNU_025674	1577	0.85	1172	0	642
NNU_28847	2348	NNU_020668	4831	0.89	125	4.99E-34	79
NNU_28847	2348	NNU_025173	3253	0.86	91	1.42E-19	53

NNU_28847	2348	NNU_006219	2926	0.94	46	1.11E-10	37
NNU_28849	2034	NNU_010311	631	0.95	61	1.23E-19	53
NNU_28856	3028	NNU_003419	2088	0.80	301	2.25E-53	114
NNU_28858	1859	NNU_025539	2646	0.93	1009	0	793
NNU_28858	1859	NNU_012068	1650	0.92	363	1.56E-147	283
NNU_28858	1859	NNU_020512	795	0.93	513	0	414
NNU_28858	1859	NNU_026066	2602	0.80	995	0	395
NNU_28858	1859	NNU_005177	345	0.91	338	5.76E-127	246
NNU_28858	1859	NNU_019302	300	0.88	292	7.77E-96	190
NNU_28858	1859	NNU_026178	1896	1.00	30	1.90E-07	31
NNU_28861	981	NNU_008540	870	1.00	29	3.56E-07	30
NNU_28861	981	NNU_003570	1390	0.95	128	3.34E-52	111
NNU_28861	981	NNU_008738	495	0.91	135	4.35E-46	100
NNU_28861	981	NNU_017733	1061	0.94	35	1.28E-06	29
NNU_28862	670	NNU_005197	4773	0.92	114	1.38E-39	88
NNU_28862	670	NNU_004498	794	0.86	108	1.09E-25	63
NNU_28865	2434	NNU_012101	2352	0.95	152	1.78E-63	132
NNU_28865	2434	NNU_008855	2424	0.88	274	2.23E-87	175
NNU_28865	2434	NNU_003848	3709	0.90	138	5.10E-44	97
NNU_28865	2434	NNU_003851	3204	0.84	130	6.74E-28	68
NNU_28865	2434	NNU_024054	826	0.95	43	1.15E-10	37
NNU_28865	2434	NNU_018466	984	0.95	39	5.36E-09	34
NNU_28865	2434	NNU_009394	1833	1.00	33	5.36E-09	34
NNU_28871	5629	NNU_005921	1509	0.95	58	1.59E-17	50
NNU_28871	5629	NNU_000105	8491	0.96	49	3.44E-14	44
NNU_28871	5629	NNU_023190	3293	0.94	48	5.76E-12	40
NNU_28871	5629	NNU_022848	3240	1.00	28	7.50E-06	29
NNU_28872	1306	NNU_014495	1994	0.93	213	3.32E-83	167
NNU_28872	1306	NNU_002958	2059	0.97	185	9.23E-84	168
NNU_28874	678	NNU_011232	593	0.98	47	1.12E-15	45
NNU_28878	3230	NNU_006016	2196	0.91	64	4.23E-16	47
NNU_28879	2581	NNU_004581	1181	0.91	55	7.30E-13	41
NNU_28883	2120	NNU_003938	1461	0.88	237	3.31E-70	144
NNU_28883	2120	NNU_004346	1190	0.94	71	1.64E-23	60
NNU_28883	2120	NNU_018291	2135	0.95	54	9.95E-16	46
NNU_28883	2120	NNU_015956	4289	1.00	31	6.03E-08	32
NNU_28884	981	NNU_016533	1944	0.96	168	5.40E-75	152
NNU_28885	3713	NNU_004207	865	1.00	29	1.37E-06	30
NNU_28886	1489	NNU_007199	1015	0.91	125	1.86E-41	92
NNU_28886	1489	NNU_017830	2367	0.78	362	8.43E-60	125
NNU_28886	1489	NNU_017823	1441	0.80	236	4.01E-43	95
NNU_28886	1489	NNU_019906	806	0.97	32	5.45E-07	30
NNU_28887	3134	NNU_012101	2352	0.95	152	1.07E-61	129
NNU_28887	3134	NNU_008855	2424	0.91	189	3.82E-66	137

NNU_28887	3134	NNU_003848	3709	0.90	138	6.58E-44	97
NNU_28887	3134	NNU_003851	3204	0.84	130	8.70E-28	68
NNU_28887	3134	NNU_018466	984	0.95	39	6.92E-09	34
NNU_28887	3134	NNU_009394	1833	1.00	33	6.92E-09	34
NNU_28887	3134	NNU_024054	826	0.93	43	6.92E-09	34
NNU_28893	2199	NNU_019118	1347	0.84	1187	0	630
NNU_28895	1563	NNU_018643	1709	0.86	354	1.39E-102	202
NNU_28898	1025	NNU_023333	3078	0.94	53	1.71E-15	45
NNU_28903	1713	NNU_010538	6731	0.94	274	2.50E-115	225
NNU_28903	1713	NNU_020635	955	0.96	103	2.15E-41	92
NNU_28903	1713	NNU_009893	1567	0.85	163	7.73E-41	91
NNU_28903	1713	NNU_015481	1327	0.89	128	1.00E-39	89
NNU_28903	1713	NNU_011697	1843	0.97	91	2.17E-36	83
NNU_28903	1713	NNU_023123	1803	0.92	89	1.31E-28	69
NNU_28903	1713	NNU_003163	4594	0.97	61	2.21E-21	56
NNU_28906	1286	NNU_024796	1375	0.86	92	3.58E-18	50
NNU_28906	1286	NNU_012898	1785	0.92	137	9.54E-49	105
NNU_28909	2505	NNU_025890	2498	0.93	521	0	408
NNU_28909	2505	NNU_007445	690	0.94	119	4.06E-45	99
NNU_28909	2505	NNU_026362	1544	0.96	47	7.09E-13	41
NNU_28909	2505	NNU_016698	2040	0.87	544	2.61E-176	335
NNU_28909	2505	NNU_007444	1545	0.85	246	1.42E-64	134
NNU_28909	2505	NNU_007740	4566	0.93	87	5.36E-29	70
NNU_28909	2505	NNU_001988	853	0.81	119	5.44E-19	52
NNU_28909	2505	NNU_005639	1620	0.92	62	9.10E-17	48
NNU_28911	676	NNU_025173	3253	0.94	48	6.70E-13	40
NNU_28911	676	NNU_006219	2926	0.92	48	1.12E-10	36
NNU_28912	1159	NNU_023328	3699	0.90	67	1.50E-16	47
NNU_28916	851	NNU_026415	1804	0.89	230	1.30E-75	153
NNU_28916	851	NNU_023534	891	0.86	249	1.01E-71	146
NNU_28918	5402	NNU_018708	6151	0.96	225	4.86E-102	202
NNU_28918	5402	NNU_001397	1838	0.86	210	1.86E-56	120
NNU_28918	5402	NNU_021975	1059	0.91	134	8.79E-45	99
NNU_28918	5402	NNU_021976	2883	0.91	131	4.09E-43	96
NNU_28918	5402	NNU_021697	432	0.88	146	4.09E-43	96
NNU_28918	5402	NNU_015566	813	0.87	93	5.44E-22	58
NNU_28918	5402	NNU_024129	1491	0.96	49	3.30E-14	44
NNU_28918	5402	NNU_002750	2172	1.00	28	7.19E-06	29
NNU_28919	1235	NNU_016696	2793	0.94	461	0	380
NNU_28919	1235	NNU_021168	1704	0.83	242	1.97E-50	108
NNU_28922	1207	NNU_020540	843	0.93	106	1.52E-36	83
NNU_28922	1207	NNU_017467	1667	0.95	76	1.54E-26	65
NNU_28923	7523	NNU_001144	2131	0.82	816	0	360
NNU_28923	7523	NNU_019395	4780	0.87	59	1.29E-09	36

NNU_28924	1192	NNU_008368	1084	1.00	44	1.99E-15	45
NNU_28926	3004	NNU_004920	1787	0.94	1569	0	1278
NNU_28927	1354	NNU_023422	1046	0.89	397	1.92E-135	261
NNU_28929	2221	NNU_020769	1883	0.94	64	1.34E-19	53
NNU_28930	565	NNU_015737	3181	0.96	159	1.11E-69	142
NNU_28932	6995	NNU_010754	548	0.83	170	1.16E-34	81
NNU_28934	2345	NNU_020769	1883	0.94	64	1.41E-19	53
NNU_28938	762	NNU_010677	3117	0.90	377	2.29E-137	264
NNU_28943	688	NNU_011226	2535	0.97	66	1.45E-24	61
NNU_28943	688	NNU_011227	488	0.96	152	1.06E-65	135
NNU_28943	688	NNU_023931	3240	0.86	96	1.88E-18	50
NNU_28943	688	NNU_007456	3350	0.88	68	1.13E-15	45
NNU_28947	6098	NNU_016168	548	0.98	315	1.10E-153	295
NNU_28947	6098	NNU_024230	945	0.92	58	1.34E-13	43
NNU_28948	903	NNU_010824	1317	0.98	102	6.69E-44	96
NNU_28948	903	NNU_012294	734	0.98	100	8.66E-43	94
NNU_28948	903	NNU_025919	2427	0.92	67	1.93E-19	52
NNU_28948	903	NNU_008146	846	1.00	33	1.96E-09	34
NNU_28948	903	NNU_013877	1412	0.86	98	3.20E-22	57
NNU_28949	2534	NNU_011501	6083	0.97	150	8.58E-67	138
NNU_28949	2534	NNU_011500	1982	0.95	41	1.55E-09	35
NNU_28954	2661	NNU_011609	1392	0.92	129	1.20E-45	100
NNU_28954	2661	NNU_007684	1038	0.90	93	2.65E-27	67
NNU_28955	755	NNU_018547	694	0.81	312	1.51E-59	124
NNU_28955	755	NNU_009639	2438	0.87	100	2.06E-23	59
NNU_28955	755	NNU_010316	2507	0.88	72	9.65E-17	47
NNU_28956	1929	NNU_008587	693	0.88	56	3.27E-10	36
NNU_28957	1467	NNU_000490	1074	0.81	412	1.33E-87	175
NNU_28957	1467	NNU_022223	1549	0.94	152	1.79E-61	128
NNU_28957	1467	NNU_022447	739	0.97	71	5.22E-27	66
NNU_28957	1467	NNU_015615	1467	0.96	49	8.85E-15	44
NNU_28967	4799	NNU_000191	483	0.92	422	6.54E-170	324
NNU_28967	4799	NNU_001477	1002	0.82	127	6.25E-21	56
NNU_28968	1386	NNU_021251	2214	0.93	404	4.04E-167	318
NNU_28968	1386	NNU_011227	488	0.95	255	1.21E-112	220
NNU_28968	1386	NNU_011226	2535	0.94	67	1.78E-21	56
NNU_28968	1386	NNU_023931	3240	0.86	96	3.86E-18	50
NNU_28970	5069	NNU_025920	13099	0.85	100	8.55E-20	54
NNU_28972	2430	NNU_011381	381	0.97	35	1.92E-08	33
NNU_28977	897	NNU_006903	340	0.97	37	5.40E-10	35
NNU_28977	897	NNU_021621	1119	0.95	36	9.04E-08	31
NNU_28978	2961	NNU_003378	1052	0.93	56	5.01E-15	45
NNU_28978	2961	NNU_022055	324	1.00	33	6.53E-09	34
NNU_28978	2961	NNU_011118	3176	0.80	143	1.38E-20	55

NNU_28978	2961	NNU_018686	1056	0.93	60	3.00E-17	49
NNU_28978	2961	NNU_019632	501	0.93	60	3.00E-17	49
NNU_28978	2961	NNU_005096	297	0.94	35	3.93E-06	29
NNU_28987	482	NNU_011232	593	0.90	260	7.05E-91	180
NNU_28987	482	NNU_008441	1168	0.90	61	2.82E-15	44
NNU_28987	482	NNU_006968	1126	0.95	42	7.89E-11	36
NNU_28987	482	NNU_002653	2707	0.93	41	1.32E-08	32
NNU_28988	849	NNU_012834	354	0.91	100	1.38E-30	72
NNU_28995	2557	NNU_021459	2463	0.92	112	6.98E-38	86
NNU_28995	2557	NNU_008777	3527	0.73	1406	2.82E-136	263
NNU_28995	2557	NNU_015448	492	0.94	227	1.39E-94	188
NNU_28995	2557	NNU_015335	1380	0.87	196	3.16E-56	119
NNU_28995	2557	NNU_002493	1433	0.86	117	2.55E-27	67
NNU_28999	882	NNU_014507	2971	0.94	259	4.61E-110	215
NNU_28999	882	NNU_011446	1556	0.97	190	2.20E-88	176
NNU_28999	882	NNU_010229	271	0.98	185	2.85E-87	174
NNU_28999	882	NNU_002573	2001	0.88	187	6.40E-59	123
NNU_28999	882	NNU_024547	800	0.97	127	4.98E-55	116
NNU_28999	882	NNU_008264	651	0.95	110	6.53E-44	96
NNU_29006	1224	NNU_010614	2304	0.98	42	1.23E-12	40
NNU_29007	1266	NNU_019029	3084	0.93	132	1.22E-47	103
NNU_29009	1249	NNU_012817	870	0.89	206	4.22E-62	129
NNU_29010	2629	NNU_020911	1942	0.91	382	7.95E-147	282
NNU_29010	2629	NNU_010376	799	0.91	356	2.24E-137	265
NNU_29010	2629	NNU_007644	517	0.90	274	8.53E-97	192
NNU_29011	975	NNU_003172	390	0.91	300	8.48E-113	220
NNU_29016	1999	NNU_021693	2329	0.84	143	9.17E-31	73
NNU_29021	7803	NNU_020055	1521	0.91	113	2.77E-36	84
NNU_29021	7803	NNU_010499	1645	0.89	113	2.79E-31	75
NNU_29021	7803	NNU_024142	855	0.89	70	1.03E-15	47
NNU_29021	7803	NNU_020037	5013	0.87	53	6.21E-08	33
NNU_29022	2745	NNU_004737	1642	0.88	93	5.92E-24	61
NNU_29030	518	NNU_014840	1005	0.91	437	4.06E-173	328
NNU_29030	518	NNU_020839	1449	0.91	437	4.08E-168	319
NNU_29037	1324	NNU_021762	334	0.95	55	1.72E-16	47
NNU_29038	2786	NNU_022143	723	0.97	36	6.14E-09	34
NNU_29039	818	NNU_013504	750	0.93	116	2.81E-42	93
NNU_29041	550	NNU_002946	679	0.84	330	1.76E-82	165
NNU_29042	2038	NNU_011677	240	0.94	211	5.17E-88	176
NNU_29048	1243	NNU_019579	2292	0.94	205	6.78E-85	170
NNU_29048	1243	NNU_011543	462	0.90	161	7.08E-55	116
NNU_29048	1243	NNU_015766	1262	0.99	88	3.36E-38	86
NNU_29048	1243	NNU_008078	492	0.92	87	1.23E-27	67
NNU_29049	2936	NNU_000044	1935	0.90	599	0	413

NNU_29049	2936	NNU_019609	798	0.95	419	0	359
NNU_29049	2936	NNU_022735	2216	0.95	424	0	358
NNU_29049	2936	NNU_017016	1350	0.88	472	2.44E-157	301
NNU_29049	2936	NNU_009334	1344	0.84	468	1.18E-125	244
NNU_29049	2936	NNU_017191	1101	0.93	222	1.61E-89	179
NNU_29049	2936	NNU_024360	1156	0.91	237	2.08E-88	177
NNU_29049	2936	NNU_010456	2864	0.85	190	1.70E-49	107
NNU_29049	2936	NNU_004799	2484	0.88	147	2.87E-42	94
NNU_29050	2133	NNU_007873	2369	0.81	309	2.02E-62	130
NNU_29051	4345	NNU_004084	627	0.90	600	0	396
NNU_29053	2140	NNU_018295	1659	0.92	95	1.27E-29	71
NNU_29053	2140	NNU_000105	8491	0.92	95	1.27E-29	71
NNU_29053	2140	NNU_005932	2459	0.85	101	2.77E-21	56
NNU_29053	2140	NNU_023190	3293	1.00	35	3.64E-10	36
NNU_29054	1891	NNU_003289	864	0.95	728	0	619
NNU_29054	1891	NNU_013064	905	0.93	89	3.12E-30	72
NNU_29054	1891	NNU_021196	464	0.90	89	3.14E-25	63
NNU_29054	1891	NNU_021184	740	0.97	65	1.46E-23	60
NNU_29054	1891	NNU_019902	543	0.89	88	5.26E-23	59
NNU_29054	1891	NNU_001639	357	0.96	55	5.29E-18	50
NNU_29055	3385	NNU_019470	6016	0.85	133	4.34E-31	74
NNU_29055	3385	NNU_013312	857	0.87	133	1.55E-35	82
NNU_29055	3385	NNU_001805	2153	0.82	109	9.52E-18	50
NNU_29056	1475	NNU_022143	723	0.89	344	2.13E-120	234
NNU_29065	413	NNU_023804	1092	0.86	175	2.28E-50	107
NNU_29065	413	NNU_001324	997	0.97	34	1.12E-08	32
NNU_29069	2697	NNU_000666	1137	0.80	1015	0	411
NNU_29073	4615	NNU_014511	1173	0.88	100	1.66E-26	66
NNU_29081	5042	NNU_006983	213	0.89	165	2.27E-50	109
NNU_29081	5042	NNU_025660	459	0.91	103	1.80E-31	75
NNU_29081	5042	NNU_008046	3016	0.93	78	3.04E-24	62
NNU_29081	5042	NNU_002638	1239	0.98	62	3.93E-23	60
NNU_29081	5042	NNU_011497	1252	0.92	62	1.84E-16	48
NNU_29085	691	NNU_004329	1143	0.98	80	2.40E-32	75
NNU_29086	546	NNU_025820	1162	0.89	160	8.50E-51	108
NNU_29086	546	NNU_021602	945	0.98	199	2.87E-95	188
NNU_29086	546	NNU_023845	276	0.87	181	1.84E-47	102
NNU_29086	546	NNU_025821	828	0.97	67	3.17E-25	62
NNU_29087	853	NNU_025820	1162	0.96	220	2.10E-98	194
NNU_29087	853	NNU_021602	945	0.98	166	6.01E-79	159
NNU_29087	853	NNU_006230	2901	0.92	211	2.16E-78	158
NNU_29087	853	NNU_023844	1608	0.97	117	1.74E-49	106
NNU_29094	1897	NNU_017211	870	0.93	574	0	460
NNU_29095	1136	NNU_023185	399	0.82	255	8.35E-54	114

NNU_29098	923	NNU_012854	525	1.00	49	2.55E-18	50
NNU_29098	923	NNU_009636	2013	1.00	49	2.55E-18	50
NNU_29099	6349	NNU_009613	1062	0.92	100	1.76E-32	77
NNU_29100	6239	NNU_017182	504	1.00	78	1.33E-33	79
NNU_29107	1258	NNU_003562	3733	0.95	38	9.88E-09	33
NNU_29108	1351	NNU_017805	417	0.92	168	2.13E-60	126
NNU_29115	1027	NNU_026106	1451	0.87	169	2.73E-48	104
NNU_29116	1740	NNU_017865	2289	0.91	64	6.29E-17	48
NNU_29122	2250	NNU_000489	1131	0.91	256	1.58E-93	186
NNU_29122	2250	NNU_020788	1443	0.84	181	1.69E-43	96
NNU_29125	1865	NNU_004127	3180	0.85	103	6.70E-22	57
NNU_29125	1865	NNU_021076	1228	0.95	182	6.20E-77	156
NNU_29133	3376	NNU_009937	1083	0.95	54	5.72E-15	45
NNU_29133	3376	NNU_021979	1973	0.97	36	7.45E-09	34
NNU_29136	904	NNU_001743	4168	0.84	495	5.94E-129	249
NNU_29138	2033	NNU_001780	600	0.78	364	1.17E-54	116
NNU_29138	2033	NNU_011824	2591	0.78	197	4.37E-24	61
NNU_29139	1338	NNU_018685	1431	0.89	222	9.59E-74	150
NNU_29139	1338	NNU_005096	297	0.87	222	7.52E-65	134
NNU_29139	1338	NNU_022055	324	0.95	37	3.78E-08	32
NNU_29140	3654	NNU_024819	366	1.00	58	1.02E-22	59
NNU_29140	3654	NNU_023316	441	0.96	54	3.70E-17	49
NNU_29140	3654	NNU_015040	1307	0.95	97	4.65E-36	83
NNU_29140	3654	NNU_015631	243	1.00	29	1.35E-06	30
NNU_29141	1190	NNU_008874	477	0.89	366	2.19E-129	250
NNU_29142	4015	NNU_017499	1205	0.89	62	3.17E-13	42
NNU_29142	4015	NNU_009390	1363	0.89	62	3.17E-13	42
NNU_29144	902	NNU_016507	1238	0.91	64	1.16E-16	47
NNU_29144	902	NNU_009451	3613	0.86	79	1.16E-16	47
NNU_29144	902	NNU_013492	2276	0.90	60	1.94E-14	43
NNU_29144	902	NNU_000605	1684	0.90	60	1.94E-14	43
NNU_29144	902	NNU_015993	3325	0.93	53	6.98E-14	42
NNU_29144	902	NNU_005729	1706	0.88	56	1.51E-10	36
NNU_29144	902	NNU_004797	1906	0.88	50	2.53E-08	32
NNU_29149	918	NNU_026028	2517	0.88	208	5.12E-65	134
NNU_29149	918	NNU_000028	1170	0.92	200	2.35E-73	149
NNU_29152	326	NNU_003570	1390	0.92	85	1.09E-27	66
NNU_29152	326	NNU_008540	870	1.00	50	2.39E-19	51
NNU_29152	326	NNU_026530	621	0.97	35	2.42E-09	33
NNU_29152	326	NNU_026542	1563	1.00	31	8.71E-09	32
NNU_29152	326	NNU_014667	468	1.00	31	8.71E-09	32
NNU_29152	326	NNU_014546	1752	1.00	31	8.71E-09	32
NNU_29156	2126	NNU_020540	843	0.91	172	4.35E-59	124
NNU_29156	2126	NNU_003804	3759	0.89	154	4.42E-49	106

NNU_29156	2126	NNU_017467	1667	0.98	95	3.46E-40	90
NNU_29156	2126	NNU_014170	1245	0.92	117	4.48E-39	88
NNU_29156	2126	NNU_023675	3348	0.94	49	6.00E-13	41
NNU_29156	2126	NNU_020594	1389	0.84	77	6.00E-13	41
NNU_29157	9117	NNU_017520	813	0.88	83	1.54E-19	54
NNU_29159	969	NNU_004127	3180	0.93	174	8.99E-68	139
NNU_29166	1831	NNU_017895	2776	0.99	82	1.08E-34	80
NNU_29166	1831	NNU_016300	347	0.96	88	5.02E-33	77
NNU_29169	965	NNU_010140	3276	0.97	32	3.50E-07	30
NNU_29170	671	NNU_004589	3138	0.97	157	3.67E-70	143
NNU_29173	1932	NNU_004610	768	0.78	197	4.15E-24	61
NNU_29174	4684	NNU_011943	1718	0.82	77	8.01E-10	36
NNU_29175	3010	NNU_004635	636	0.92	142	3.75E-51	110
NNU_29179	3905	NNU_004670	1551	0.91	110	6.44E-35	81
NNU_29180	2185	NNU_026090	1197	0.84	208	7.60E-47	102
NNU_29180	2185	NNU_010557	1044	0.83	175	3.59E-35	81
NNU_29180	2185	NNU_015333	783	0.80	207	4.64E-34	79
NNU_29180	2185	NNU_013168	3900	0.88	55	1.03E-10	37
NNU_29181	787	NNU_024089	966	0.95	57	7.78E-18	49
NNU_29181	787	NNU_009077	111	0.97	58	4.65E-20	53
NNU_29182	972	NNU_005364	2328	0.96	98	7.27E-39	87
NNU_29182	972	NNU_000654	354	0.96	91	2.04E-34	79
NNU_29182	972	NNU_009472	1173	0.92	77	2.67E-23	59
NNU_29182	972	NNU_014622	1068	0.97	38	1.63E-10	36
NNU_29183	2209	NNU_004037	735	1.00	29	8.13E-07	30
NNU_29186	672	NNU_025820	1162	0.82	292	1.72E-63	131
NNU_29186	672	NNU_006230	2901	0.90	211	7.91E-72	146
NNU_29186	672	NNU_021602	945	0.94	49	6.66E-13	40
NNU_29188	4748	NNU_022951	4362	0.96	44	1.74E-11	39
NNU_29190	1688	NNU_026227	994	0.92	327	8.74E-125	242
NNU_29190	1688	NNU_009192	1273	0.84	185	2.74E-40	90
NNU_29190	1688	NNU_012538	1999	0.88	93	1.68E-22	58
NNU_29192	1495	NNU_003217	2653	0.92	49	1.95E-11	38
NNU_29192	1495	NNU_026673	1164	0.97	107	2.41E-45	99
NNU_29193	3418	NNU_007427	2543	0.96	45	3.48E-12	40
NNU_29197	2051	NNU_009484	1896	0.93	384	1.68E-162	310
NNU_29198	7061	NNU_009336	1949	0.86	144	9.02E-36	83
NNU_29201	4886	NNU_019568	10194	0.86	147	4.82E-37	85
NNU_29203	741	NNU_002467	1555	1.00	44	1.22E-15	45
NNU_29208	1357	NNU_012594	3874	0.90	182	9.94E-59	123
NNU_29208	1357	NNU_025026	399	0.98	49	1.76E-16	47
NNU_29209	1582	NNU_015984	1047	0.96	106	4.26E-43	95
NNU_29209	1582	NNU_014851	3748	0.96	91	9.29E-35	80
NNU_29209	1582	NNU_012246	474	1.00	35	2.68E-10	36

NNU_29209	1582	NNU_002736	601	1.00	30	1.61E-07	31
NNU_29209	1582	NNU_006493	318	1.00	30	1.61E-07	31
NNU_29217	315	NNU_020291	428	0.96	135	1.31E-56	118
NNU_29217	315	NNU_026316	544	0.96	105	7.98E-44	95
NNU_29219	1274	NNU_012973	1968	0.94	416	1.02E-177	337
NNU_29219	1274	NNU_018618	1264	0.96	140	9.32E-59	123
NNU_29222	1317	NNU_020169	1194	0.87	179	5.84E-51	109
NNU_29222	1317	NNU_026380	813	0.77	286	3.57E-38	86
NNU_29223	1523	NNU_016314	519	0.92	267	4.87E-102	201
NNU_29228	1480	NNU_004621	633	0.89	632	0	417
NNU_29229	487	NNU_018019	1899	0.98	96	1.64E-42	93
NNU_29238	1372	NNU_026083	1558	0.84	316	2.10E-80	162
NNU_29239	1055	NNU_013963	1894	0.93	102	4.76E-36	82
NNU_29241	2573	NNU_000633	1232	0.98	89	9.09E-37	84
NNU_29241	2573	NNU_000634	778	0.85	125	5.51E-29	70
NNU_29245	1153	NNU_007106	4644	0.94	111	5.17E-41	91
NNU_29246	834	NNU_010543	351	0.91	191	2.77E-67	138
NNU_29247	4417	NNU_000875	543	0.94	106	2.60E-39	89
NNU_29247	4417	NNU_012344	2709	0.93	152	1.52E-56	120
NNU_29247	4417	NNU_005747	2132	0.92	256	2.39E-99	197
NNU_29247	4417	NNU_022019	2538	0.98	53	3.46E-18	51
NNU_29248	1176	NNU_010647	426	0.80	95	1.98E-10	36
NNU_29249	894	NNU_000371	1218	0.93	106	2.40E-38	86
NNU_29249	894	NNU_017712	357	0.92	82	5.27E-25	62
NNU_29252	1920	NNU_004250	3927	0.95	255	1.02E-109	215
NNU_29256	974	NNU_025864	1555	0.92	237	4.06E-91	181
NNU_29257	3244	NNU_018324	1794	0.94	491	0	402
NNU_29266	719	NNU_023908	1740	0.92	151	4.03E-55	116
NNU_29268	5780	NNU_021683	2319	0.91	196	3.27E-69	143
NNU_29268	5780	NNU_006591	5446	0.88	358	1.83E-116	228
NNU_29268	5780	NNU_012719	3170	0.85	70	7.65E-11	38
NNU_29268	5780	NNU_026398	4103	0.88	51	1.66E-07	32
NNU_29269	2906	NNU_017173	1632	1.00	51	6.32E-19	52
NNU_29272	700	NNU_019062	1796	0.80	194	3.14E-31	73
NNU_29272	700	NNU_004508	1725	0.91	110	1.12E-35	81
NNU_29272	700	NNU_006099	739	0.80	118	3.21E-16	46
NNU_29273	1922	NNU_022414	2268	0.87	535	9.42E-165	314
NNU_29273	1922	NNU_005197	4773	0.90	136	5.19E-43	95
NNU_29273	1922	NNU_001211	942	0.82	145	2.47E-26	65
NNU_29275	2099	NNU_020203	1503	0.85	634	0	345
NNU_29276	695	NNU_002233	234	0.88	111	3.12E-31	73
NNU_29276	695	NNU_012311	1306	0.88	56	1.15E-10	36
NNU_29280	623	NNU_025504	1083	0.90	101	1.00E-30	72
NNU_29281	2561	NNU_011902	1518	0.93	89	4.24E-30	72

NNU_29283	1322	NNU_001468	1254	0.93	70	1.70E-21	56
NNU_29283	1322	NNU_000192	2034	0.94	51	2.87E-14	43
NNU_29285	6183	NNU_025760	258	0.83	197	1.69E-42	95
NNU_29285	6183	NNU_003003	1269	0.94	52	3.78E-14	44
NNU_29289	794	NNU_020528	1017	0.91	42	7.97E-08	31
NNU_29291	2856	NNU_015625	3082	0.78	1955	0	655
NNU_29292	783	NNU_014832	633	0.97	33	7.85E-08	31
NNU_29293	8113	NNU_014877	1511	0.96	97	2.23E-37	86
NNU_29293	8113	NNU_012469	4775	0.78	200	1.76E-23	61
NNU_29298	2669	NNU_019652	1451	0.87	143	3.39E-36	83
NNU_29300	2112	NNU_021104	930	0.90	384	1.80E-137	265
NNU_29300	2112	NNU_009096	952	0.98	45	4.61E-14	43
NNU_29300	2112	NNU_025660	459	0.94	110	9.56E-41	91
NNU_29301	1963	NNU_018264	264	0.91	163	3.13E-55	117
NNU_29301	1963	NNU_009779	3297	0.99	124	5.20E-58	122
NNU_29302	435	NNU_004878	2286	0.97	371	1.56E-176	334
NNU_29310	1092	NNU_026304	1101	0.91	201	4.69E-71	145
NNU_29310	1092	NNU_009903	666	0.89	80	1.81E-20	54
NNU_29313	1370	NNU_004749	1540	0.98	197	2.05E-95	189
NNU_29313	1370	NNU_024236	1095	0.90	132	6.17E-41	91
NNU_29314	561	NNU_005346	635	0.88	119	1.93E-32	75
NNU_29317	1586	NNU_019371	2087	0.92	671	0	523
NNU_29317	1586	NNU_013117	2635	0.77	613	3.12E-84	169
NNU_29320	607	NNU_009422	968	0.98	47	9.95E-16	45
NNU_29321	2838	NNU_023528	4032	0.94	77	4.74E-25	63
NNU_29321	2838	NNU_000969	1502	1.00	30	2.91E-07	31
NNU_29323	559	NNU_022682	627	0.92	83	9.02E-26	63
NNU_29324	948	NNU_007644	517	0.97	118	5.40E-50	107
NNU_29326	2821	NNU_026347	2289	0.87	183	7.55E-53	113
NNU_29330	744	NNU_011498	2767	0.95	421	0	354
NNU_29332	3515	NNU_025786	2578	0.83	364	1.15E-91	183
NNU_29332	3515	NNU_006709	3256	0.84	318	9.02E-83	167
NNU_29332	3515	NNU_024899	3722	0.83	156	2.71E-28	69
NNU_29332	3515	NNU_006009	2228	0.82	141	2.73E-23	60
NNU_29332	3515	NNU_001676	1042	0.88	77	9.89E-18	50
NNU_29332	3515	NNU_014577	3299	0.85	87	1.66E-15	46
NNU_29335	1130	NNU_001221	2794	0.93	97	3.07E-33	77
NNU_29335	1130	NNU_014549	447	1.00	30	1.14E-07	31
NNU_29344	1308	NNU_010871	501	0.90	134	9.78E-44	96
NNU_29345	9345	NNU_007455	543	0.95	58	2.64E-17	50
NNU_29350	6114	NNU_004520	2153	0.81	651	6.68E-146	281
NNU_29351	3189	NNU_017421	4857	0.95	376	1.56E-169	323
NNU_29351	3189	NNU_001329	4100	0.79	235	1.46E-35	82
NNU_29351	3189	NNU_025289	1250	0.81	124	1.93E-19	53

NNU_29351	3189	NNU_020222	482	0.94	48	3.25E-12	40
NNU_29352	793	NNU_003938	1461	0.89	140	5.85E-44	96
NNU_29361	1739	NNU_015423	1853	0.90	403	2.43E-145	279
NNU_29361	1739	NNU_005718	985	0.80	139	2.25E-21	56
NNU_29362	1305	NNU_005563	1350	0.84	631	1.38E-161	308
NNU_29364	938	NNU_002559	3905	0.89	103	1.98E-29	70
NNU_29366	130	NNU_014546	1752	0.95	129	1.04E-53	112
NNU_29366	130	NNU_026531	427	0.96	51	5.09E-17	46
NNU_29366	130	NNU_026530	621	0.94	49	3.07E-14	41
NNU_29367	450	NNU_017839	1534	0.82	272	5.29E-62	128
NNU_29367	450	NNU_014546	1752	0.94	148	1.14E-58	122
NNU_29367	450	NNU_026530	621	0.93	152	1.48E-57	120
NNU_29367	450	NNU_014547	303	0.93	146	3.20E-54	114
NNU_29368	1880	NNU_019044	1572	0.84	1453	0	723
NNU_29370	683	NNU_019099	845	0.86	440	1.60E-128	248
NNU_29370	683	NNU_019100	1079	0.81	244	2.32E-47	102
NNU_29370	683	NNU_019101	295	0.93	140	2.30E-52	111
NNU_29371	4177	NNU_020395	3471	0.90	215	2.34E-74	152
NNU_29372	899	NNU_015767	492	0.90	62	5.37E-15	44
NNU_29372	899	NNU_004531	810	0.93	54	1.93E-14	43
NNU_29376	1537	NNU_015680	1016	0.86	479	4.65E-142	273
NNU_29376	1537	NNU_015681	402	0.90	311	4.85E-112	219
NNU_29378	1501	NNU_014059	3731	0.94	246	6.21E-101	199
NNU_29384	1744	NNU_020613	1272	0.97	71	2.24E-26	65
NNU_29387	730	NNU_008816	1634	0.90	166	1.47E-54	115
NNU_29388	644	NNU_016191	5081	0.99	76	8.01E-32	74
NNU_29388	644	NNU_019690	4259	0.94	76	3.75E-25	62
NNU_29389	2270	NNU_010348	1364	0.90	76	1.37E-19	53
NNU_29391	1905	NNU_007198	2560	0.93	44	3.23E-10	36
NNU_29392	1628	NNU_001966	2250	0.96	134	2.59E-55	117
NNU_29392	1628	NNU_017023	807	0.95	134	1.20E-53	114
NNU_29392	1628	NNU_024752	1557	0.94	134	5.60E-52	111
NNU_29396	1625	NNU_001966	2250	1.00	51	3.51E-19	52
NNU_29396	1625	NNU_017023	807	0.96	51	7.59E-16	46
NNU_29396	1625	NNU_024752	1557	0.96	51	7.59E-16	46
NNU_29398	1356	NNU_005030	1311	0.80	410	3.47E-78	158
NNU_29399	734	NNU_020099	2018	0.86	148	2.53E-37	84

Table S3. Comparison of the expression of 14 common genes between our study and the previous study of Cheng et al. [10] who analyzed transcript profiling of lotus rhizome by sequence tag counting technology

Gene name	Gene ID	R [1]	P value	Significance [2]
APETALA AP2	NNU_17043	0.99	0.009	**
Cycling Dof Factor DOF	NNU_12021	0.95	0.02	*
Granule-Bound Starch Synthase GBSS	NNU_07282	0.62	0.57	N
MADS-box transcription factor AGL8	NNU_04430	0.44	0.71	N
Glucose pyrophosphorylase AGPase	NNU_11690	0.21	0.86	N
GA 20-oxidase	NNU_07907	0.14	0.9	N
Zinc finger CONSTANS-like protein COL	NNU_22255	0.07	0.96	N
Calmodulin-like protein	NNU_17533	-0.01	0.99	N
GIGANTEA GI	NNU_20074	-0.4	0.75	N
Sucrose synthase SUS	NNU_05767	-0.4	0.74	N
Phytochrome B PHYB	NNU_08548	-0.98	0.11	N
Lipoxygenase	NNU_06221	-0.99	0.006	**
BEL1-like HD transcription factor BEL1	NNU_05289	-0.99	0.003	*

[1] The correlation coefficient of the expression between the two study;

[2] The significant level: N, no significance; *, $P = 0.05$; **, $P = 0.01$.

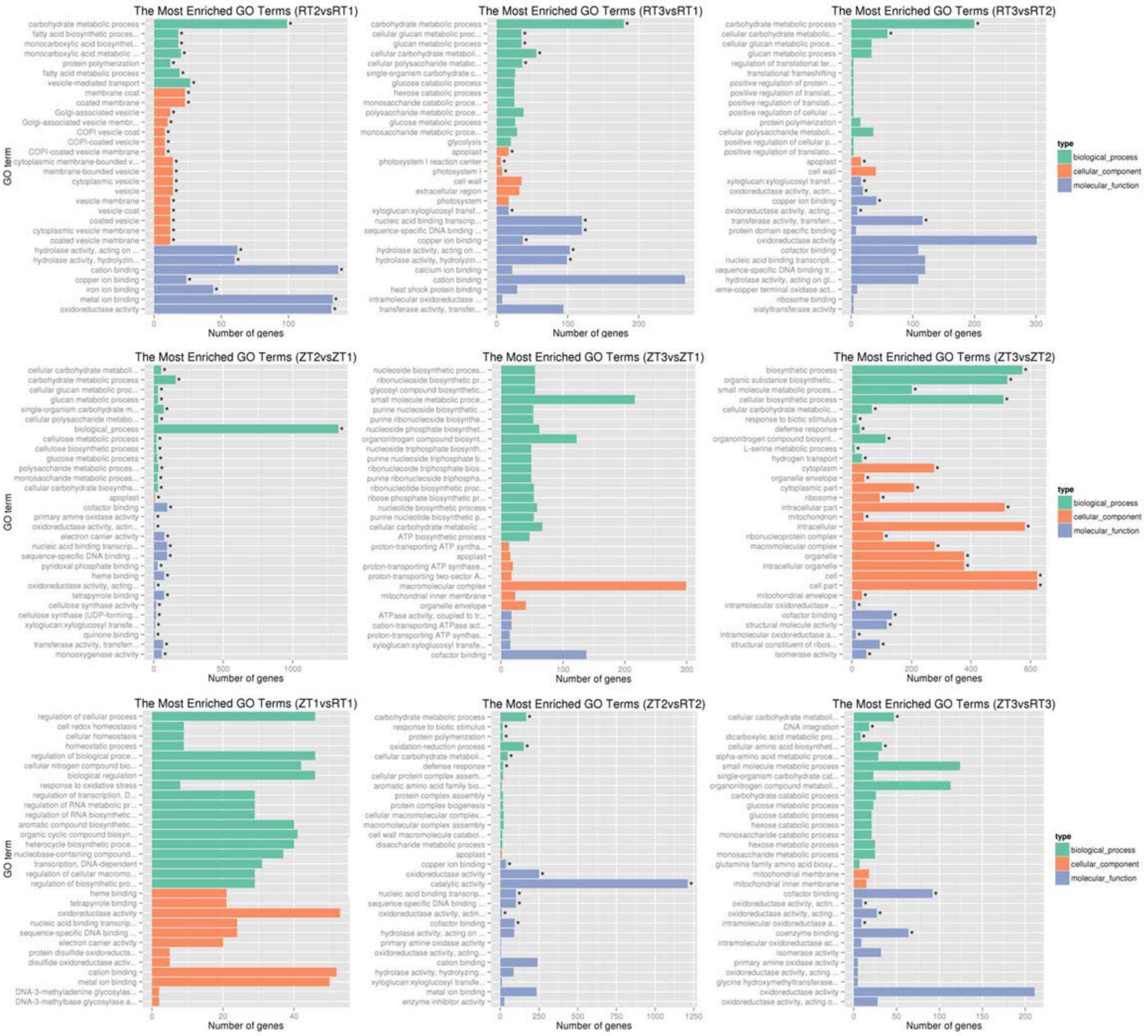

Figure S1. GO terms significantly enriched in DEGs in comparisons of RT1, RT2, RT3, ZT1, ZT2, and ZT3 libraries.

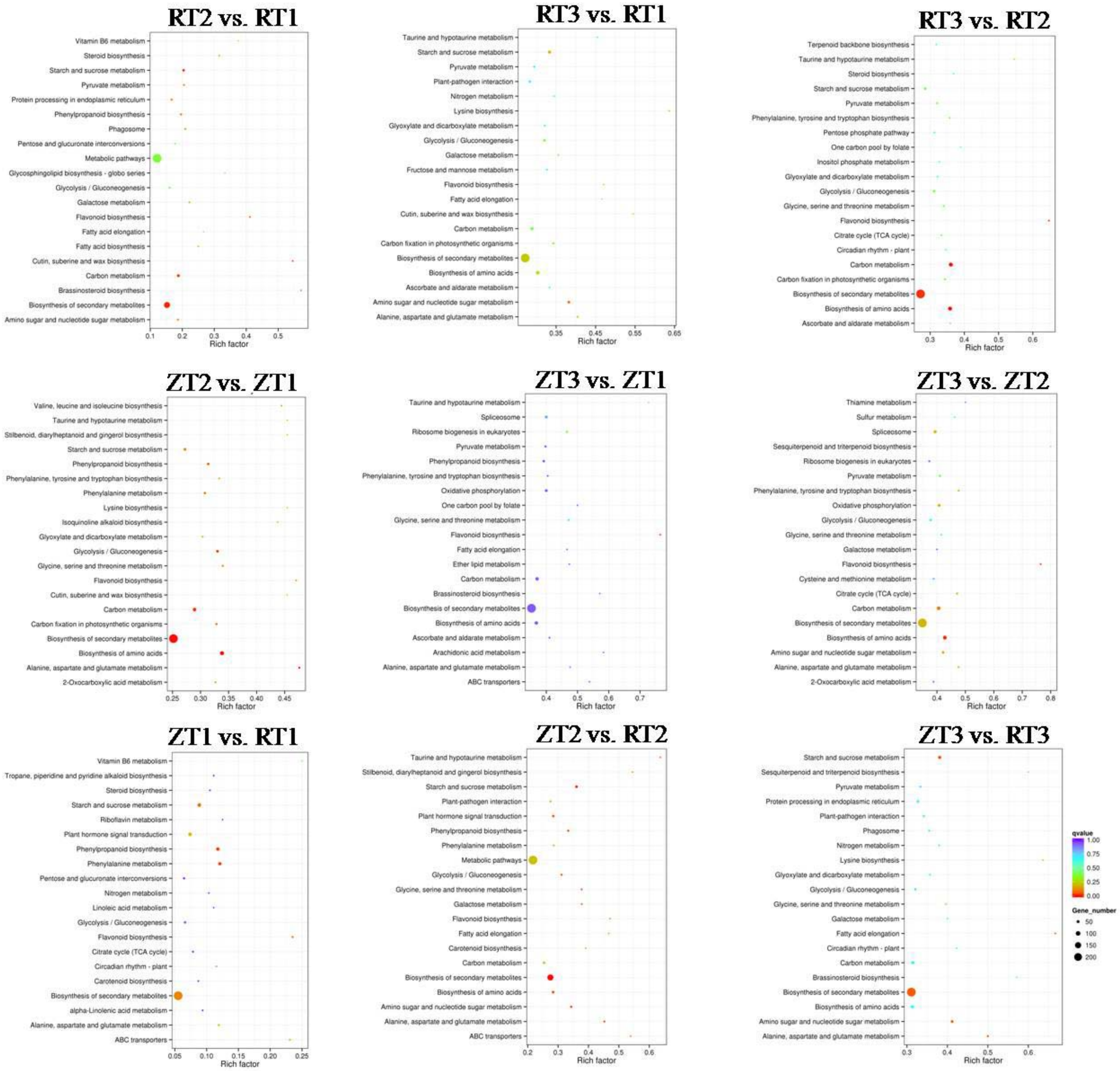

Figure S2. KEGG pathways significantly enriched in DEGs in comparisons of RT1, RT2, RT3, ZT1, ZT2, and ZT3 libraries.

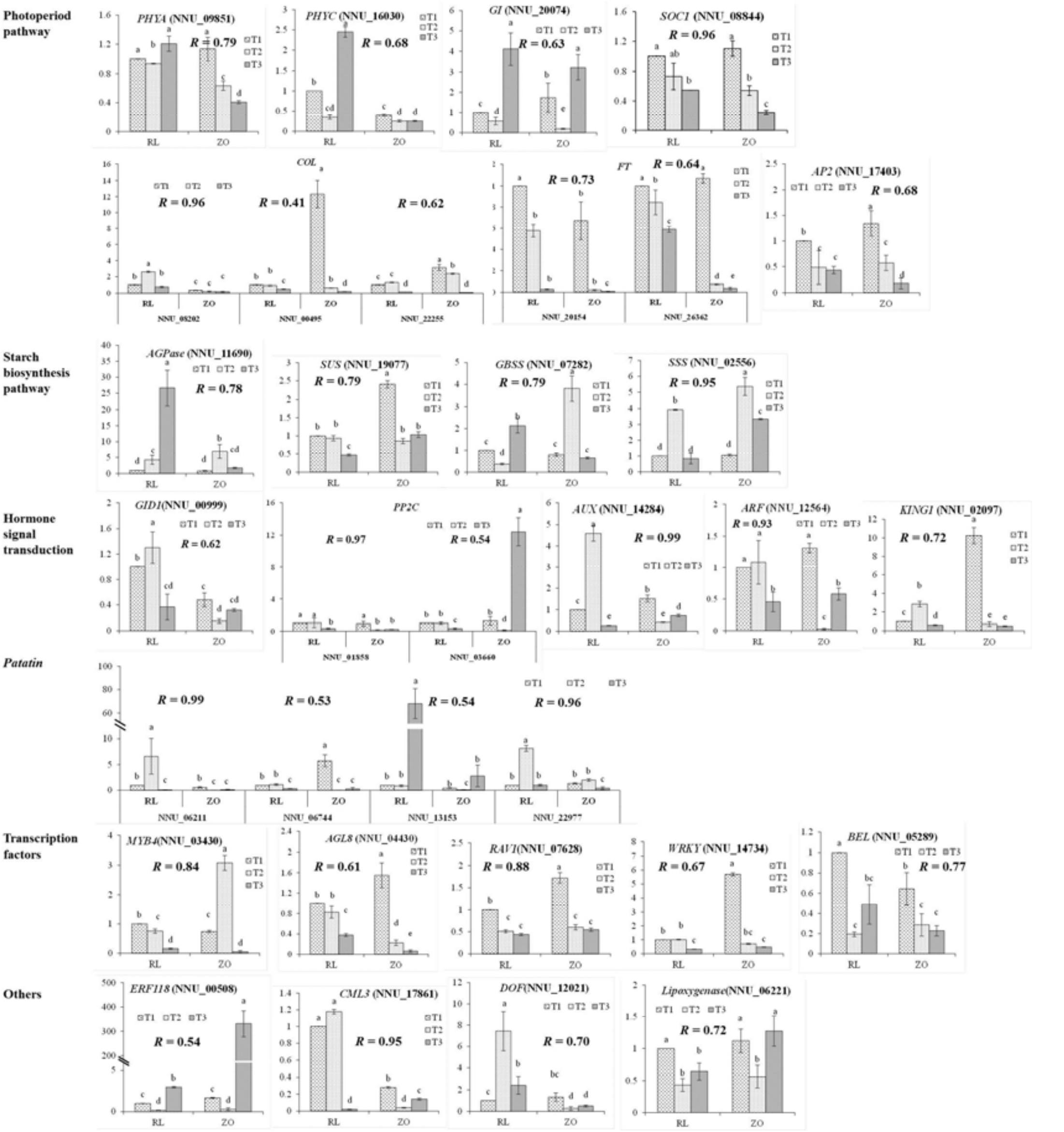

Figure S3. Real-time quantitative RT-qPCR validation of 31 genes expressed at the three stages, T1, T2, and T3, between the two cultivars, 'RL' and 'ZO'. The left y-axis indicates relative gene expression levels determined by RT-qPCR. Data were normalized against a lotus *β-actin* gene (NNU_24864) and analyzed using the $2^{-\Delta\Delta C}$T Method. The expression values were adjusted by setting the expression of RT1 to be 1 for each gene. All RT-qPCRs for each gene used three biological replicates, with three technical replicates per experiment; the error bars indicate SE. Different lower case letter (a, b, c, d, e) indicates the significant difference among six treatments at *P* = 0.05. The correlation coefficient (*R*) between the RT-qPCR and RNA-Seq data is shown for each gene.
